# Supplementary material for: Comparative analysis of Parkinson’s and inflammatory bowel disease gut microbiomes reveals shared butyrate-producing bacteria depletion
Source: NPJ Parkinsons Dis. 2025 Mar 19;11:50. doi: 10.1038/s41531-025-00894-4 (PMC11923181; doi:10.1038/s41531-025-00894-4)
Supplement: Supplementary file 1 — Supplementary Material [file 41531_2025_894_MOESM1_ESM.pdf]

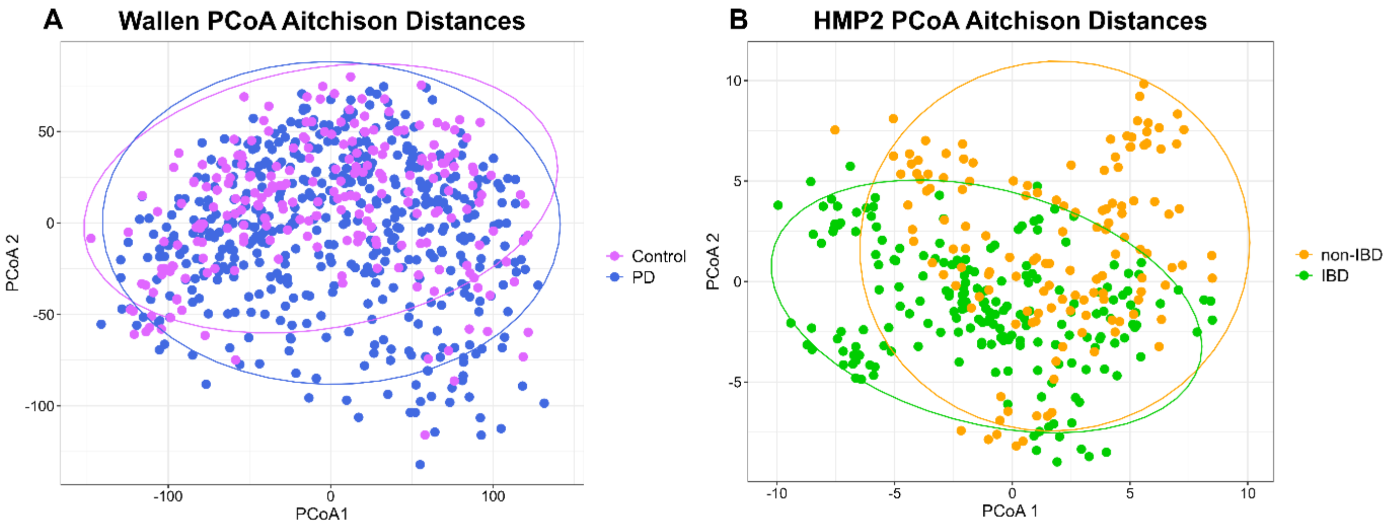

Supplementary Figure 1. Wallen PD and HMP2 IBD PCoA. (A) Principal coordinate analysis (PCoA) using Aitchison distances between 490 PD (blue) and 234 neurologically healthy control (pink) metagenomes with all species demonstrated PD dispersion was significantly different than Control by PERMANOVA (*p*=0.001) and PERMDISP (*p*=0.001). (B) PCoA using Aitchison distances between 198 IBD and 139 nonIBD metagenomes with all species demonstrated IBD dispersion was significantly different than nonIBD by PERMANOVA (*p*=0.001) but not by PERMDISP (*p*=0.832).

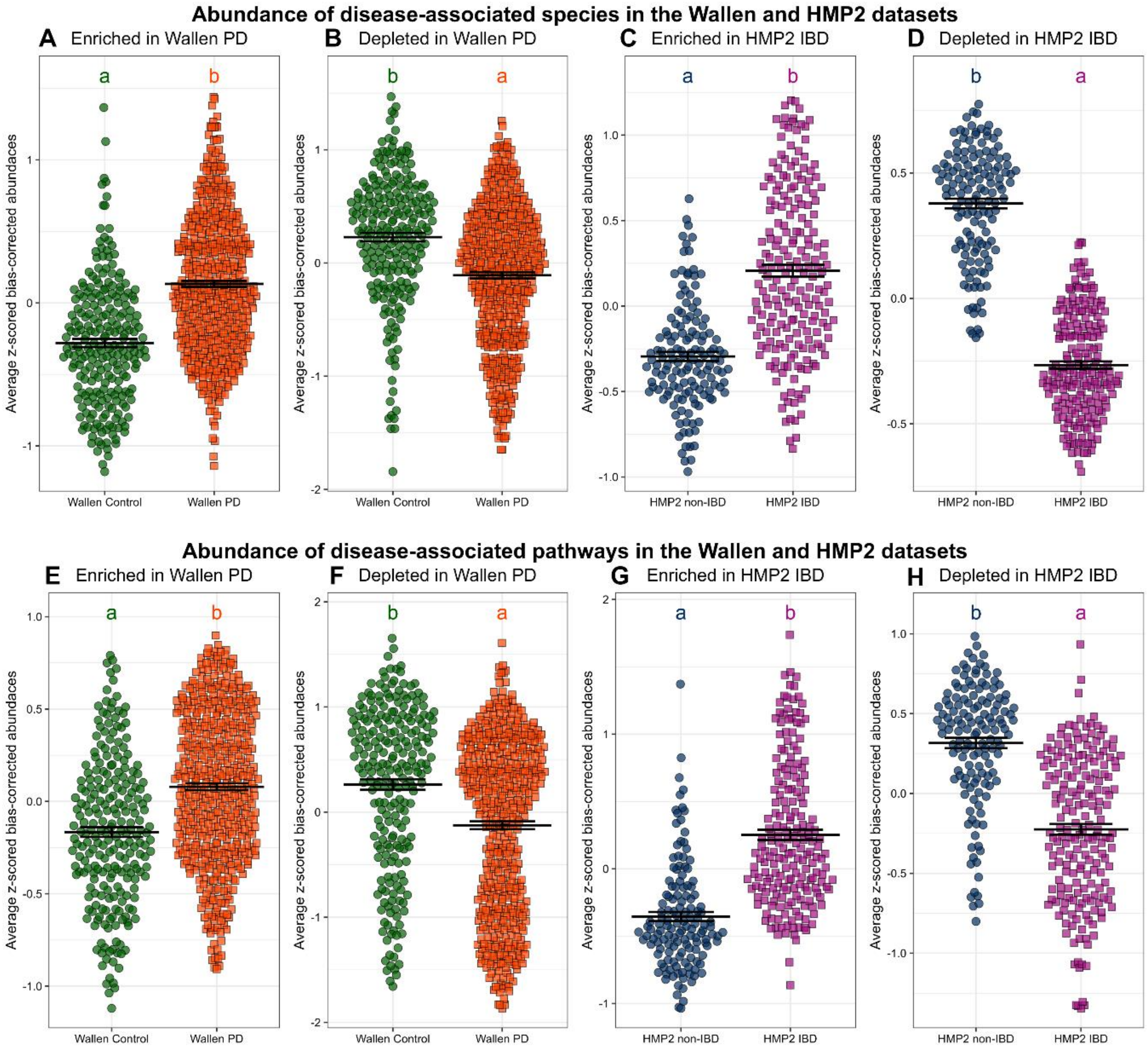

Supplementary Figure 2. Species and pathway module analysis quality control comparing Wallen PD and HMP2 IBD to their own modules. Quality control was performed by calculating module scores within in the Wallen and HMP2 datasets, ensuring that the abundance of features match the movement seen in the feature lists created initially. (A-B) The abundance of significant species from Wallen PD within the Wallen PD cohorts are plotted. (C-D) The abundance of significant species from HMP2 IBD within the HMP2 IBD cohorts are plotted. Modules are denoted by the title on each individual plot. Significantly enriched and depleted species were extracted into separate feature lists from the ANCOM-BC2 analyses of the Wallen and HMP2 datasets. Calculated module scores were comprised of the average abundance of those species found within each dataset respectively. One-way ANOVAs were performed followed by calculation of estimated marginal means and pairwise comparisons. Compact letter display was used to display pairwise comparisons, as letters that are different from each other are significantly different ($p < 0.05$). (E-F) The abundance of significant MetaCyc pathways from Wallen PD within the Wallen PD cohorts are plotted. (G-H) The abundance of significant MetaCyc pathways from HMP2 IBD within the HMP2 IBD cohorts are plotted. Statistical methods were identical as described above, but the feature lists were comprised of significantly enriched and depleted MetaCyc pathways from the ANCOM-BC2 analyses of the Wallen and HMP2 datasets, and calculated module scores were comprised of the average abundance of those pathways found within those feature lists.

Supplementary Table 1: UFPF all species-level ANCOM-BC2 output

Differential abundances of species in PD (N = 54) vs. IBD (N = 26) vs. Healthy Control (N=16) were tested using ANCOM-BC2, while adjusting for covariates (diagnosis and total sequence count per sample). Shown are all 233 species that were detected by MetaPhlAn, however, only species that were present in at least 10% of samples were included in ANCOM-BC2 analysis. LFC: indicates the log fold change compared to healthy control or in the case of lfc_PDvsIBD it is the log fold change of PD compared to IBD. SE: standard error; Pval: the uncorrected, two-sided P-value reported by ANCOM-BC2; Adj. Pval aka q-value from the false discovery rate, i.e., multiple-testing corrected significance q-value, calculated using the Benjamini-Hochberg method, alpha =0.05.

species	lfc_IBD	lfc_PD	lfc_PDvsIBD	se_IBD	se_PD	se_PDvsIBD	pval_IBD	pval_PD	pval_PDvsIBD	adj_pval_IBD	adj_pval_PD	adj_pval_PDvsIBD
Faecalimonas_umbili	6.0161365	0.7888483	-5.2272882	1.3052196	1.1612559	1.3824636	4.04E-06	0.4969439	0.0001561	0.0028243	1	0.1089661
Corynebacterium_am	0.4895925	0.8200546	0.3304621	1.6901784	1.5052247	1.6240207	1	1	1	1	1	1
GGB2945_SGB3917	0.4587373	1.0475459	0.5888086	1.3107698	1.1662148	1.3858435	1	1	1	1	1	1
Fenollaria_timonensis	-1.3192452	2.2281631	3.5474083	1.7083514	1.5214628	1.6357506	1	1	1	1	1	1
Ezakiella_SGB6726	-1.4956695	0.6594008	2.1550703	1.546379	1.376735	1.5321767	1	1	1	1	1	1
Varibaculum_cambrie	-3.5968994	1.5098785	5.106778	1.663348	1.4812509	1.6067515	1	1	1	1	1	1
Porphyromonas_SGB1	0.4965208	0.9003521	0.4038314	1.3629005	1.2127925	1.4177443	1	1	1	1	1	1
Clostridiales_bacteriu	0.7859053	1.3157106	0.5298053	1.1863867	1.0550932	1.3108564	1	1	1	1	1	1
Prevotella_buccalis	-1.1057687	2.3370498	3.4428185	1.6399326	1.4603285	1.5917288	1	1	1	1	1	1
Collinsella_aerofacier	1.5118525	0.5860729	-0.9257796	1.6840427	1.4997423	1.6200664	1	1	1	1	1	1
Peptoniphilus_grosser	-1.7757682	2.2714016	4.0471698	1.592698	1.4181227	1.5615656	1	1	1	1	1	1
Corynebacterium_aur	1.9492624	1.0088668	-0.9403955	1.736235	1.5463774	1.6537988	1	1	1	1	1	1
Mobiluncus_SGB1548	0.0472424	4.2829094	4.235667	1.5429996	1.3737153	1.53004	1	1	1	1	1	1
GGB9715_SGB15265	-1.8138202	-1.9872281	-0.1734079	1.3608116	1.210926	1.4164606	1	1	1	1	1	1
Porphyromonas_SGB1	-1.2816158	0.3577528	1.6393686	1.4980194	1.3335239	1.5017013	1	1	1	1	1	1
Alterileibacterium_ma	0.5097056	2.25539	1.7456844	1.4596436	1.2992338	1.4776742	1	1	1	1	1	1
Peptococcus_niger	-0.8561613	1.304623	2.1607843	1.478783	1.3163355	1.4896398	1	1	1	1	1	1
Gemmiger_formicilis	-4.1615977	-1.3971224	2.7644754	1.6814382	1.4974151	1.6183887	1	1	1	1	1	1
Peptoniphilus_sp_Mai	-2.0610515	0.749712	2.8107634	1.6010319	1.4255694	1.5668735	1	1	1	1	1	1
GGB1456_SGB2019	-0.3163443	1.8208345	2.1371787	1.5196149	1.3528202	1.5152835	1	1	1	1	1	1
Oscillibacter_sp_ER4	-1.9539848	-1.5218407	0.4321441	1.4667995	1.3056278	1.4821438	1	1	1	1	1	1
Peptoniphilus_pacaer	-1.0377192	0.1699936	1.2077128	1.35523	1.2059389	1.4130328	1	1	1	1	1	1
Alistipes_putredinis	-2.7203899	-0.7705382	1.9498517	1.6705687	1.4877028	1.6113933	1	1	1	1	1	1
Ruminococcus_bicirc	-1.8209086	-1.7574328	0.0634758	1.651547	1.4707063	1.5991746	1	1	1	1	1	1
Evtepia_gabavorous	-0.7572542	0.2956509	1.0529051	1.6714743	1.4885121	1.6119758	1	1	1	1	1	1
Blautia_obeum	-3.782755	-0.9831344	2.7996206	1.5886107	1.4144706	1.5589645	1	1	1	1	1	1
Faecalibacterium_pra	-5.2741822	-1.4605881	3.8135941	1.6783812	1.4946836	1.6164204	1	1	1	1	1	1
Phascolarctobacteriu	0.6381992	1.3067303	0.668531	1.6280993	1.4497551	1.5841544	1	1	1	1	1	1
GGB9512_SGB14909	-0.9775969	-0.815956	0.1616409	1.5356434	1.3671422	1.5253926	1	1	1	1	1	1
Porphyromonas_benn	-2.2813337	0.6122176	2.8935514	1.6305987	1.4519883	1.5857532	1	1	1	1	1	1
Bacteroides_uniformis	-0.5999902	0.4895839	1.0895741	1.5723269	1.3999203	1.5486167	1	1	1	1	1	1
Bifidobacterium_adol	-1.3816301	-0.8467609	0.5348692	1.5482468	1.3784039	1.533358	1	1	1	1	1	1
Wujia_chipingensis	-1.2826377	-1.3560416	-0.0734039	1.2240043	1.0886976	1.3333695	1	1	1	1	1	1

species	lfc_IBD	lfc_PD	lfc_PDvsIBD	se_IBD	se_PD	se_PDvsIBD	pval_IBD	pval_PD	pval_PDvsIBD	adj_pval_IBD	adj_pval_PD	adj_pval_PDvsIBD
Dorea_longicatena	-2.3659936	-2.7985846	-0.432591	1.709715	1.5226812	1.6366318	1	1	1	1	1	1
Anaerobutyricum_hall	-1.3096804	-0.9333492	0.3763312	1.683247	1.4990313	1.6195538	1	1	1	1	1	1
Lagierella_massiliensi	0.6744653	0.728596	0.0541307	1.1007746	0.9786285	1.2601096	1	1	1	1	1	1
Clostridiaceae_unclas	0.0624665	-0.2781503	-0.3406168	1.3263489	1.1801342	1.3953476	1	1	1	1	1	1
Acidaminococcus_int	1.4381117	0.379551	-1.0585606	1.4676169	1.3063582	1.4826547	1	1	1	1	1	1
Barnesiella_intestinih	-0.4058771	-0.5052484	-0.0993713	1.4922223	1.3283439	1.4980627	1	1	1	1	1	1
Adlercreutzia_equolifa	0.9075379	1.224689	0.3171511	1.517494	1.3509251	1.5139477	1	1	1	1	1	1
Mediterraneibacter_fa	-2.132381	-2.8306105	-0.6982295	1.6673336	1.4848122	1.6093131	1	1	1	1	1	1
Blautia_faecis	-2.4809301	-1.1446077	1.3363223	1.6143328	1.4374542	1.5753574	1	1	1	1	1	1
Candidatus_Cibioniba	-3.0007533	-0.8450526	2.1557008	1.7035063	1.5171335	1.6326207	1	1	1	1	1	1
Eubacterium_rectale	-3.1574529	-1.9078139	1.2496391	1.8188202	1.6201687	1.7075996	1	1	1	1	1	1
Faecalicatena_fissica	-1.4222475	-1.8386604	-0.416413	1.3272063	1.1809002	1.3958714	1	1	1	1	1	1
Fusicatenibacter_sac	-3.207854	-3.36981	-0.1619559	1.7836956	1.5887844	1.6846557	1	1	1	1	1	1
Ruminococcus_bromi	0.3076943	1.0357286	0.7280344	1.8899191	1.6836956	1.7543089	1	1	1	1	1	1
Agathobaculum_butyr	-2.8321943	-3.0994985	-0.2673042	1.5438982	1.3745182	1.530608	1	1	1	1	1	1
Phocaeicola_vulgatus	0.5370712	0.1941399	-0.3429312	1.7241534	1.5355823	1.6459713	1	1	1	1	1	1
Blautia_sp_MCC283	-0.6911495	-1.8543379	-1.1631884	1.1088865	0.9858725	1.2648917	1	1	1	1	1	1
Brotolimicola_acetigi	-1.9443045	-1.9137546	0.0305499	1.4310999	1.2737293	1.4598953	1	1	1	1	1	1
Eubacteriales_Family_	0.1491492	1.7852751	1.6361259	1.4740955	1.312147	1.4867059	1	1	1	1	1	1
GGB9715_SGB15260	-0.6034479	-0.4343862	0.1690617	1.3094218	1.1650104	1.3850223	1	1	1	1	1	1
Olegusella_massiliens	-0.381188	0.270843	0.652031	1.2788413	1.1376885	1.3664437	1	1	1	1	1	1
GGB4277_SGB5832	-2.8810828	0.7967607	3.6778435	1.6055445	1.4296015	1.5697501	1	1	1	1	1	1
Phocaeicola_dorei	-2.1344565	-0.8179695	1.316487	1.6937936	1.508455	1.6263521	1	1	1	1	1	1
Ruminococcus_lactar	0.2795743	-0.5479687	-0.8275429	1.3444123	1.1962734	1.4063986	1	1	1	1	1	1
Alistipes_indistinctus	-0.3145116	-1.0099969	-0.6954853	1.076291	0.9567661	1.2457036	1	1	1	1	1	1
Dorea_formicigenerar	-2.3659285	-2.3829325	-0.017004	1.6184748	1.4411552	1.5780025	1	1	1	1	1	1
Oscillibacter_valericig	-1.5076201	-0.5116764	0.9959437	1.4703584	1.3088078	1.4843685	1	1	1	1	1	1
Oscillospiraceae_bac	-2.6091954	-1.3898856	1.2193098	1.6147539	1.4378304	1.5756262	1	1	1	1	1	1
Oliverpabstia_intestin	-1.6238761	-1.9911668	-0.3672908	1.6056313	1.4296791	1.5698055	1	1	1	1	1	1
Blautia_wexlerae	-1.4469471	-1.9154511	-0.4685039	1.8845672	1.6789138	1.7507809	1	1	1	1	1	1
Alistipes_communis	0.6930442	0.684347	-0.0086972	1.2010803	1.0682189	1.3196335	1	1	1	1	1	1
Alistipes_shahii	-1.1564495	0.5981036	1.7545531	1.4852876	1.3221475	1.4937143	1	1	1	1	1	1
Blautia_massiliensis	-2.7910106	-2.5501676	0.240843	1.7609134	1.5684281	1.6698224	1	1	1	1	1	1
Coprococcus_comes	-2.7878577	-2.6015776	0.18628	1.5216192	1.3546111	1.5165463	1	1	1	1	1	1
Parabacteroides_mer	1.948345	2.1239338	0.1755888	1.5432196	1.3739119	1.5301791	1	1	1	1	1	1
Lawsonella_SGB3665	-1.0087383	2.1913388	3.2000771	1.5625679	1.3912003	1.5424265	1	1	1	1	1	1
Phocaeicola_massilie	-3.1061167	-3.0620295	0.0440872	1.4665541	1.3054085	1.4819904	1	1	1	1	1	1
Ruminococcus_torqu	-0.576473	-0.0718115	0.5046615	1.7429039	1.5523362	1.6581243	1	1	1	1	1	1
Alistipes_onderdonkii	-0.2024393	0.0113973	0.2138366	1.6164898	1.4393815	1.5767347	1	1	1	1	1	1
Negativibacillus_mas	-0.4354665	-1.0385427	-0.6030761	1.3244853	1.1784691	1.3942094	1	1	1	1	1	1
GGB3433_SGB4573	1.3813393	1.0801683	-0.301171	1.4390528	1.2808354	1.4648409	1	1	1	1	1	1

species	lfc_IBD	lfc_PD	lfc_PDvsIBD	se_IBD	se_PD	se_PDvsIBD	pval_IBD	pval_PD	pval_PDvsIBD	adj_pval_IBD	adj_pval_PD	adj_pval_PDvsIBD
Bacteroides_intestina	0.2240072	0.7614195	0.5374123	1.3725631	1.2214259	1.4236877	1	1	1	1	1	1
Escherichia_coli	5.7305709	-0.0589693	-5.7895402	1.8398252	1.6389368	1.7213627	1	1	1	1	1	1
Dysosmobacter_welb	-1.2273612	0.8926375	2.1199987	1.5709452	1.3986858	1.5477398	1	1	1	1	1	1
Bacteroides_stercoris	-1.0575551	-0.4158232	0.6417319	1.8386691	1.6379038	1.7206043	1	1	1	1	1	1
Parabacteroides_dista	0.1477542	-0.9575549	-1.1053092	1.5424613	1.3732343	1.5296997	1	1	1	1	1	1
Paraprevotella_clara	1.270002	0.3948004	-0.8752016	1.2362471	1.0996348	1.3407269	1	1	1	1	1	1
GGB3167_SGB4181	2.8871511	2.0140668	-0.8730843	1.3071616	1.1629911	1.3836459	1	1	1	1	1	1
Bacteroides_faecis	2.6655974	0.6143609	-2.0512365	1.1925665	1.0606135	1.3145453	1	1	1	1	1	1
Monoglobus_pectinily	-0.1175752	-1.1346081	-1.0170329	1.3122398	1.1675282	1.3867392	1	1	1	1	1	1
Streptococcus_parasa	2.249282	-1.2061074	-3.4553893	1.1747203	1.0446721	1.3039025	1	1	1	1	1	1
Bacteroides_finegoldi	0.3093595	-0.4650843	-0.7744438	1.3550198	1.2057511	1.4129038	1	1	1	1	1	1
GGB51647_SGB4348	-1.11833	-2.3088688	-1.1905387	1.2875113	1.1454346	1.3717012	1	1	1	1	1	1
Clostridiales_bacteriu	-0.6786009	-0.3377979	0.340803	1.4720512	1.3103204	1.4854271	1	1	1	1	1	1
Eisenbergiella_tayi	1.3866972	2.7090615	1.3223644	1.4600536	1.2996002	1.4779301	1	1	1	1	1	1
Corynebacterium_sim	0.4673228	-0.8838292	-1.351152	1.2753295	1.134551	1.3643163	1	1	1	1	1	1
Blautia_caecimuris	4.0010046	0.2961951	-3.7048094	1.6168307	1.4396862	1.5769524	1	1	1	1	1	1
Lawsonibacter_homin	1.3565763	1.4832525	0.1266761	1.2200755	1.0851878	1.3310116	1	1	1	1	1	1
Bacteroides_ovatus	2.1703102	1.4442357	-0.7260745	1.6314782	1.4527742	1.586316	1	1	1	1	1	1
Anaerostipes_hadrus	-2.1755751	-1.9209646	0.2546105	1.7798124	1.5853147	1.6821247	1	1	1	1	1	1
Bacteroides_caccae	-1.6984105	-1.4360631	0.2623474	1.6714313	1.4884736	1.6119481	1	1	1	1	1	1
Faecalibacterium_sp_	0.2069817	-0.7173652	-0.9243469	1.3434636	1.1954258	1.4058174	1	1	1	1	1	1
Ruthenibacterium_lac	-0.6648056	1.54962	2.2144257	1.5849628	1.411211	1.5566444	1	1	1	1	1	1
Eggerthella_lenta	1.8494427	1.8338734	-0.0155693	1.6296706	1.4511591	1.5851595	1	1	1	1	1	1
Bilophila_wadsworthia	0.1238184	2.1270962	2.0032778	1.4564255	1.2963583	1.4756658	1	1	1	1	1	1
Clostridium_sp_AF36_	1.3175034	1.1515301	-0.1659733	1.1597052	1.0312602	1.2949714	1	1	1	1	1	1
Enterocloster_clostric	3.6536821	1.2363347	-2.4173475	1.2624542	1.123048	1.3565277	1	1	1	1	1	1
Clostridium_scindens	2.3288441	0.6609184	-1.6679257	1.327578	1.1812324	1.3960985	1	1	1	1	1	1
Roseburia_inulinivora	-0.5091664	-1.1831234	-0.673957	1.4537188	1.2939398	1.4739773	1	1	1	1	1	1
Parasutterella_excrem	0.8454472	-0.8407988	-1.686246	1.4214525	1.2651092	1.4539044	1	1	1	1	1	1
Anaerotruncus_massi	2.4622622	1.7030055	-0.7592567	1.0897364	0.9687717	1.2536099	1	1	1	1	1	1
Akkermansia_mucinip	0.7562743	1.1571775	0.4009031	1.8175964	1.6190752	1.7067987	1	1	1	1	1	1
Blautia_hansenii	3.8932307	0.8724669	-3.0207638	1.234269	1.0978677	1.3395372	1	1	1	1	1	1
Clostridium_symbiosu	2.5160726	2.4765106	-0.0395621	1.4218997	1.2655087	1.454182	1	1	1	1	1	1
Enterocloster_hominis	-2.243698	-1.2230395	1.0206585	1.4540579	1.2942428	1.4741888	1	1	1	1	1	1
Blautia_hydrogenotro	1.0773985	-0.4139667	-1.4913652	1.337499	1.1900965	1.4021652	1	1	1	1	1	1
Eubacterium_ramulus	0.0217546	-1.2739367	-1.2956913	1.4155862	1.2598675	1.4502661	1	1	1	1	1	1
Blautia_producta	3.7359695	0.8289852	-2.9069843	1.2697193	1.1295387	1.3609205	1	1	1	1	1	1
Intestinimonas_massi	1.9474721	1.4024881	-0.544984	1.2625039	1.1230924	1.3565577	1	1	1	1	1	1
Ruminococcus_gnavu	0.7440217	-2.1341115	-2.8781332	1.7591682	1.5668688	1.6686878	1	1	1	1	1	1
Enterocloster_bolteae	2.3508603	2.7896514	0.4387911	1.5486238	1.3787407	1.5335965	1	1	1	1	1	1
Lachnospira_eligens	-1.9708761	-1.2795383	0.6913378	1.3852602	1.2327708	1.4315122	1	1	1	1	1	1

species	lfc_IBD	lfc_PD	lfc_PDvsIBD	se_IBD	se_PD	se_PDvsIBD	pval_IBD	pval_PD	pval_PDvsIBD	adj_pval_IBD	adj_pval_PD	adj_pval_PDvsIBD
Neglectibacter_timon	1.2220834	-0.1677347	-1.3898181	1.3078401	1.1635972	1.384059	1	1	1	1	1	1
Roseburia_hominis	0.5386454	0.4883155	-0.0503298	1.2672637	1.1273448	1.3594351	1	1	1	1	1	1
Corynebacterium_tub	1.0866314	0.4967486	-0.5898828	1.2160131	1.0815587	1.3285753	1	1	1	1	1	1
Klebsiella_pneumonia	4.4149506	0.3027332	-4.1122174	1.3488427	1.2002319	1.4091142	1	1	1	1	1	1
Porphyromonas_ueno	-0.4831749	-0.5403156	-0.0571408	1.3310259	1.184313	1.3982057	1	1	1	1	1	1
Lawsonibacter_asacc	-0.1463397	-0.6552681	-0.5089285	1.3763835	1.2248394	1.4260403	1	1	1	1	1	1
Blautia_glucerasea	-2.0706042	-2.5883287	-0.5177245	1.3183326	1.1729718	1.3904541	1	1	1	1	1	1
Roseburia_intestinalis	-2.0683671	-2.9763057	-0.9079386	1.3856885	1.2331536	1.4317764	1	1	1	1	1	1
Roseburia_faecis	-0.6294234	-1.0930652	-0.4636418	1.6612215	1.4793508	1.6053853	1	1	1	1	1	1
Flavonifractor_plautii	-0.3400251	1.0716399	1.411665	1.5418147	1.3726566	1.5292911	1	1	1	1	1	1
Dialister_invisus	0.7409974	0.7733592	0.0323618	1.5668163	1.3949964	1.5451202	1	1	1	1	1	1
Lachnospira_pectinos	-1.1161505	-1.1601744	-0.0440239	1.4398686	1.2815643	1.4653486	1	1	1	1	1	1
Bifidobacterium_long	2.3978404	-0.2259191	-2.6237595	1.7384228	1.5483323	1.6552175	1	1	1	1	1	1
Eisenbergiella_massil	1.7323296	1.9208892	0.1885597	1.379511	1.2276339	1.4279673	1	1	1	1	1	1
Finegoldia_magna	-3.4497463	0.8285409	4.2782871	1.6013063	1.4258145	1.5670483	1	1	1	1	1	1
Streptococcus_saliva	0.6436729	-1.3548659	-1.9985388	1.4573723	1.2972043	1.4762566	1	1	1	1	1	1
Corynebacterium_pse	0.2302966	0.0618389	-0.1684578	1.142891	1.0162419	1.2849946	1	1	1	1	1	1
Enterocloster_aldene	2.3804316	1.4428627	-0.9375689	1.1385111	1.01233	1.2823998	1	1	1	1	1	1
Anaerococcus_prevot	1.2319187	0.1461355	-1.0857832	1.1023539	0.9800388	1.2610402	1	1	1	1	1	1
Clostridium_leptum	1.2608763	0.1181451	-1.1427312	1.3112217	1.1666186	1.3861188	1	1	1	1	1	1
Faecalibacterium_SG	-1.2445239	-1.4779698	-0.2334459	1.467889	1.3066013	1.4828247	1	1	1	1	1	1
GGB9342_SGB14306	-0.4726254	-0.0164509	0.4561745	1.3250556	1.1789787	1.3945577	1	1	1	1	1	1
GGB9708_SGB15234	-1.6813347	-0.2849192	1.3964155	1.4219405	1.2655452	1.4542072	1	1	1	1	1	1
GGB3653_SGB4964	-0.3879575	0.1724999	0.5604574	1.3681493	1.2174822	1.4209716	1	1	1	1	1	1
GGB36331_SGB1512	0.1243948	0.2917286	0.1673339	1.171061	1.0414034	1.301724	1	1	1	1	1	1
Coprococcus_catus	-1.8481004	-0.3611029	1.4869975	1.4011552	1.2469732	1.4413303	1	1	1	1	1	1
GGB9365_SGB14341	-0.4853299	-1.5043368	-1.0190069	1.2687055	1.128633	1.3603072	1	1	1	1	1	1
Blautia_luti	0.260111	-1.3343959	-1.5945069	1.2770833	1.1361179	1.3653786	1	1	1	1	1	1
Clostridiaceae_bacte	-2.6924728	-2.528014	0.1644588	1.4857912	1.3225975	1.4940299	1	1	1	1	1	1
GGB9730_SGB15291	-0.2846835	-1.0128292	-0.7281458	1.2400733	1.1030531	1.3430295	1	1	1	1	1	1
Faecalibacterium_sp_	-1.7133097	-1.9139932	-0.2006835	1.3094734	1.1650565	1.3850537	1	1	1	1	1	1
Clostridium_fessum	-1.4386224	-1.8860591	-0.4474367	1.3721001	1.2210123	1.4234027	1	1	1	1	1	1
Sutterella_wadsworth	-3.2361667	-1.938216	1.2979507	1.4789276	1.3164646	1.4897303	1	1	1	1	1	1
Lachnospiraceae_bac	-0.4484115	-0.8723095	-0.423898	1.4075326	1.2526715	1.4452766	1	1	1	1	1	1
GGB32900_SGB5344	-1.397172	-1.2117969	0.1853752	1.2846501	1.1428783	1.3699653	1	1	1	1	1	1
GGB9635_SGB15106	-0.4639457	-0.3538884	0.1100573	1.1921697	1.0602591	1.3143084	1	1	1	1	1	1
Lentihominibacter_fa	-0.1732764	0.4078951	0.5811715	1.2815909	1.1401451	1.3681102	1	1	1	1	1	1
Odoribacter_splanchn	-2.4636998	-1.0493859	1.4143139	1.3593996	1.2096644	1.4155931	1	1	1	1	1	1
Methanobrevibacter_	-1.9254864	-0.7946879	1.1307985	1.333412	1.1864448	1.3996647	1	1	1	1	1	1
Lachnospiraceae_bac	-1.343307	-2.2227441	-0.879437	1.3118861	1.1672122	1.3865237	1	1	1	1	1	1
GGB9760_SGB15373	0.1246186	-0.2865991	-0.4112177	1.2440392	1.1065961	1.3454176	1	1	1	1	1	1

species	lfc_IBD	lfc_PD	lfc_PDvsIBD	se_IBD	se_PD	se_PDvsIBD	pval_IBD	pval_PD	pval_PDvsIBD	adj_pval_IBD	adj_pval_PD	adj_pval_PDvsIBD
Desulfovibrio_fairfield	0.0847352	-0.4592106	-0.5439458	1.1522623	1.0246122	1.290552	1	1	1	1	1	1
GGB9699_SGB15216	-1.115429	-0.353027	0.7624021	1.3756229	1.2241599	1.4255718	1	1	1	1	1	1
Blautia_SGB4815	-1.8385333	-2.448254	-0.6097207	1.0872457	0.9665476	1.2521444	1	1	1	1	1	1
Clostridiaceae_bacte	-0.3656193	-0.5794263	-0.213807	1.1015663	0.9793355	1.2605761	1	1	1	1	1	1
Clostridium_sp_AM22	-0.246304	-1.4615142	-1.2152102	1.3079147	1.1636639	1.3841045	1	1	1	1	1	1
Lacrimispora_amygda	-0.9800341	-1.5979699	-0.6179358	1.3154508	1.1703971	1.3886965	1	1	1	1	1	1
GGB2980_SGB3962	0.6323235	0.3662553	-0.2660682	0.989726	0.8795012	1.194963	1	1	1	1	1	1
Dorea_sp_AF36_15AT	-2.5132597	-3.3592368	-0.8459771	1.2618416	1.1225007	1.3561576	1	1	1	1	1	1
GGB1215_SGB1581	0.2735184	1.8791247	1.6056062	1.4596578	1.2992465	1.4776831	1	1	1	1	1	1
Levyella_massiliensis	-3.0207659	0.4399947	3.4607606	1.8063561	1.6090319	1.6994476	1	1	1	1	1	1
Porphyromonas_sp_H	-1.0136894	0.8025429	1.8162323	1.547863	1.3780609	1.5331152	1	1	1	1	1	1
Prevotella_bivia	-0.6253055	0.1517808	0.7770863	1.3869313	1.234264	1.4325432	1	1	1	1	1	1
Parabacteroides_gold	0.9116541	0.8509886	-0.0606655	1.2112063	1.0772646	1.3256945	1	1	1	1	1	1
Peptoniphilus_urinima	0.4751745	0.7035144	0.2283399	1.259508	1.1204158	1.3547479	1	1	1	1	1	1
Clostridiales_bacteriu	0.4660014	1.812292	1.3462907	1.4375853	1.2795242	1.4639279	1	1	1	1	1	1
GGB3109_SGB4121	0.5294954	2.3677326	1.8382372	1.3681708	1.2175014	1.4209848	1	1	1	1	1	1
Bacteroides_thetaiota	-1.9350796	0.6012742	2.5363538	1.5165227	1.3500572	1.5133361	1	1	1	1	1	1
GGB9480_SGB14874	0.3146234	1.2229377	0.9083143	1.4500436	1.2906559	1.4716858	1	1	1	1	1	1
Anaerococcus_sp_Ma	0.7907809	0.4009185	-0.3898624	1.1403214	1.0139468	1.2834721	1	1	1	1	1	1
Clostridium_innocuur	2.4590812	2.6371672	0.178086	1.4905569	1.3268559	1.497018	1	1	1	1	1	1
Prevotella_corporis	-1.5696003	1.5790658	3.1486661	1.744605	1.5538562	1.6592282	1	1	1	1	1	1
Anaerococcus_obesie	-0.8378754	0.6790258	1.5169012	1.4324785	1.2749611	1.4607522	1	1	1	1	1	1
Hungatella_hatheway	0.2518833	1.4691797	1.2172964	1.4579626	1.2977318	1.476625	1	1	1	1	1	1
Streptococcus_therm	1.4179922	-0.5906164	-2.0086086	1.5214329	1.3544446	1.5164289	1	1	1	1	1	1
Bifidobacterium_anim	0.7062626	0.2616142	-0.4446483	1.19617	1.0638325	1.316698	1	1	1	1	1	1
Bacteroides_eggerthii	0.3195279	-1.5904505	-1.9099784	1.4797946	1.3172393	1.4902731	1	1	1	1	1	1
Anaerobutyricum_soe	-0.7059133	-0.0580385	0.6478748	1.4626912	1.301957	1.4795771	1	1	1	1	1	1
Bacteroides_xylanisol	0.2649655	-0.7647007	-1.0296661	1.4010811	1.246907	1.4412845	1	1	1	1	1	1
GGB9345_SGB14311	-0.5318305	-0.1451503	0.3866802	1.29309	1.1504188	1.3750882	1	1	1	1	1	1
Oscillibacter_sp_MSJ_	-0.3190551	0.778435	1.0974901	1.3527809	1.2037507	1.4115298	1	1	1	1	1	1
Alistipes_finegoldii	-1.7918345	-1.2198264	0.5720082	1.3414085	1.1935896	1.4045586	1	1	1	1	1	1
Campylobacter_homi	0.2059915	1.2581891	1.0521975	1.4019695	1.2477008	1.4418339	1	1	1	1	1	1
Vescimonas_coproco	-0.87444	-0.2259783	0.6484617	1.453054	1.2933458	1.4735627	1	1	1	1	1	1
GGB9453_SGB14844	1.3132355	1.1640694	-0.1491661	1.1754544	1.0453278	1.3043397	1	1	1	1	1	1
Oscillospiraceae_bac	-1.0765652	0.0249977	1.1015629	1.37279	1.2216287	1.4238274	1	1	1	1	1	1
Clostridiales_bacteriu	-2.3859415	-1.4636334	0.922308	1.3830464	1.2307928	1.4301468	1	1	1	1	1	1
Anaerotignum_faecico	-1.4517977	-1.3345809	0.1172168	1.3232127	1.1773321	1.3934324	1	1	1	1	1	1
GGB9770_SGB15390	0.9131541	1.1420167	0.2288625	1.2961433	1.1531467	1.3769433	1	1	1	1	1	1
Bacteroides_cellulosi	-0.1778216	-0.0970275	0.0807941	1.4951349	1.3309464	1.4998904	1	1	1	1	1	1
Eubacterium_siraeum	0.2283447	1.3956699	1.1673252	1.4330884	1.2755061	1.4611313	1	1	1	1	1	1
Bifidobacterium_bifid	-0.266313	-1.8570808	-1.5907679	1.2887876	1.1465749	1.3724758	1	1	1	1	1	1

species	lfc_IBD	lfc_PD	lfc_PDvsIBD	se_IBD	se_PD	se_PDvsIBD	pval_IBD	pval_PD	pval_PDvsIBD	adj_pval_IBD	adj_pval_PD	adj_pval_PDvsIBD
Bifidobacterium_pseu	2.1894015	0.5555704	-1.6338311	1.5295875	1.361731	1.5215704	1	1	1	1	1	1
Bacteroides_nordii	0.933592	0.205929	-0.727663	1.1868859	1.0555392	1.3111543	1	1	1	1	1	1
GGB3433_SGB4574	1.691892	1.2250267	-0.4668653	1.3024068	1.1587429	1.380752	1	1	1	1	1	1
GGB38744_SGB1484	0.4119731	-0.1558384	-0.5678115	1.1637786	1.0348985	1.2973922	1	1	1	1	1	1
Blautia_stercoris	-0.5186057	-1.0410153	-0.5224096	1.1878254	1.0563784	1.3117149	1	1	1	1	1	1
Porphyromonas_some	-1.8881324	-0.8935553	0.9945771	1.4311974	1.2738164	1.4599559	1	1	1	1	1	1
Porphyromonas_asac	-0.6797281	0.2309028	0.910631	1.3942237	1.2407798	1.4370457	1	1	1	1	1	1
Porphyromonas_SGB1	-0.0579251	0.2884493	0.3463744	1.2771093	1.1361411	1.3653943	1	1	1	1	1	1
Peptoniphilus_lacrima	-1.0993863	-0.2971891	0.8021972	1.4234822	1.2669227	1.455164	1	1	1	1	1	1
Lawsonella_cleveland	-0.4421266	-0.3184493	0.1236773	1.2073262	1.0737984	1.3233708	1	1	1	1	1	1
Schaalia_turicensis	-0.158356	0.1719691	0.3303251	1.2700402	1.1298254	1.3611146	1	1	1	1	1	1
Prevotella_timonensis	-1.5232357	-0.2300469	1.2931889	1.4617492	1.3011152	1.4789888	1	1	1	1	1	1
Peptoniphilus_harei	-1.4536263	-0.2753903	1.178236	1.3873757	1.2346611	1.4328175	1	1	1	1	1	1
Prevotella_bergensis	-0.0358918	0.109077	0.1449688	1.1117809	0.9884573	1.2665992	1	1	1	1	1	1
GGB9524_SGB14924	-0.6977854	-0.6986382	-0.0008528	1.165217	1.0361834	1.2982474	1	1	1	1	1	1
Arcanobacterium_urir	0.3169224	0.5609769	0.2440545	1.1549965	1.0270543	1.2921749	1	1	1	1	1	1
Urinicoccus_timonens	-0.6633172	-0.3962316	0.2670856	1.1908358	1.0590675	1.3135118	1	1	1	1	1	1
Bacteroides_fragilis	1.9976236	0.9438903	-1.0537332	1.5422437	1.3730398	1.5295622	1	1	1	1	1	1
Anaerococcus_SGB66	-0.7823606	-0.7098413	0.0725193	1.2823526	1.1408256	1.368572	1	1	1	1	1	1
Bacteroides_salyersia	1.6705877	1.1597488	-0.5108389	1.1556827	1.0276672	1.2925823	1	1	1	1	1	1
Sellimonas_intestinali	0.9587484	0.9163203	-0.0424281	1.2606467	1.1214331	1.3554357	1	1	1	1	1	1
Ezakiella_coagulans	-0.0574598	0.1489471	0.2064068	1.3159956	1.1708839	1.3890287	1	1	1	1	1	1
Fenollaria_massiliens	-0.6977621	-0.3064716	0.3912905	1.2953857	1.1524698	1.3764829	1	1	1	1	1	1
Mediterraneibacter_gl	1.6230495	0.6241313	-0.9989183	1.1860662	1.0548069	1.3106652	1	1	1	1	1	1
Clostridium_SGB4750	0.3778116	-0.2658141	-0.6436257	1.1172574	0.9933482	1.2698318	1	1	1	1	1	1
GGB1455_SGB2018	-0.1071226	0.0681763	0.1752988	1.1385329	1.0123494	1.2824127	1	1	1	1	1	1
Campylobacter_ureol	0.0243298	0.1960064	0.1716766	1.1667635	1.0375647	1.2991671	1	1	1	1	1	1
Peptoniphilus_gorbac	-0.9638854	-0.9240043	0.0398811	1.2504776	1.1123481	1.349298	1	1	1	1	1	1
Anaerococcus_medite	0.5088428	0.3608122	-0.1480305	1.024841	0.910836	1.2155311	1	1	1	1	1	1
Butyricimonas_virosa	0.2408564	-0.3898577	-0.6307141	1.0963789	0.9747032	1.2575202	1	1	1	1	1	1
Frisingicoccus_SGB46	0.270521	0.6997013	0.4291803	1.2535855	1.1151247	1.3511727	1	1	1	1	1	1
Anaerococcus_murdo	-0.5425099	0.1840411	0.726551	1.2538202	1.1153343	1.3513143	1	1	1	1	1	1
GGB10524_SGB1704	-0.811551	-0.8297736	-0.0182226	1.1854291	1.0542378	1.3102851	1	1	1	1	1	1
Streptococcus_anginc	-1.7218956	-0.2772119	1.4446838	1.5296536	1.3617901	1.5216121	1	1	1	1	1	1
Erysipelatoclostridium	3.4190497	1.5357169	-1.8833328	1.2630683	1.1235966	1.3568988	1	1	1	1	1	1
Clostridium_sp_AT4	0.5964438	-0.8951363	-1.4915801	1.2145152	1.0802205	1.3276773	1	1	1	1	1	1

Supplementary Table 2: UFPF all genus-level ANCOM-BC2 output

Differential abundances of genera in PD (N = 54) vs. IBD (N = 26) vs. Healthy Control (N=16) were tested using ANCOM-BC2, while adjusting for covariates (diagnosis and total sequence count per sample). Shown are all 140 genera that were detected by MetaPhlAn, however, only genera that were present in at least 10% of samples were included in ANCOM-BC2 analysis. LFC: indicates the log fold change compared to healthy control or in the case of lfc_PDvsIBD it is the log fold change of PD compared to IBD. SE: standard error; Pval: the uncorrected, two-sided P-value reported by ANCOM-BC2; Adj. Pval aka q-value from the false discovery rate, i.e., multiple-testing corrected significance q-value, calculated using the Benjamini-Hochberg method, alpha =0.05.

genus	lfc_IBD	lfc_PD	lfc_PDvsIBD	se_IBD	se_PD	se_PDvsIBD	pval_IBD	pval_PD	pval_PDvsIB	adj_pval_IBD	adj_pval_PD	adj_pval_PDvsIBD
Klebsiella	5.9838547	-0.4905578	-6.4744125	1.5249969	1.3558347	1.58999317	8.7146E-05	0.71749224	4.6616E-05	0.01821352	1	0.0097894
Faecalimonas	6.15170955	0.51459602	-5.6371135	1.37905092	1.22542294	1.49889964	8.1643E-06	0.67453411	0.00016935	0.00171451	1	0.03539361
Porphyromonas	-3.1549307	1.25713601	4.4120667	1.89537987	1.6868254	1.82816214	1	1	1	1	1	1
Corynebacterium	-1.0060846	-0.4542492	0.55183539	1.90234083	1.69304595	1.83272711	1	1	1	1	1	1
GGB2945	0.2272441	0.91710208	0.68985797	1.56819781	1.39444015	1.61726409	1	1	1	1	1	1
Fenollaria	-2.6694431	1.54060629	4.2100494	1.77587058	1.58002673	1.75027249	1	1	1	1	1	1
Ezakiella	-2.7539247	0.21872034	2.97264504	1.70276115	1.51469232	1.70309598	1	1	1	1	1	1
Peptoniphilus	-3.3463948	1.43278862	4.77918339	1.71191541	1.52287305	1.70898268	1	1	1	1	1	1
Varibaculum	-3.4622566	1.28542656	4.74768315	1.73552456	1.54397144	1.72419188	1	1	1	1	1	1
Eubacteriales_uncl	-3.2412519	-1.4972904	1.7439615	1.61009288	1.43187926	1.64384438	1	1	1	1	1	1
Prevotella	-4.8257922	1.5481513	6.37394352	1.73829732	1.54644933	1.72598067	1	1	1	1	1	1
Collinsella	1.28785693	-0.0136551	-1.3015121	1.67982942	1.4941993	1.68837573	1	1	1	1	1	1
Ruminococcus	-1.9663892	-0.7811723	1.18521685	1.92753388	1.71555924	1.84927361	1	1	1	1	1	1
GGB9715	-2.8726847	-2.3041	0.56858475	1.66744882	1.48313534	1.68044408	1	1	1	1	1	1
Mobiluncus	0.46175743	4.58329212	4.12153469	1.6454339	1.46346169	1.66636758	1	1	1	1	1	1
Alterileibacterium	0.64527861	1.98113775	1.33585914	1.52932788	1.35970494	1.59272079	1	1	1	1	1	1
Peptococcus	-0.7205883	1.03037066	1.75095899	1.54800006	1.37639083	1.6044966	1	1	1	1	1	1
Gemmiger	-4.0184514	-1.3357632	2.68268827	1.70777015	1.51916862	1.70631631	1	1	1	1	1	1
GGB1456	-0.1807713	1.54658217	1.72735345	1.58786636	1.4120167	1.62972647	1	1	1	1	1	1
Oscillibacter	-2.7825647	-0.3132096	2.46935509	1.65901303	1.47559669	1.67504602	1	1	1	1	1	1
Alistipes	-3.400008	-1.3208933	2.0791147	1.66806618	1.48368705	1.68083933	1	1	1	1	1	1
Blautia	1.84600767	-0.9791049	-2.8251126	1.51353274	1.34559017	1.58277997	1	1	1	1	1	1
Evtepia	-0.6216812	0.02139856	0.64307978	1.73647473	1.54482056	1.7248048	1	1	1	1	1	1
Faecalibacterium	-4.7451695	-1.7161248	3.02904465	1.7796123	1.58337052	1.75269686	1	1	1	1	1	1
Phascolarctobacte	0.53517487	0.82579112	0.29061624	1.70202923	1.51403823	1.70262557	1	1	1	1	1	1
GGB9512	-0.8420239	-1.0902083	-0.2481844	1.60352706	1.42601176	1.63967006	1	1	1	1	1	1
Bacteroides	1.15020202	0.67616177	-0.4740403	1.43270172	1.27336091	1.53220256	1	1	1	1	1	1
Dorea	-2.8519699	-2.9035733	-0.0516033	1.71979334	1.52991319	1.71405336	1	1	1	1	1	1
Bifidobacterium	1.15493721	-1.5290491	-2.6839863	1.87766616	1.67099579	1.81655921	1	1	1	1	1	1
Wujia	-1.1470647	-1.6302939	-0.4832292	1.30031551	1.15507989	1.45038626	1	1	1	1	1	1
Anaerobutyricum	-2.2145497	-1.0021877	1.21236198	1.72069705	1.53072079	1.71463532	1	1	1	1	1	1
Lagierella	0.81003829	0.45434368	-0.3556946	1.18128604	1.04877322	1.37767373	1	1	1	1	1	1

genus	lfc_IBD	lfc_PD	lfc_PDvsIBD	se_IBD	se_PD	se_PDvsIBD	pval_IBD	pval_PD	pval_PDvsIBD	adj_pval_IBD	adj_pval_PD	adj_pval_PDvsIBD
Clostridiaceae_unc	-2.3685145	-1.798633	0.5698815	1.74593604	1.55327569	1.73091138	1	1	1	1	1	1
Mediterraneibacter	-0.1849174	-1.6989094	-1.513992	1.56643366	1.39286365	1.61614771	1	1	1	1	1	1
Acidaminococcus	1.57368466	0.10529875	-1.4683859	1.53710538	1.36665508	1.59762256	1	1	1	1	1	1
Barnesiella	-0.2703041	-0.7795007	-0.5091966	1.56111688	1.3881124	1.61278457	1	1	1	1	1	1
Adlercreutzia	1.04311089	0.95043674	-0.0926742	1.58579465	1.41016534	1.62841244	1	1	1	1	1	1
Actinomyces	0.9515865	0.36332456	-0.5882619	1.32734001	1.17922242	1.4669922	1	1	1	1	1	1
Candidatus_Cibion	-2.8651803	-1.1193048	1.7458755	1.76788258	1.57288825	1.74510001	1	1	1	1	1	1
Lachnospiraceae_u	-2.7765107	-2.4043055	0.37220525	1.82255184	1.62174335	1.78058584	1	1	1	1	1	1
Coprococcus	-3.5957394	-2.195523	1.40021641	1.64711479	1.46496381	1.66744111	1	1	1	1	1	1
Phocaeicola	-1.2863497	-1.0203999	0.26594987	1.50579156	1.33867258	1.57791487	1	1	1	1	1	1
Faecalicatena	-1.0825281	-1.9775573	-0.8950291	1.45511118	1.29338511	1.54617544	1	1	1	1	1	1
Mogibacterium	-0.1631114	-0.6613897	-0.4982783	1.22360546	1.0865623	1.4034651	1	1	1	1	1	1
Fusicatenibacter	-3.1140203	-3.684153	-0.5701327	1.84744089	1.64398529	1.79680679	1	1	1	1	1	1
Agathobaculum	-2.6966213	-3.3737508	-0.6771295	1.61159489	1.43322152	1.64479975	1	1	1	1	1	1
Brotolimicola	-1.8087315	-2.1880069	-0.3792754	1.50149909	1.33483681	1.57521913	1	1	1	1	1	1
Eubacteriales_Fam	-1.0732286	2.39804152	3.47127008	1.69313675	1.50609143	1.69691336	1	1	1	1	1	1
Olegusella	-0.245615	-0.0034093	0.24220567	1.35345392	1.20255299	1.48308282	1	1	1	1	1	1
GGB4277	-2.7455098	0.5225084	3.26801825	1.67189428	1.48710804	1.6832908	1	1	1	1	1	1
Oscillospiraceae_u	-3.9047179	-1.3054793	2.59923858	1.65422049	1.47131382	1.67198157	1	1	1	1	1	1
Oliverpabstia	-1.4883031	-2.2654191	-0.7771161	1.67197931	1.48718402	1.68334527	1	1	1	1	1	1
GGB9760	-0.5168601	-0.976617	-0.4597569	1.42602338	1.2673935	1.52804564	1	1	1	1	1	1
GGB9635	-0.3538152	-0.5410735	-0.1872583	1.28265855	1.13930693	1.43955986	1	1	1	1	1	1
Parabacteroides	0.26001098	0.31590421	0.05589323	1.54202164	1.37104836	1.60072338	1	1	1	1	1	1
Lawsonella	-2.6263179	0.46798939	3.09430724	1.64970473	1.46727831	1.66909563	1	1	1	1	1	1
Roseburia	-1.6513235	-2.3201741	-0.6688506	1.79719578	1.59908398	1.76410235	1	1	1	1	1	1
Negativibacillus	-0.2998935	-1.312795	-1.0129014	1.39776017	1.24213958	1.51048949	1	1	1	1	1	1
GGB3433	1.85027101	1.40611173	-0.4441593	1.67825507	1.49279238	1.68736651	1	1	1	1	1	1
Escherichia	5.8697726	-0.3331782	-6.2029508	1.90195994	1.69270558	1.83247724	1	1	1	1	1	1
Dysosmobacter	-1.0506818	0.88662371	1.93730554	1.6263642	1.44642006	1.65420284	1	1	1	1	1	1
Paraprevotella	0.68635283	-0.5993174	-1.2856702	1.3580122	1.20662556	1.48589616	1	1	1	1	1	1
Enterocloster	0.33956802	0.72236039	0.38279237	1.68411433	1.49802852	1.69112343	1	1	1	1	1	1
GGB3167	3.02272409	1.73981453	-1.2829096	1.38093632	1.22710752	1.50006647	1	1	1	1	1	1
Odoribacter	-1.962183	-1.1375633	0.82461973	1.48849312	1.32321467	1.56705967	1	1	1	1	1	1
Streptococcus	0.31651994	-2.0269504	-2.3434704	1.68451033	1.4983824	1.69137743	1	1	1	1	1	1
Monoglobus	0.01799779	-1.4088604	-1.4268582	1.38586701	1.23151303	1.50311912	1	1	1	1	1	1
GGB51647	-0.982757	-2.583121	-1.600364	1.36186468	1.21006757	1.48827499	1	1	1	1	1	1
Eisenbergiella	1.70069518	3.10890022	1.40820504	1.64067768	1.45921129	1.66333105	1	1	1	1	1	1
Intestinimonas	2.16495085	1.52365385	-0.641297	1.37785075	1.22435061	1.49815702	1	1	1	1	1	1
Anaerostipes	-1.8128886	-2.1903672	-0.3774787	1.84524508	1.64202303	1.7953741	1	1	1	1	1	1
Ruthenibacterium	-0.5292326	1.27536773	1.80460037	1.65175283	1.46910859	1.67040435	1	1	1	1	1	1

genus	lfc_IBD	lfc_PD	lfc_PDvsIBD	se_IBD	se_PD	se_PDvsIBD	pval_IBD	pval_PD	pval_PDvsIBD	adj_pval_IBD	adj_pval_PD	adj_pval_PDvsIBD
Lachnoclostridium	3.20754747	2.86849592	-0.3390516	1.60192873	1.42458342	1.63865438	1	1	1	1	1	1
Clostridium	-1.9407909	-2.2131014	-0.2723105	1.70178974	1.51382421	1.70247166	1	1	1	1	1	1
Eggerthella	2.27750331	1.67839028	-0.599113	1.69512887	1.5078717	1.69819254	1	1	1	1	1	1
GGB9719	2.44329154	0.84741792	-1.5958736	1.30942012	1.16321337	1.45597585	1	1	1	1	1	1
Bilophila	-0.2422666	1.4976644	1.73993101	1.50151858	1.33485422	1.57523137	1	1	1	1	1	1
Clostridia_unclassi	0.76146007	0.45220994	-0.3092501	1.32301559	1.17535906	1.4643319	1	1	1	1	1	1
Brevibacterium	-0.1553973	-0.873257	-0.7178597	1.31046463	1.16414647	1.45661742	1	1	1	1	1	1
Parasutterella	0.76504177	-1.2495137	-2.0145554	1.5637598	1.39047421	1.61445609	1	1	1	1	1	1
Anaerotruncus	2.05612464	1.17040748	-0.8857172	1.24837849	1.10868732	1.41858802	1	1	1	1	1	1
Akkermansia	-0.6653236	0.4635672	1.12889079	1.89470823	1.6862252	1.82772184	1	1	1	1	1	1
Eubacterium	-0.9100655	-2.1713944	-1.2613289	1.55648966	1.38397738	1.60985935	1	1	1	1	1	1
Lachnospira	-3.0301303	-2.7370151	0.29311523	1.58378985	1.40837378	1.62714114	1	1	1	1	1	1
Neglectibacter	1.3576564	-0.441987	-1.7996434	1.38159504	1.22769608	1.50047419	1	1	1	1	1	1
Lawsonibacter	-0.0107667	-0.9295204	-0.9187538	1.4482166	1.28722434	1.54187251	1	1	1	1	1	1
Desulfovibrio	-1.2791667	-1.7419428	-0.4627761	1.4476158	1.28668749	1.54149771	1	1	1	1	1	1
Schaalia	1.13543344	-0.5680423	-1.7034757	1.44407838	1.28352658	1.53929153	1	1	1	1	1	1
GGB9480	1.22037132	1.13098324	-0.0893881	1.611012	1.43270063	1.64442898	1	1	1	1	1	1
Flavonifractor	-0.148926	1.12730381	1.27622982	1.59533063	1.41868707	1.63446355	1	1	1	1	1	1
Dialister	-1.0458951	-0.9338951	0.11199992	1.69792287	1.51036856	1.69998709	1	1	1	1	1	1
Finegoldia	-3.3141733	0.55428857	3.86846185	1.66774599	1.48340091	1.68063434	1	1	1	1	1	1
Anaerococcus	-1.6895266	0.78289727	2.4724239	1.69459434	1.50739402	1.69784928	1	1	1	1	1	1
GGB9342	-0.3370524	-0.2907032	0.04634918	1.39831415	1.24263456	1.51083304	1	1	1	1	1	1
GGB9708	-1.5462847	-0.5389483	1.00733639	1.49919881	1.33278127	1.57377509	1	1	1	1	1	1
GGB3653	-0.2523845	-0.1017524	0.15063216	1.44020574	1.28006615	1.53687735	1	1	1	1	1	1
GGB36331	0.25996776	0.01747634	-0.2424914	1.2491108	1.10934139	1.41903545	1	1	1	1	1	1
GGB9365	-0.7456656	-1.6665706	-0.920905	1.42446077	1.26599724	1.52707348	1	1	1	1	1	1
GGB9730	-0.1491105	-1.2870815	-1.1379711	1.31587616	1.1689809	1.45994243	1	1	1	1	1	1
Sutterella	-3.0224584	-1.8745865	1.14787191	1.55104893	1.37911538	1.60642189	1	1	1	1	1	1
GGB32900	-1.261599	-1.4860491	-0.2244501	1.35908876	1.20758742	1.48656081	1	1	1	1	1	1
Lentihominibacter	-0.0375453	0.13450642	0.1720517	1.35630427	1.20509962	1.48484187	1	1	1	1	1	1
Butyricimonas	-1.3038374	-1.2062186	0.09761881	1.27741688	1.13462473	1.43634926	1	1	1	1	1	1
Methanobrevibacte	-1.7899134	-1.0689402	0.7209732	1.40643295	1.24988883	1.51587054	1	1	1	1	1	1
GGB9699	-0.979856	-0.6272792	0.35257679	1.44747658	1.28656308	1.54141086	1	1	1	1	1	1
Lacrimispora	-0.8444611	-1.8722221	-1.0277611	1.38898512	1.23429905	1.50505049	1	1	1	1	1	1
GGB2980	0.76789645	0.09200299	-0.6758935	1.07446136	0.95344577	1.31245871	1	1	1	1	1	1
GGB1215	0.40909143	1.60487237	1.19578094	1.52934175	1.35971734	1.59272953	1	1	1	1	1	1
Levyella	-2.8851929	0.16574244	3.05093535	1.86885592	1.66312263	1.81079565	1	1	1	1	1	1
Megasphaera	1.55997138	-0.6359337	-2.1959051	1.35551888	1.20439791	1.48435712	1	1	1	1	1	1
Peptostreptococcu	0.27991919	0.11187657	-0.1680426	1.34384587	1.19396886	1.47715738	1	1	1	1	1	1
GGB3109	0.66094834	2.25725825	1.5963099	1.44786321	1.28690856	1.54165205	1	1	1	1	1	1

genus	lfc_IBD	lfc_PD	lfc_PDvsIBD	se_IBD	se_PD	se_PDvsIBD	pval_IBD	pval_PD	pval_PDvsIBD	adj_pval_IBD	adj_pval_PD	adj_pval_PDvsIBD
Erysipelatoclostridi	3.13652563	3.15685425	0.02032862	1.62692948	1.44692523	1.65456306	1	1	1	1	1	1
Hungatella	0.38745627	1.19492739	0.80747112	1.52768841	1.35823988	1.5916881	1	1	1	1	1	1
Fusobacterium	1.45225435	0.67704484	-0.7752095	1.30257954	1.15710241	1.45177576	1	1	1	1	1	1
Enterococcus	4.07566553	0.35274478	-3.7229207	1.38425257	1.23007054	1.50211941	1	1	1	1	1	1
GGB9345	-0.3962575	-0.4194026	-0.0231451	1.36727781	1.21490398	1.4916192	1	1	1	1	1	1
GGB9770	0.38325123	0.41359529	0.03034405	1.44390538	1.28337199	1.53918366	1	1	1	1	1	1
Campylobacter	-1.0254972	0.87162043	1.89711759	1.57183242	1.39768817	1.61956487	1	1	1	1	1	1
Pseudoflavonifracto	0.69530948	0.17047411	-0.5248354	1.27066751	1.12859586	1.4322173	1	1	1	1	1	1
Vescimonas	-0.887881	-0.2439125	0.64396855	1.56493826	1.39152732	1.61520158	1	1	1	1	1	1
GGB9453	1.44753667	0.90201759	-0.5455191	1.25890513	1.11808949	1.4250219	1	1	1	1	1	1
Anaerotignum	-1.3164156	-1.1537337	0.1626819	1.44746192	1.28654998	1.54140172	1	1	1	1	1	1
Gordonibacter	1.70467062	1.78474141	0.08007079	1.22676624	1.08938508	1.40539334	1	1	1	1	1	1
Tyzzerella	1.61161894	1.86645862	0.25483968	1.5451652	1.37385752	1.60270707	1	1	1	1	1	1
Veillonella	1.82479078	0.22563926	-1.5991515	1.34713799	1.19691012	1.47918699	1	1	1	1	1	1
GGB38744	0.54754611	-0.4300907	-0.9776368	1.24207525	1.1030576	1.41473784	1	1	1	1	1	1
GGB33512	0.6742375	0.19601851	-0.478219	1.39563625	1.24024185	1.50917252	1	1	1	1	1	1
Slackia	-0.6836856	-1.477064	-0.7933784	1.29489	1.1502332	1.44705772	1	1	1	1	1	1
Firmicutes_unclass	2.07797301	0.21721241	-1.8607606	1.43108004	1.27191186	1.53119284	1	1	1	1	1	1
GGB9524	-0.5555752	-0.2053033	0.35027191	1.41617577	1.2585943	1.52192202	1	1	1	1	1	1
Arcanobacterium	0.45249538	0.28672461	-0.1657708	1.23359345	1.09548235	1.4095595	1	1	1	1	1	1
Urinicoccus	-0.5667418	-0.5748916	-0.0081498	1.28551723	1.14186053	1.44131148	1	1	1	1	1	1
GGB4260	-0.3673746	-0.8485818	-0.4812072	1.23765734	1.09911185	1.41204024	1	1	1	1	1	1
Sellimonas	1.09432135	0.64206796	-0.4522534	1.33581164	1.18679096	1.47220726	1	1	1	1	1	1
Frisingicoccus	-0.4645496	0.07473039	0.53927994	1.42678112	1.26807057	1.52851713	1	1	1	1	1	1
GGB3550	2.05293539	0.95895573	-1.0939797	1.31392384	1.16723678	1.45874266	1	1	1	1	1	1
GGB1455	0.02845044	-0.206076	-0.2345265	1.21770007	1.08128853	1.39986338	1	1	1	1	1	1
GGB10524	-0.4350321	-0.8084362	-0.3734041	1.31529809	1.16846448	1.45958717	1	1	1	1	1	1
Facklamia	-0.2963314	-1.0040963	-0.7077649	1.19714108	1.06292983	1.38733206	1	1	1	1	1	1

Supplementary Table 3: UFPF all MetaCyc pathways ANCOM-BC2 output

Differential abundances of MetaCyc pathways in PD (N = 54) vs. IBD (N = 26) vs. Healthy Control (N=16) were tested using ANCOM-BC2, while adjusting for covariates (diagnosis and total sequence count per sample). Shown are all 350 pathways that were detected by HUMAnN, however, only pathways that were present in at least 25% of samples were included in ANCOM-BC2 analysis. LFC: indicates the log fold change compared to healthy control or in the case of lfc_PDvsIBD it is the log fold change of PD compared to IBD. SE: standard error; Pval: the uncorrected, two-sided P-value reported by ANCOM-BC2; Adj. Pval aka q-value from the false discovery rate, i.e., multiple-testing corrected significance q-value, calculated using the Benjamini-Hochberg method, alpha =0.05.

pathway	lfc_IBD	lfc_PD	lfc_PDvsIBD	se_IBD	se_PD	se_PDvsIBI	pval_IBD	pval_PD	pval_PDvsI	adj_pval_IB	adj_pval_PI	adj_pval_PDvsIBD
1CMET2-PWY: folate	-0.01972893	0.075067717	0.094796644	0.272119	0.239435	0.337619	1	1	1	1	1	1
ALLANTOINDEG-PW	0.475023084	0.094901518	-0.380121566	0.541985	0.481057	0.50562	1	1	1	1	1	1
ANAEROFRUCAT-PV	0.094818502	0.074803484	-0.020015018	0.489512	0.434032	0.471262	1	1	1	1	1	1
ANAGLYCOLYSIS-PV	-0.14071797	0.180405671	0.32112364	0.485581	0.430508	0.468716	1	1	1	1	1	1
ARG+POLYAMINE-S	1.450901815	0.01103001	-1.439871805	0.694942	0.618102	0.609086	1	1	1	1	1	1
ARGDEG-PWY: supe	2.658537374	-0.150175512	-2.808712886	0.514034	0.456008	0.487231	2.32E-07	0.741909	8.18E-09	1.83E-06	1	7.28E-08
ARGININE-SYN4-PW	0.82883156	-0.057208379	-0.886039938	0.518022	0.459582	0.489843	1	1	1	1	1	1
ARGSYN-PWY: L-arg	-0.03225496	0.083262149	0.11551711	0.498817	0.44237	0.477302	1	1	1	1	1	1
ARGSYNBSUB-PWY:	-0.02156424	0.096346998	0.117911238	0.50084	0.444184	0.478619	1	1	1	1	1	1
ARO-PWY: chorisma	-0.1833521	0.143899791	0.327251888	0.487012	0.431791	0.469642	1	1	1	1	1	1
ASPASN-PWY: super	0.445621285	0.143479939	-0.302141345	0.495523	0.439418	0.475161	1	1	1	1	1	1
AST-PWY: L-arginine	2.92170807	-0.245318323	-3.167026393	0.662172	0.588745	0.586576	1.02E-05	0.676912	6.69E-08	8.08E-05	1	5.96E-07
BIOTIN-BIOSYNTHES	0.705940044	-0.420750111	-1.126690155	0.581393	0.51637	0.531853	1	1	1	1	1	1
BRANCHED-CHAIN-	0.127429173	0.136254892	0.008825718	0.49438	0.438394	0.474419	1	1	1	1	1	1
CALVIN-PWY: Calvir	0.085498487	0.207558384	0.122059897	0.488808	0.433401	0.470806	1	1	1	1	1	1
CENTFERM-PWY: py	-2.78821581	-0.829438426	1.958777389	0.577726	0.513084	0.529397	1.39E-06	0.10597	0.000216	1.24E-05	0.731015	0.001703
CITRULBIO-PWY: L-(	0.966535995	0.102918778	-0.863617218	0.422608	0.374069	0.42857	1	1	1	1	1	1
COA-PWY-1: superp	-0.45847585	0.202341508	0.660817359	0.484768	0.42978	0.468191	1	1	1	1	1	1
COA-PWY: coenzym	-0.51632485	0.213318002	0.729642855	0.4839	0.429002	0.46763	1	1	1	1	1	1
COBALSYN-PWY: su	0.113675977	-0.432704259	-0.546380236	0.337773	0.298066	0.376562	1	1	1	1	1	1
COLANSYN-PWY: co	0.135297987	0.140725107	0.005427119	0.483117	0.4283	0.467123	1	1	1	1	1	1
COMPLETE-ARO-PW	-0.09082587	0.045035749	0.135861621	0.246763	0.217043	0.321891	1	1	1	1	1	1
DAPLYSINESYN-PWY	0.647652041	0.006167282	-0.641484759	0.359216	0.317268	0.389474	1	1	1	1	1	1
DARABCATK12-PWY	1.995094809	-0.013987742	-2.009082552	0.483424	0.428576	0.467322	3.68E-05	0.973963	1.71E-05	0.00029	1	0.000153
DTDPRHAMSYN-PW	-0.59567949	0.128661757	0.724341252	0.499769	0.443224	0.477922	1	1	1	1	1	1
ECASYN-PWY: enter	1.833269818	-0.459727066	-2.292996884	0.511509	0.453746	0.48558	0.000338	0.310973	2.33E-06	0.002672	1	2.08E-05
FAO-PWY: fatty acid	2.787142021	-0.338275915	-3.125417936	0.80762	0.71903	0.68756	0.000558	0.638025	5.48E-06	0.00441	1	4.87E-05
FASYN-ELONG-PWY	0.656561937	-0.07480171	-0.731363647	0.496358	0.440167	0.475704	1	1	1	1	1	1
FERMENTATION-PW	0.229579453	-0.265020415	-0.494599868	0.473293	0.419496	0.46079	1	1	1	1	1	1
FOLSYN-PWY: super	0.689284605	-1.143247581	-1.832532186	0.670135	0.595879	0.592031	0.303679	0.055036	0.001966	1	0.434694	0.017494
FUC-RHAMCAT-PWY	1.127677157	-0.402289112	-1.529966269	0.638241	0.567306	0.570244	1	1	1	1	1	1
FUCCAT-PWY: fucos	1.22971142	-0.018211643	-1.247923063	0.564798	0.5015	0.520765	1	1	1	1	1	1

pathway	lfc_IBD	lfc_PD	lfc_PDvsIBD	se_IBD	se_PD	se_PDvsIBD	pval_IBD	pval_PD	pval_PDvsI	adj_pval_IB	adj_pval_PI	adj_pval_PDvsIBD
GALACT-GLUCURO	0.718485369	-0.595275012	-1.31376038	0.622161	0.552899	0.559325	1	1	1	1	1	1
GALACTARDEG-PWY	2.381426093	-0.310140016	-2.691566109	0.702929	0.625257	0.614595	0.000704	0.61988	1.19E-05	0.005564	1	0.000106
GALACTITOLCAT-PW	2.094925658	0.004209645	-2.090716013	0.699726	0.622388	0.612385	0.002754	0.994603	0.00064	0.021753	1	0.005695
GALACTUROCAT-PW	0.469595266	0.037045398	-0.432549868	0.492096	0.436347	0.472937	1	1	1	1	1	1
GLCMANNANAUT-P	0.350598418	0.04417435	-0.306424068	0.41584	0.368003	0.42433	1	1	1	1	1	1
GLUCARDEG-PWY:	2.290936666	-0.442459468	-2.733396133	0.684003	0.608303	0.601554	0.00081	0.467001	5.52E-06	0.006399	1	4.91E-05
GLUCARGALACTSU	2.381426093	-0.310140016	-2.691566109	0.702929	0.625257	0.614595	0.000704	0.61988	1.19E-05	0.005564	1	0.000106
GLUCONEO-PWY: g	0.150655652	-0.024335068	-0.17499072	0.240153	0.21131	0.317269	1	1	1	1	1	1
GLUCOSE1PMETAB-	1.417104425	-0.089579614	-1.506684038	0.76664	0.682326	0.658847	1	1	1	1	1	1
GLUCUROCAT-PWY	0.093398439	-0.409793699	-0.503192138	0.452264	0.400649	0.447326	1	1	1	1	1	1
GLUDEG-I-PWY: GA	0.534973902	-0.903594393	-1.438568295	0.535028	0.474822	0.501026	1	1	1	1	1	1
GLUTORN-PWY: L-o	0.066664691	0.008439907	-0.058224784	0.507731	0.45036	0.483112	1	1	1	1	1	1
GLYCOCAT-PWY: gly	0.810707522	-0.240167386	-1.050874908	0.565815	0.502411	0.521443	1	1	1	1	1	1
GLYCOGENSYNTH-I	-0.14017013	0.018365846	0.158535975	0.499143	0.442663	0.477515	1	1	1	1	1	1
GLYCOL-GLYOXDEG	1.819100247	-0.61959053	-2.438690776	0.561534	0.498575	0.518591	0.001197	0.21397	2.57E-06	0.009457	1	2.29E-05
GLYCOLYSIS-E-D: s	0.475463497	-0.319961293	-0.79542479	0.58027	0.515363	0.5311	1	1	1	1	1	1
GLYCOLYSIS-TCA-G	1.722109413	-0.308283302	-2.030392715	0.572336	0.508254	0.525794	0.002622	0.544147	0.000113	0.020708	1	0.001002
GLYCOLYSIS: glycol	0.14534943	0.083534836	-0.061814595	0.493429	0.437542	0.473802	1	1	1	1	1	1
GLYOXYLATE-BYPAS	1.773510154	-0.006689276	-1.78019943	0.66955	0.595354	0.59163	0.008078	0.991035	0.002621	0.063799	1	0.023325
GOLPDLCAT-PWY: s	0.2503546	-0.692708647	-0.943063248	0.528108	0.468621	0.496467	1	1	1	1	1	1
HCAMHPDEG-PWY:	2.97847032	0.163515319	-2.814955	0.643734	0.572227	0.573985	3.71E-06	0.775068	9.38E-07	2.93E-05	1	8.35E-06
HEME-BIOSYNTHES	2.073519602	-0.396443878	-2.46996348	0.771223	0.686431	0.662049	0.007175	0.563572	0.000191	0.05667	1	0.001699
HEME-BIOSYNTHES	1.058151538	0.008130149	-1.050021389	0.668303	0.594237	0.590775	1	1	1	1	1	1
HEMESYN2-PWY: he	0.724913079	-0.139110201	-0.86402328	0.624242	0.554763	0.560735	1	1	1	1	1	1
HEXITOLDEGSUPER	2.089405501	-0.219607321	-2.309012823	0.773351	0.688337	0.663536	0.006897	0.749696	0.000502	0.054478	1	0.004464
HISDEG-PWY: L-hist	-1.16011229	0.341475718	1.50158801	0.43564	0.385748	0.436777	0.007745	0.376033	0.000586	0.061169	1	0.005217
HISTSYN-PWY: L-his	-0.02384954	0.001987468	0.025837007	0.292318	0.257427	0.349597	0.934975	0.99384	0.941086	1	1	1
HOMOSER-METSYN	2.092994872	-0.347311558	-2.440306431	0.731434	0.650791	0.634331	0.004216	0.593566	0.00012	0.033303	1	0.001064
HSERMETANA-PWY:	-1.12532219	0.178441909	1.303764099	0.334497	0.295133	0.374602	0.000768	0.545435	0.000501	0.006063	1	0.004455
ILEUSYN-PWY: L-iso	0.154951638	0.130715182	-0.024236455	0.495806	0.439673	0.475346	1	1	1	1	1	1
KDO-NAGLIPASYN-I	2.033566196	-0.193747958	-2.227314154	0.502692	0.445844	0.479825	5.22E-05	0.663878	3.45E-06	0.000413	1	3.07E-05
KETOGLUCONMET-I	2.139615724	0.22999885	-1.909616874	0.673311	0.598724	0.59421	0.001484	0.700869	0.00131	0.011723	1	0.01166
LACTOSECAT-PWY:	-1.72586795	-0.444979046	1.280888902	0.505359	0.448233	0.481563	0.000638	0.320837	0.007817	0.005673	1	0.061745
LIPASYN-PWY: phos	0.197018071	0.042967903	-0.154050168	0.743216	0.661344	0.642518	1	1	1	1	1	1
MET-SAM-PWY: supe	1.973103355	-0.393220583	-2.366323938	0.789832	0.703098	0.675074	0.012485	0.575978	0.000456	0.09861	1	0.004059
METH-ACETATE-PW	-1.18702593	-0.991601211	0.195424717	0.742113	0.660357	0.641751	1	1	1	1	1	1
METHGLYUT-PWY: s	1.69268785	-0.236071508	-1.928759357	0.514294	0.456241	0.487401	0.000997	0.604858	7.58E-05	0.007877	1	0.000675
METSYN-PWY: supe	2.033465699	-0.383519812	-2.416985511	0.782338	0.696386	0.669824	0.009344	0.58182	0.000308	0.073799	1	0.002742
NAD-BIOSYNTHESIS	1.617789507	-0.181155142	-1.798944649	0.556164	0.493763	0.51502	0.003628	0.713703	0.000478	0.028653	1	0.004251

pathway	lfc_IBD	lfc_PD	lfc_PDvsIBD	se_IBD	se_PD	se_PDvsIBI	pval_IBD	pval_PD	pval_PDvsI	adj_pval_IB	adj_pval_PI	adj_pval_PDvsIBD
NAGLIPASYN-PWY: l	0.704516038	-0.059579214	-0.764095252	0.548602	0.486987	0.510001	1	1	1	1	1	1
NONMEVIPP-PWY: n	-0.34078121	0.208120398	0.548901608	0.484899	0.429897	0.468276	1	1	1	1	1	1
NONOXIPENT-PWY:	0.249098982	0.183242825	-0.065856157	0.49528	0.439201	0.475004	1	1	1	1	1	1
OANTIGEN-PWY: O-	-0.55238346	0.083684075	0.63606753	0.321555	0.283553	0.366892	1	1	1	1	1	1
ORNARGDEG-PWY:	2.658537374	-0.150175512	-2.808712886	0.514034	0.456008	0.487231	2.32E-07	0.741909	8.18E-09	1.83E-06	1	7.28E-08
ORNDEG-PWY: supe	2.104288476	-0.678951196	-2.783239672	0.779233	0.693605	0.667651	0.006924	0.327643	3.06E-05	0.054691	1	0.000273
P105-PWY: TCA cycl	1.14509961	-0.17941308	-1.32451269	0.671949	0.597504	0.593275	1	1	1	1	1	1
P108-PWY: pyruvate	0.91244004	-0.680003467	-1.592443507	0.599587	0.532672	0.544073	1	1	1	1	1	1
P122-PWY: heterola	1.597290422	-0.643705418	-2.24099584	0.611671	0.5435	0.552226	0.009018	0.236266	4.95E-05	0.071231	1	0.00044
P124-PWY: Bifidoba	-0.84278927	-0.771869825	0.070919442	0.693399	0.616719	0.608022	1	1	1	1	1	1
P161-PWY: acetylen	0.911088548	-0.476867214	-1.387955762	0.615213	0.546673	0.55462	1	1	1	1	1	1
P164-PWY: purine n	-1.54926612	0.069355569	1.618621684	0.576784	0.51224	0.528767	0.00723	0.892298	0.002205	0.057108	1	0.019622
P185-PWY: formalde	0.153393618	-0.55054557	-0.703939188	0.576049	0.511581	0.528276	1	1	1	1	1	1
P221-PWY: octane c	2.049919677	0.075563931	-1.974355746	0.554368	0.492153	0.513826	0.000218	0.877974	0.000122	0.001718	1	0.001084
P23-PWY: reductive	-1.06381326	0.15140645	1.21521971	0.568172	0.504523	0.523015	1	1	1	1	1	1
P4-PWY: superpathv	1.689356917	-0.587755488	-2.277112406	0.771498	0.686677	0.662241	0.028545	0.39203	0.000585	0.225459	1	0.005205
P41-PWY: pyruvate f	-0.47674917	0.093032305	0.569781472	0.500227	0.443634	0.47822	1	1	1	1	1	1
P42-PWY: incomplet	-1.67106534	0.154102629	1.825167968	0.634709	0.564141	0.567842	0.008468	0.784728	0.001308	0.066885	1	0.011639
P441-PWY: superpa	0.422045946	-0.49919039	-0.921236336	0.57742	0.51281	0.529193	1	1	1	1	1	1
P461-PWY: hexitol fe	1.150872968	-0.179140103	-1.330013071	0.622584	0.553277	0.559611	1	1	1	1	1	1
PANTO-PWY: phosp	-0.16393845	0.247178331	0.411116777	0.482079	0.427369	0.466453	1	1	1	1	1	1
PANTOSYN-PWY: su	-0.35989558	0.21360432	0.573499904	0.480062	0.425562	0.465151	1	1	1	1	1	1
PENTOSE-P-PWY: pe	0.616945699	0.172748433	-0.444197266	0.502484	0.445657	0.47969	1	1	1	1	1	1
PEPTIDOGLYCANSY	-0.26493523	0.23454003	0.499475262	0.487594	0.432312	0.470019	1	1	1	1	1	1
PHOSLIPSYN-PWY: s	0.394572721	-0.025797639	-0.42037036	0.484455	0.4295	0.467989	1	1	1	1	1	1
POLYAMINSYN3-PW	-2.66419564	-1.102025505	1.562170139	0.570705	0.506793	0.524705	3.04E-06	0.029667	0.002909	2.7E-05	0.204652	0.022973
POLYAMSYN-PWY: s	1.331127796	-0.240557441	-1.571685237	0.648683	0.57666	0.577359	1	1	1	1	1	1
POLYISOPRENSYN-I	0.479980654	-0.33855726	-0.818537915	0.438124	0.387975	0.438348	1	1	1	1	1	1
PPGPPMET-PWY: pp	0.273816245	0.295362136	0.021545892	0.533954	0.47386	0.500318	1	1	1	1	1	1
PWY-1042: glycolysi	-0.11483482	0.206530681	0.321365498	0.490957	0.435327	0.472198	1	1	1	1	1	1
PWY-1269: CMP-3-c	0.958027551	-0.154495817	-1.112523368	0.420273	0.371976	0.427106	1	1	1	1	1	1
PWY-1861: formalde	-0.98833259	-1.025746622	-0.037414034	0.724567	0.644639	0.629566	1	1	1	1	1	1
PWY-241: C4 photos	-0.57201861	-0.74375196	-0.171733346	0.619045	0.550107	0.557214	1	1	1	1	1	1
PWY-2941: L-lysine	-0.57378101	-0.620558977	-0.046777971	0.586676	0.521104	0.535395	1	1	1	1	1	1
PWY-2942: L-lysine	-0.01723215	0.270888091	0.288120245	0.487239	0.431994	0.46979	1	1	1	1	1	1
PWY-3001: superpa	0.172940208	0.013769897	-0.159170311	0.257966	0.226891	0.329037	1	1	1	1	1	1
PWY-3841: folate tra	-0.32728122	0.295711632	0.622992848	0.482421	0.427676	0.466674	1	1	1	1	1	1
PWY-4041: &gamma	0.68329481	0.179647541	-0.503647268	0.510209	0.452581	0.48473	1	1	1	1	1	1
PWY-4984: urea cyc	-0.33189803	0.410878855	0.742776887	0.620644	0.551539	0.558296	1	1	1	1	1	1

pathway	lfc_IBD	lfc_PD	lfc_PDvsIBD	se_IBD	se_PD	se_PDvsIBI	pval_IBD	pval_PD	pval_PDvsI	adj_pval_IB	adj_pval_PI	adj_pval_PDvsIBD
PWY-5004: superpa	-1.49720699	-0.262395278	1.234811716	0.475092	0.421107	0.461947	0.001625	0.533214	0.007516	0.014459	1	0.059366
PWY-5005: biotin bi	-2.40350865	-0.81870723	1.584801421	0.571171	0.507211	0.525016	2.58E-05	0.106498	0.00254	0.000229	0.734654	0.020059
PWY-5022: 4-amino	-1.75666562	0.36861917	2.125284786	0.564019	0.500801	0.520246	0.001842	0.461695	4.4E-05	0.014551	1	0.000392
PWY-5030: L-histidi	-2.08351852	-0.029105035	2.054413489	0.575445	0.51104	0.527872	0.000294	0.954583	9.95E-05	0.002321	1	0.000885
PWY-5097: L-lysine	-0.07140856	0.170471706	0.24188027	0.489674	0.434176	0.471366	1	1	1	1	1	1
PWY-5100: pyruvate	-0.13756376	0.182477045	0.320040803	0.483372	0.428529	0.467289	1	1	1	1	1	1
PWY-5103: L-isoleu	0.120335278	0.14900377	0.028668492	0.493231	0.437364	0.473673	1	1	1	1	1	1
PWY-5104: L-isoleu	-0.37612663	-0.196571787	0.179554845	0.693897	0.617166	0.608365	1	1	1	1	1	1
PWY-5121: superpa	-0.12385926	-0.044678688	0.079180569	0.400616	0.35436	0.414849	1	1	1	1	1	1
PWY-5130: 2-oxobu	-2.77184867	-0.011473034	2.760375634	0.581196	0.516193	0.53172	1.85E-06	0.982267	2.09E-07	1.46E-05	1	1.86E-06
PWY-5136: fatty aci	1.792285921	-0.463813063	-2.256098983	0.740961	0.659324	0.64095	0.015569	0.481765	0.000432	0.122968	1	0.003841
PWY-5138: fatty aci	2.519190358	-0.235889989	-2.755080347	0.697184	0.62011	0.610631	0.000302	0.703648	6.43E-06	0.002387	1	5.72E-05
PWY-5154: L-argini	0.773282957	-0.353499801	-1.126782758	0.451887	0.40031	0.447085	1	1	1	1	1	1
PWY-5188: tetrapyr	0.203841368	0.268147887	0.064306519	0.499304	0.442807	0.47762	1	1	1	1	1	1
PWY-5189: tetrapyr	0.56327781	0.057623377	-0.505654433	0.650264	0.578077	0.578438	1	1	1	1	1	1
PWY-5345: superpa	0.79706067	0.088889324	-0.708171346	0.757342	0.673997	0.652357	1	1	1	1	1	1
PWY-5347: superpa	2.011758438	-0.39485265	-2.406611089	0.789918	0.703175	0.675135	0.010872	0.574438	0.000364	0.085868	1	0.003242
PWY-5367: petrosel	1.440783996	-0.444542223	-1.885326219	0.625014	0.555455	0.561259	0.021155	0.423525	0.000782	0.167091	1	0.006958
PWY-5384: sucrose	0.743742752	-0.319114936	-1.062857688	0.386728	0.341916	0.40627	1	1	1	1	1	1
PWY-5484: glycolysi	0.187484208	0.102158929	-0.085325279	0.49364	0.437731	0.473939	1	1	1	1	1	1
PWY-5497: purine n	1.408523399	-0.206994994	-1.615518392	0.610548	0.542494	0.551467	1	1	1	1	1	1
PWY-5505: L-glutam	-1.97575578	-0.921927046	1.053828734	0.589486	0.523622	0.53728	0.000803	0.078294	0.049831	0.007148	0.540093	0.39358
PWY-561: superpath	1.818138191	-0.989088866	-2.807227057	0.80071	0.712841	0.682706	0.023168	0.16528	3.92E-05	0.182986	1	0.000349
PWY-5656: mannos	1.485830294	-0.101864339	-1.587694632	0.680194	0.60489	0.598936	1	1	1	1	1	1
PWY-5659: GDP-ma	0.208899577	0.120139587	-0.08875999	0.480433	0.425894	0.46539	1	1	1	1	1	1
PWY-5667: CDP-dia	-0.16534961	-0.092623802	0.072725805	0.466689	0.413577	0.456547	1	1	1	1	1	1
PWY-5675: nitrate r	2.307861336	-0.194285304	-2.50214664	0.757708	0.674325	0.652613	0.00232	0.773256	0.000126	0.018326	1	0.001122
PWY-5676: acetyl-C	-2.08443846	-0.691570296	1.392868164	0.625967	0.556309	0.561905	0.000869	0.213816	0.013181	0.007729	1	0.10411
PWY-5686: UMP bio	-0.42885749	0.206708115	0.635565601	0.486245	0.431103	0.469146	1	1	1	1	1	1
PWY-5690: TCA cycl	-1.67396572	0.021473972	1.695439695	0.612797	0.544509	0.552987	0.006301	0.968542	0.00217	0.049769	1	0.019306
PWY-5692: allantoi	1.289297457	-0.165392391	-1.454689848	0.397743	0.351786	0.413069	0.001189	0.638247	0.000429	0.00939	1	0.003816
PWY-5695: inosine 5	0.089402581	0.325583825	0.236181244	0.491036	0.435397	0.47225	1	1	1	1	1	1
PWY-5705: allantoi	1.560491079	-0.171701781	-1.732192861	0.482525	0.42777	0.466741	0.001221	0.688134	0.000206	0.009641	1	0.001835
PWY-5723: Rubisco	2.718412272	0.059937266	-2.658475005	0.845147	0.752639	0.714004	0.001298	0.936527	0.000197	0.010249	1	0.001749
PWY-5747: 2-methy	2.027657872	-0.205558582	-2.233216454	0.546045	0.484695	0.508307	0.000205	0.671494	1.12E-05	0.001615	1	9.93E-05
PWY-5837: 2-carbo	1.928079957	0.021483003	-1.906596954	0.681463	0.606027	0.599808	0.004665	0.971722	0.001479	0.036843	1	0.013165
PWY-5838: superpa	1.963922723	0.016893338	-1.947029385	0.771517	0.686694	0.662254	0.010911	0.980373	0.003282	0.086179	1	0.029205
PWY-5840: superpa	2.031749663	0.329523018	-1.702226644	0.780077	0.694361	0.668241	1	1	1	1	1	1
PWY-5845: superpa	2.701169435	0.331825324	-2.369344111	0.705869	0.627891	0.616626	0.00013	0.597169	0.000122	0.001084	1	0.001084

pathway	lfc_IBD	lfc_PD	lfc_PDvsIBD	se_IBD	se_PD	se_PDvsIBI	pval_IBD	pval_PD	pval_PDvsI	adj_pval_IB	adj_pval_PI	adj_pval_PDvsIBD
PWY-5850: superpa	2.44324134	-0.313614624	-2.756855964	0.729594	0.649143	0.633053	0.000812	0.62901	1.33E-05	0.006411	1	0.000118
PWY-5855: ubiquinc	2.154457833	-0.028477574	-2.182935407	0.525265	0.466073	0.494597	4.1E-05	0.951279	1.02E-05	0.000324	1	9.05E-05
PWY-5860: superpa	2.381059426	-0.292013087	-2.673072514	0.693805	0.617084	0.608302	0.000599	0.63606	1.11E-05	0.004734	1	9.89E-05
PWY-5861: superpa	2.002521139	0.028003566	-1.974517573	0.744595	0.66258	0.643478	0.007158	0.966288	0.002151	0.056535	1	0.019143
PWY-5862: superpa	2.615212165	0.302649022	-2.312563143	0.672447	0.59795	0.593617	0.000101	0.612756	9.79E-05	0.000871	1	0.000871
PWY-5896: superpa	2.44324134	-0.313614624	-2.756855964	0.729594	0.649143	0.633053	0.000812	0.62901	1.33E-05	0.006411	1	0.000118
PWY-5897: superpa	1.966231943	0.02577434	-1.940457603	0.769547	0.68493	0.660878	0.010617	0.969982	0.003323	0.083858	1	0.029567
PWY-5898: superpa	1.966231943	0.02577434	-1.940457603	0.769547	0.68493	0.660878	0.010617	0.969982	0.003323	0.083858	1	0.029567
PWY-5899: superpa	1.966231943	0.02577434	-1.940457603	0.769547	0.68493	0.660878	0.010617	0.969982	0.003323	0.083858	1	0.029567
PWY-5913: partial T(	1.02935204	-0.443567987	-1.472920027	0.567231	0.50368	0.522387	1	1	1	1	1	1
PWY-5918: superpa	0.843260141	-0.072721528	-0.915981669	0.697104	0.620038	0.610576	1	1	1	1	1	1
PWY-5920: superpa	2.294139252	0.024868858	-2.269270394	0.74408	0.662118	0.643119	0.002048	0.970039	0.000418	0.016175	1	0.003718
PWY-5941: glycoger	0.098230926	0.03961736	-0.058613566	0.501703	0.444957	0.479181	1	1	1	1	1	1
PWY-5971: palmitat	1.825356121	-0.441598005	-2.266954126	0.839465	0.747551	0.709992	0.029673	0.554704	0.001408	0.234367	1	0.012532
PWY-5973: cis-vacc	0.18015673	0.340660844	0.160504114	0.4873	0.432049	0.469829	1	1	1	1	1	1
PWY-5981: CDP-dia	0.884668752	0.373325983	-0.511342768	0.604103	0.536719	0.547117	1	1	1	1	1	1
PWY-5989: stearate	0.720730014	-0.095486235	-0.816216249	0.479634	0.425179	0.464875	1	1	1	1	1	1
PWY-6121: 5-amino	-0.18117595	0.186377612	0.36755356	0.485141	0.430114	0.468432	1	1	1	1	1	1
PWY-6122: 5-amino	-0.09228929	0.206603473	0.298892764	0.486358	0.431205	0.469219	1	1	1	1	1	1
PWY-6123: inosine-	-0.33306287	0.270002083	0.603064954	0.484981	0.429971	0.468329	1	1	1	1	1	1
PWY-6124: inosine-	-0.41822894	0.28188719	0.700116127	0.484223	0.429291	0.467838	1	1	1	1	1	1
PWY-6125: superpa	0.500449489	0.249724638	-0.250724851	0.397135	0.351241	0.412693	1	1	1	1	1	1
PWY-6126: superpa	0.383274171	0.239251582	-0.144022589	0.414248	0.366577	0.423335	1	1	1	1	1	1
PWY-6147: 6-hydro>	0.040819421	0.119464842	0.078645422	0.256984	0.226024	0.328429	1	1	1	1	1	1
PWY-6151: S-adeno	-0.09614669	0.137963303	0.23410999	0.482226	0.427502	0.466548	1	1	1	1	1	1
PWY-6163: chorism	-0.27772022	0.138343053	0.416063274	0.487674	0.432385	0.470071	1	1	1	1	1	1
PWY-6168: flavin bi	1.744873653	0.443202397	-1.301671256	0.877787	0.781871	0.737109	1	1	1	1	1	1
PWY-621: sucrose d	0.88752557	-0.110925363	-0.998450934	0.396042	0.350262	0.412016	1	1	1	1	1	1
PWY-6270: isoprene	-0.41226531	0.089516069	0.50178138	0.246304	0.216642	0.321584	1	1	1	1	1	1
PWY-6277: superpa	-0.09228929	0.206603473	0.298892764	0.486358	0.431205	0.469219	1	1	1	1	1	1
PWY-6282: palmitol	0.651262395	-0.084201654	-0.735464049	0.490523	0.434937	0.471917	1	1	1	1	1	1
PWY-6284: superpa	1.652803299	-0.464965792	-2.117769091	0.730388	0.649854	0.633604	0.023641	0.474304	0.000831	0.186724	1	0.00739
PWY-6285: superpa	2.801724074	-0.085681376	-2.88740545	0.778058	0.692553	0.666828	0.000317	0.901538	1.49E-05	0.002504	1	0.000133
PWY-6292: superpa	-2.04229749	-0.819183381	1.223114112	0.599108	0.532243	0.54375	0.000652	0.123776	0.024487	0.005804	0.853845	0.193405
PWY-6293: superpa	0.497465147	-0.77268474	-1.270149887	0.618641	0.549745	0.55694	1	1	1	1	1	1
PWY-6305: superpa	1.275922055	0.054404418	-1.221517637	0.547074	0.485617	0.508989	1	1	1	1	1	1
PWY-6317: D-galact	0.012993419	-0.180649369	-0.193642788	0.274046	0.241149	0.33877	1	1	1	1	1	1
PWY-6318: L-phenyl	1.022094889	-0.241398338	-1.263493227	0.426313	0.37739	0.430898	1	1	1	1	1	1
PWY-6328: L-lysine	1.145014509	0.113081389	-1.031933119	0.516943	0.458615	0.489136	1	1	1	1	1	1

pathway	lfc_IBD	lfc_PD	lfc_PDvsIBD	se_IBD	se_PD	se_PDvsIBD	pval_IBD	pval_PD	pval_PDvsI	adj_pval_IB	adj_pval_PI	adj_pval_PDvsIBD
PWY-6353: purine n	0.342668183	-0.01069197	-0.353360153	0.492909	0.437076	0.473464	1	1	1	1	1	1
PWY-6385: peptidog	-0.19445344	0.236719357	0.431172797	0.487162	0.431926	0.46974	1	1	1	1	1	1
PWY-6386: UDP-N-a	-0.32977497	0.238764049	0.568539022	0.48927	0.433815	0.471105	1	1	1	1	1	1
PWY-6387: UDP-N-a	-0.26116335	0.237095558	0.498258908	0.487916	0.432601	0.470228	1	1	1	1	1	1
PWY-6470: peptidog	-3.52889313	-1.278221689	2.250671438	0.703414	0.625692	0.614931	5.25E-07	0.041063	0.000252	4.67E-06	0.283265	0.001992
PWY-6507: 4-deoxy-	0.020575427	-0.462786918	-0.483362345	0.431307	0.381865	0.434043	1	1	1	1	1	1
PWY-6519: 8-amino	0.719629555	-0.412586946	-1.1322165	0.581623	0.516576	0.532007	1	1	1	1	1	1
PWY-6527: stachyos	0.161022753	-0.251332427	-0.41235518	0.313426	0.276283	0.36207	1	1	1	1	1	1
PWY-6531: mannito	1.713297308	-0.477098674	-2.190395982	0.695341	0.618459	0.609361	0.013741	0.440452	0.000325	0.10853	1	0.002891
PWY-6545: pyrimidi	1.163242377	-0.598483988	-1.761726364	0.625923	0.556269	0.561875	0.063106	0.281976	0.001716	0.49843	1	0.015269
PWY-6549: L-glutam	-2.04567258	-1.022259694	1.023412886	0.635144	0.56453	0.568137	0.001278	0.07017	0.071648	0.011375	0.554221	0.554221
PWY-6588: pyruvate	-0.33800443	-0.476567935	-0.138563502	0.656045	0.583256	0.582386	1	1	1	1	1	1
PWY-6590: superpat	-2.89625699	-0.867986121	2.028270866	0.612466	0.544212	0.552763	2.26E-06	0.110725	0.000243	2.01E-05	0.763818	0.001921
PWY-6595: superpat	-0.25082814	0.546487987	0.797316126	0.589116	0.52329	0.537032	1	1	1	1	1	1
PWY-6606: guanosi	0.496789218	-0.007168949	-0.503958167	0.432086	0.382563	0.434534	1	1	1	1	1	1
PWY-6607: guanosi	-0.28695594	0.536632501	0.823588444	0.590775	0.524776	0.538146	1	1	1	1	1	1
PWY-6608: guanosi	0.268110403	0.058473907	-0.209636495	0.382207	0.337864	0.403492	1	1	1	1	1	1
PWY-6609: adenine	-0.28955504	0.170107049	0.459662091	0.483223	0.428395	0.467192	1	1	1	1	1	1
PWY-6612: superpat	0.721829625	-1.052001701	-1.773831326	0.628906	0.558941	0.563899	0.25107	0.059818	0.001657	1	0.472461	0.014746
PWY-6628: superpat	0.24351993	-0.153078429	-0.396598359	0.358443	0.316575	0.389005	1	1	1	1	1	1
PWY-6630: superpat	0.912071706	0.220206942	-0.691864764	0.514918	0.4568	0.48781	1	1	1	1	1	1
PWY-6690: cinnama	2.97847032	0.163515319	-2.814955	0.643734	0.572227	0.573985	3.71E-06	0.775068	9.38E-07	2.93E-05	1	8.35E-06
PWY-6700: queuosi	-0.15496123	0.28344095	0.43840218	0.480557	0.426005	0.46547	1	1	1	1	1	1
PWY-6703: preQ0 bi	-0.18819038	0.1446346	0.332824984	0.460315	0.407863	0.452464	1	1	1	1	1	1
PWY-6708: ubiquino	1.628472083	-0.100075561	-1.728547644	0.468164	0.414898	0.457493	0.000504	0.809396	0.000158	0.003984	1	0.001405
PWY-6731: starch de	0.365118141	-0.345739705	-0.710857845	0.418557	0.370439	0.426031	1	1	1	1	1	1
PWY-6749: CMP-leg	-0.68001862	-0.056428486	0.623590135	0.523222	0.464243	0.493255	1	1	1	1	1	1
PWY-6803: phospha	2.204471029	-0.305180805	-2.509651834	0.78663	0.70023	0.67283	0.005072	0.662961	0.000191	0.04006	1	0.001704
PWY-6823: molybdo	-0.32453458	-0.028576713	0.29595787	0.499946	0.443382	0.478037	1	1	1	1	1	1
PWY-6859: all-trans	0.163113225	-0.260720749	-0.423833974	0.40724	0.360297	0.418965	1	1	1	1	1	1
PWY-6895: superpat	0.370223614	-0.857848502	-1.228072116	0.710865	0.632366	0.620079	1	1	1	1	1	1
PWY-6897: thiamine	-0.04839359	0.060923834	0.109317428	0.484697	0.429716	0.468145	1	1	1	1	1	1
PWY-6901: superpat	0.315853223	-0.108693789	-0.424547012	0.395943	0.350173	0.411955	1	1	1	1	1	1
PWY-6902: chitin de	0.228604717	-0.126896893	-0.355501611	0.423175	0.374577	0.428927	1	1	1	1	1	1
PWY-6906: chitin de	-0.38304101	-0.402323256	-0.019282245	0.712001	0.633384	0.620865	1	1	1	1	1	1
PWY-6922: L-N&del	0.500984199	0.004735853	-0.496248346	0.567279	0.503723	0.522419	1	1	1	1	1	1
PWY-6936: seleno-a	0.432416945	-0.044400483	-0.476817428	0.324898	0.286543	0.368879	1	1	1	1	1	1
PWY-6961: L-ascorb	2.341562182	-0.32957291	-2.671135092	0.669413	0.595232	0.591536	0.000469	0.579792	6.31E-06	0.003703	1	5.62E-05
PWY-6969: TCA cycl	0.264623364	0.05474129	-0.209882074	0.353917	0.312521	0.386268	1	1	1	1	1	1

pathway	lfc_IBD	lfc_PD	lfc_PDvsIBD	se_IBD	se_PD	se_PDvsIBI	pval_IBD	pval_PD	pval_PDvsI	adj_pval_IB	adj_pval_PI	adj_pval_PDvsIBD
PWY-6992: 1,5-anhy	1.010065519	-0.192333158	-1.202398677	0.486429	0.431269	0.469266	1	1	1	1	1	1
PWY-7013: (S)-prop	0.565631834	-0.32040834	-0.886040175	0.744772	0.662738	0.643601	1	1	1	1	1	1
PWY-702: L-methior	1.083807982	-0.101426237	-1.185234219	0.420807	0.372455	0.42744	1	1	1	1	1	1
PWY-7094: fatty aci	2.051737026	-0.403541197	-2.455278223	0.623067	0.55371	0.559938	0.000991	0.466127	1.16E-05	0.00783	1	0.000103
PWY-7111: pyruvate	0.432535117	0.113728845	-0.318806272	0.501381	0.444668	0.478971	1	1	1	1	1	1
PWY-7115: C4 phot	0.656336877	-0.601790735	-1.258127612	0.627041	0.557271	0.562634	1	1	1	1	1	1
PWY-7117: C4 phot	1.226629472	-0.495133259	-1.721762731	0.570231	0.506369	0.524389	0.031468	0.328167	0.001026	0.248541	1	0.009127
PWY-7118: chitin de	2.114376212	-0.472985622	-2.587361834	0.673333	0.598744	0.594225	0.001689	0.429549	1.34E-05	0.013337	1	0.000119
PWY-7184: pyrimidi	1.529342089	-0.558497526	-2.087839616	0.651216	0.57893	0.579088	0.018852	0.334692	0.000312	0.148901	1	0.002773
PWY-7197: pyrimidi	0.453075713	0.282913105	-0.170162608	0.362547	0.320251	0.391493	1	1	1	1	1	1
PWY-7198: pyrimidi	0.203205687	0.281529535	0.078323848	0.395255	0.349556	0.41153	1	1	1	1	1	1
PWY-7199: pyrimidi	-0.34133515	0.202709315	0.544044463	0.481611	0.42695	0.466151	1	1	1	1	1	1
PWY-7204: pyridoxa	2.079720852	0.360477042	-1.71924381	0.714027	0.635198	0.622266	0.003584	0.570372	0.005729	0.031888	1	0.045253
PWY-7208: superpa	0.116032556	0.081530379	-0.034502177	0.311924	0.27494	0.361181	1	1	1	1	1	1
PWY-7209: superpa	0.968629635	-0.166883237	-1.135512872	0.549718	0.487987	0.510741	1	1	1	1	1	1
PWY-7210: pyrimidi	1.691150296	-0.326698864	-2.01784916	0.65416	0.581567	0.581098	0.009732	0.574282	0.000516	0.076863	1	0.004589
PWY-7211: superpa	1.015608039	-0.969077253	-1.984685291	0.619881	0.550856	0.55778	0.10134	0.07854	0.000373	0.699076	0.620333	0.003323
PWY-7220: adenosi	0.619793513	0.267222365	-0.352571148	0.38368	0.339184	0.404396	1	1	1	1	1	1
PWY-7221: guanosi	-0.44687935	0.220712452	0.6675918	0.482091	0.427381	0.466461	1	1	1	1	1	1
PWY-7222: guanosi	0.619793513	0.267222365	-0.352571148	0.38368	0.339184	0.404396	1	1	1	1	1	1
PWY-7228: superpa	0.474484076	0.249667377	-0.224816699	0.40691	0.36	0.418759	1	1	1	1	1	1
PWY-7229: superpa	0.295181755	0.231457256	-0.063724499	0.432749	0.383157	0.434952	1	1	1	1	1	1
PWY-7234: inosine-	-0.06870326	-0.269177554	-0.200474291	0.500754	0.444107	0.478563	1	1	1	1	1	1
PWY-7237: myo-, ch	-1.59145781	-1.818392064	-0.226934252	0.941121	0.838587	0.782188	1	1	1	1	1	1
PWY-7238: sucrose	0.039846289	0.032691371	-0.007154918	0.507333	0.450003	0.482852	0.937398	0.942087	0.988177	1	1	1
PWY-724: superpath	-0.01770359	0.136747657	0.154451242	0.488482	0.433108	0.470594	1	1	1	1	1	1
PWY-7242: D-fructu	0.074316854	-0.374991909	-0.449308763	0.441114	0.390655	0.440241	1	1	1	1	1	1
PWY-7254: TCA cycl	-1.67024976	0.36057701	2.030826773	0.655995	0.583211	0.582352	0.010892	0.536403	0.000488	0.086031	1	0.004342
PWY-7269: mitocho	1.912939459	-0.111995271	-2.02493473	0.559451	0.496708	0.517205	0.000628	0.82161	9.03E-05	0.004958	1	0.000804
PWY-7282: 4-amino	0.234376109	0.021926201	-0.212449908	0.454567	0.402712	0.448793	1	1	1	1	1	1
PWY-7315: dTDP-N-	1.175690951	-0.243351978	-1.419042929	0.649598	0.57748	0.577983	1	1	1	1	1	1
PWY-7323: superpa	-0.11772019	-0.031659761	0.086060433	0.301676	0.265782	0.355123	1	1	1	1	1	1
PWY-7328: superpa	-0.11941154	0.406359591	0.525771135	0.338372	0.298602	0.376921	1	1	1	1	1	1
PWY-7345: superpa	0.494396641	-0.48605646	-0.980453102	0.595436	0.528953	0.54128	1	1	1	1	1	1
PWY-7356: thiamine	-1.45921457	-0.863384781	0.595829787	0.594464	0.528082	0.540626	1	1	1	1	1	1
PWY-7357: thiamine	-0.23551732	-0.070438762	0.16507856	0.492639	0.436834	0.473289	1	1	1	1	1	1
PWY-7383: anaerob	-3.72376816	0.0388978	3.762665961	0.563948	0.500738	0.520198	4.03E-11	0.938082	4.72E-13	3.18E-10	1	4.2E-12
PWY-7385: 1,3-prop	1.310885773	-1.012447598	-2.323333371	0.638497	0.567534	0.570418	0.040065	0.074433	4.64E-05	0.316443	0.513463	0.000413
PWY-7388: octanoy	1.30649585	-0.298435776	-1.604931626	0.654919	0.582247	0.581617	1	1	1	1	1	1

pathway	lfc_IBD	lfc_PD	lfc_PDvsIBD	se_IBD	se_PD	se_PDvsIBI	pval_IBD	pval_PD	pval_PDvsI	adj_pval_IB	adj_pval_PI	adj_pval_PDvsIBD
PWY-7392: taxadien	-1.58758859	-0.408053891	1.1795347	0.635086	0.564479	0.568098	1	1	1	1	1	1
PWY-7400: L-argini	1.161401669	0.36256308	-0.798838589	0.624398	0.554903	0.560841	1	1	1	1	1	1
PWY-7409: phospho	1.805400606	0.295084028	-1.510316578	0.45064	0.399192	0.446291	6.17E-05	0.459784	0.000714	0.000549	1	0.005639
PWY-7456: β-(	-2.00699105	-1.051814394	0.955176659	0.622533	0.553232	0.559577	0.001265	0.057273	0.087829	0.011252	0.452363	0.605871
PWY-7560: methyle	-0.07201195	0.108583493	0.180595439	0.238387	0.2098	0.315914	1	1	1	1	1	1
PWY-7616: methano	1.522555746	-0.0525955	-1.575151246	0.382602	0.338218	0.403734	6.91E-05	0.876421	9.56E-05	0.000615	1	0.000755
PWY-7663: gondoat	0.614971229	0.320694118	-0.294277111	0.491158	0.435507	0.472329	1	1	1	1	1	1
PWY-7664: oleate bi	0.622704219	-0.087573266	-0.710277486	0.494351	0.438368	0.4744	1	1	1	1	1	1
PWY-7761: NAD salv	-0.30614341	-0.285276244	0.020867167	0.425084	0.376288	0.430126	1	1	1	1	1	1
PWY-7790: UMP bio	-0.46926629	0.204476154	0.673742445	0.485583	0.43051	0.468718	1	1	1	1	1	1
PWY-7791: UMP bio	-0.47009666	0.204508002	0.674604658	0.485674	0.430591	0.468777	1	1	1	1	1	1
PWY-7805: (aminom	1.82731658	0.17171054	-1.655606039	0.463879	0.411058	0.454746	8.18E-05	0.676146	0.000272	0.000727	1	0.002147
PWY-7807: glyphosa	1.794567245	0.076611136	-1.717956109	0.472904	0.419146	0.460539	0.000148	0.854972	0.000191	0.001315	1	0.00151
PWY-7851: coenzym	-0.47951324	0.186329536	0.665842778	0.483986	0.429079	0.467685	1	1	1	1	1	1
PWY-7858: (5Z)-doc	2.704786775	-0.167465151	-2.872251926	0.697905	0.620756	0.611129	0.000106	0.787333	2.6E-06	0.00084	1	2.32E-05
PWY-7874: L-threon	1.355688291	0.073086043	-1.282602247	0.435679	0.385784	0.436802	0.00186	0.849742	0.003321	0.016554	1	0.02623
PWY-7883: anhydro	1.482570641	-0.36171356	-1.844284201	0.622092	0.552836	0.559277	0.017163	0.512927	0.000975	0.135556	1	0.008677
PWY-7942: 5-oxo-L-	0.709222439	-0.929461984	-1.638684423	0.640572	0.569394	0.571831	1	1	1	1	1	1
PWY-7953: UDP-N-a	-0.27445406	0.21353436	0.487988417	0.491738	0.436026	0.472705	1	1	1	1	1	1
PWY-7977: L-methic	0.331036736	0.126318119	-0.204718617	0.235801	0.207624	0.313725	1	1	1	1	1	1
PWY-8004: Entner-D	0.714289053	-0.056623972	-0.770913025	0.452191	0.400583	0.447279	1	1	1	1	1	1
PWY-801: homocyst	0.960627967	-0.676929753	-1.63755772	0.586856	0.521265	0.535515	1	1	1	1	1	1
PWY-8073: lipid IVA	0.704516038	-0.059579214	-0.764095252	0.548602	0.486987	0.510001	1	1	1	1	1	1
PWY-8131: 5'-deoxy	-0.25526039	0.107545856	0.362806242	0.554273	0.492069	0.513763	1	1	1	1	1	1
PWY-8178: pentose	0.042539766	0.165282492	0.122742726	0.490807	0.435192	0.472101	1	1	1	1	1	1
PWY-8187: L-argini	-0.01757282	0.424435745	0.442008566	0.491214	0.435557	0.472365	1	1	1	1	1	1
PWY-821: superpath	1.599281812	0.01660753	-1.582674282	0.804764	0.716472	0.685553	1	1	1	1	1	1
PWY-841: superpath	0.363191047	0.22570938	-0.137481667	0.41752	0.36951	0.425382	1	1	1	1	1	1
PWY-I9: L-cysteine b	0.755725122	-0.161587379	-0.917312501	0.431061	0.381645	0.433887	1	1	1	1	1	1
PWY0-1061: superp	0.102779843	-0.720938807	-0.82371865	0.620325	0.551253	0.55808	1	1	1	1	1	1
PWY0-1241: ADP-L-	1.184501667	0.171718482	-1.012783185	0.571484	0.507491	0.525225	1	1	1	1	1	1
PWY0-1261: anhydr	1.163246798	-0.183658934	-1.346905731	0.535966	0.475663	0.501644	1	1	1	1	1	1
PWY0-1277: 3-phen	2.971231628	0.005414778	-2.96581685	0.676148	0.601266	0.596157	1.11E-05	0.992815	6.53E-07	8.78E-05	1	5.81E-06
PWY0-1296: purine	0.394673941	0.276699473	-0.117974468	0.318543	0.280858	0.365103	1	1	1	1	1	1
PWY0-1297: superp	0.56500248	-0.214131026	-0.779133506	0.566463	0.502992	0.521875	1	1	1	1	1	1
PWY0-1298: superp	0.725915692	-0.212389047	-0.938304739	0.447608	0.396475	0.444362	1	1	1	1	1	1
PWY0-1319: CDP-di	-0.1659984	-0.091091506	0.074906894	0.466488	0.413397	0.456418	1	1	1	1	1	1
PWY0-1337: oleate	2.24891442	-0.254799746	-2.503714166	0.624634	0.555114	0.561001	0.000318	0.646231	8.08E-06	0.00251	1	7.19E-05
PWY0-1338: polymy	2.522506171	-0.243456244	-2.765962414	0.62557	0.555953	0.561636	5.52E-05	0.661453	8.44E-07	0.000436	1	7.51E-06

pathway	lfc_IBD	lfc_PD	lfc_PDvsIBD	se_IBD	se_PD	se_PDvsIBI	pval_IBD	pval_PD	pval_PDvsI	adj_pval_IB	adj_pval_PI	adj_pval_PDvsIBD
PWY0-1415: superp	2.533025961	-0.206975082	-2.740001043	0.736732	0.655536	0.63801	0.000586	0.752204	1.75E-05	0.004625	1	0.000156
PWY0-1477: ethano	1.02994849	-0.22810086	-1.25804935	0.534479	0.47433	0.500664	1	1	1	1	1	1
PWY0-1479: tRNA p	1.134593291	-0.347421693	-1.482014984	0.735593	0.654517	0.637219	1	1	1	1	1	1
PWY0-1533: methyl	1.462360524	0.083376048	-1.378984477	0.417104	0.369136	0.425121	0.000455	0.821304	0.00118	0.004048	1	0.009318
PWY0-1586: peptido	0.517947835	-0.039573888	-0.557521722	0.491711	0.436002	0.472687	1	1	1	1	1	1
PWY0-162: superpa	0.466945063	0.158135094	-0.30880997	0.421705	0.37326	0.428004	1	1	1	1	1	1
PWY0-166: superpa	2.276494649	-0.224683206	-2.501177854	0.693363	0.616687	0.607997	0.001026	0.715605	3.89E-05	0.008105	1	0.000346
PWY0-301: L-ascorb	3.020824903	0.07418019	-2.946644713	0.632869	0.562493	0.566591	1.81E-06	0.895081	1.99E-07	1.43E-05	1	1.77E-06
PWY0-41: allantoin	1.372088336	-0.287006515	-1.659094851	0.440956	0.390513	0.440141	0.001861	0.462372	0.000164	0.014696	1	0.001456
PWY0-42: 2-methylc	2.034023892	-0.406017578	-2.440041469	0.66782	0.593805	0.590444	0.002321	0.49413	3.59E-05	0.018331	1	0.000319
PWY0-461: L-lysine	1.870458258	0.065374927	-1.805083331	0.480422	0.425885	0.465383	9.89E-05	0.878001	0.000105	0.00088	1	0.00088
PWY0-781: aspartat	1.626010884	-0.642753068	-2.268763953	0.773129	0.688138	0.663381	0.035452	0.35028	0.000626	0.280012	1	0.005572
PWY0-845: superpa	0.847524723	0.537646981	-0.309877742	0.501557	0.444826	0.479086	1	1	1	1	1	1
PWY0-862: (5Z)-doc	0.591625829	-0.101062434	-0.692688263	0.492025	0.436284	0.472891	1	1	1	1	1	1
PWY1G-0: mycothio	-1.27988587	0.130367521	1.410253392	0.589036	0.523219	0.536978	1	1	1	1	1	1
PWY1ZNC-1: assimi	2.015786963	0.335524429	-1.680262534	0.65324	0.580743	0.58047	0.00203	0.563433	0.003796	0.018062	1	0.029979
PWY3O-4107: NAD s	-0.61979659	-1.62365017	-1.003853576	0.714745	0.635841	0.622763	1	1	1	1	1	1
PWY4FS-7: phospha	0.542292267	0.112553946	-0.42973832	0.413346	0.365768	0.422772	1	1	1	1	1	1
PWY4FS-8: phospha	0.542402581	0.1122126	-0.430189982	0.413343	0.365766	0.42277	1	1	1	1	1	1
PWY4LZ-257: super	0.795352267	-0.578061094	-1.373413361	0.654717	0.582067	0.581479	1	1	1	1	1	1
PWY66-389: phytol	0.774941357	0.230961039	-0.543980318	0.710918	0.632413	0.620116	1	1	1	1	1	1
PWY66-391: fatty ac	-0.35534471	0.43955118	0.794895885	0.585163	0.519749	0.53438	1	1	1	1	1	1
PWY66-399: glucon	-4.02995477	-0.284063296	3.745891478	0.619993	0.550956	0.557856	8.03E-11	0.606146	1.88E-11	6.34E-10	1	1.68E-10
PWY66-409: superp	0.908646205	0.177252282	-0.731393923	0.637738	0.566855	0.569902	1	1	1	1	1	1
PWY66-429: fatty ac	-0.079825	-0.124905687	-0.045080687	0.49817	0.441791	0.476882	1	1	1	1	1	1
PWY66-430: myrista	1.304827186	-0.300681567	-1.605508753	0.655733	0.582976	0.582172	1	1	1	1	1	1
PYRIDNUCSAL-PWY	-0.7055049	-1.824465161	-1.118960256	0.716979	0.637843	0.62431	1	1	1	1	1	1
PYRIDNUCSYN-PWY	-0.08549183	0.215858796	0.301350623	0.4826	0.427836	0.466789	1	1	1	1	1	1
PYRIDOXSYN-PWY:	0.459889196	0.311996592	-0.147892604	0.439681	0.389371	0.439334	1	1	1	1	1	1
REDCITCYC: TCA cy	0.506000365	-0.7415848	-1.247585165	0.669834	0.595609	0.591825	1	1	1	1	1	1
RHAMCAT-PWY: L-rh	0.451883647	-0.370861041	-0.822744687	0.440958	0.390515	0.440142	1	1	1	1	1	1
RIBOSYN2-PWY: flav	-0.56380667	0.077794963	0.641601632	0.280717	0.247085	0.342736	1	1	1	1	1	1
SALVADEHYPOX-PW	0.46452419	-0.218395594	-0.682919784	0.512399	0.454543	0.486162	1	1	1	1	1	1
SER-GLYSYN-PWY: s	0.111655175	0.314592812	0.202937637	0.480798	0.426222	0.465626	1	1	1	1	1	1
SO4ASSIM-PWY: ass	1.968544503	0.027627627	-1.940916876	0.687785	0.61169	0.604156	0.004208	0.963975	0.001315	0.033234	1	0.011704
SULFATE-CYS-PWY:	1.766817773	0.047063757	-1.719754017	0.721238	0.641658	0.627259	1	1	1	1	1	1
TCA-GLYOX-BYPASS	1.663827401	-0.918939563	-2.582766964	0.801242	0.713317	0.683079	0.037842	0.197655	0.000156	0.298888	1	0.00139
TCA: TCA cycle I (pro	1.288162912	-0.389454768	-1.67761768	0.794382	0.707174	0.678266	1	1	1	1	1	1
THISYNARA-PWY: su	-1.14617967	-0.41186879	0.73431088	0.463249	0.410493	0.454342	1	1	1	1	1	1

pathway	lfc_IBD	lfc_PD	lfc_PDvsIBD	se_IBD	se_PD	se_PDvsIBI	pval_IBD	pval_PD	pval_PDvsI	adj_pval_IB	adj_pval_PI	adj_pval_PDvsIBD
THRESYN-PWY: sup	0.156161001	0.143289283	-0.012871718	0.486654	0.43147	0.469411	1	1	1	1	1	1
TRNA-CHARGING-P\	-0.1965962	0.226725308	0.423321509	0.489583	0.434095	0.471308	1	1	1	1	1	1
UBISYN-PWY: super	1.668063576	-0.106731936	-1.774795512	0.480648	0.426087	0.465529	0.00052	0.802206	0.000138	0.004104	1	0.001224
UDPNAGSYN-PWY: l	0.292744912	0.199263122	-0.09348179	0.292878	0.257927	0.349928	1	1	1	1	1	1
URDEGR-PWY: supe	1.289297457	-0.165392391	-1.454689848	0.397743	0.351786	0.413069	0.001189	0.638247	0.000429	0.00939	1	0.003816
VALSYN-PWY: L-vali	0.156760283	0.11837263	-0.038387653	0.496177	0.440005	0.475586	1	1	1	1	1	1

Supplementary Table 4: UFPF all significant PD and IBD-associated MetaCyc pathways ANCOM-BC2 output

Differential abundances of MetaCyc pathways in PD (N = 54) vs. IBD (N = 26) vs. Healthy Control (N=16) were tested using ANCOM-BC2, while adjusting for covariates (diagnosis and total sequence count per sample). Shown are all the significantly enriched and depleted pathways are associated with IBD. Note: there were no significantly enriched or depleted pathways in the UFPF PD cohort. These pathways were detected by HUMAnN, however, only pathways that were present in at least 25% of samples were included in ANCOM-BC2 analysis. LFC: indicates the log fold change compared to healthy control or in the case of lfc_PDvsIBD it is the log fold change of PD compared to IBD. SE: standard error; Pval: the uncorrected, two-sided P-value reported by ANCOM-BC2; Adj. Pval aka q-value from the false discovery rate, i.e., multiple-testing corrected significance q-value, calculated using the Benjamini-Hochberg method, alpha =0.05. These tables correspond to the venn diagrams depicted in Figure 1C-D in the manuscript.

**IBD-Depleted Pathways in UFPF Cohort**												
pathway	lfc_IBD	lfc_PD	lfc_PDvsIBI	se_IBD	se_PD	se_PDvsIBI	pval_IBD	pval_PD	pval_PDvsI	adj_pval_IB	adj_pval_PI	adj_pval_PDvsIBD
PWY66-399: gluconeogenesis III	-4.02995	-0.28406	3.745891	0.619993	0.550956	0.557856	8.03E-11	0.606146	1.88E-11	6.34E-10	1	1.68E-10
PWY-7383: anaerobic energy metabolism (invertebrates	-3.72377	0.038898	3.762666	0.563948	0.500738	0.520198	4.03E-11	0.938082	4.72E-13	3.18E-10	1	4.2E-12
PWY-6470: peptidoglycan biosynthesis V (β-lactam	-3.52889	-1.27822	2.250671	0.703414	0.625692	0.614931	5.25E-07	0.041063	0.000252	4.67E-06	0.283265	0.001992
PWY-6590: superpathway of Clostridium acetobutylicun	-2.89626	-0.86799	2.028271	0.612466	0.544212	0.552763	2.26E-06	0.110725	0.000243	2.01E-05	0.763818	0.001921
CENTFERM-PWY: pyruvate fermentation to butanoate	-2.78822	-0.82944	1.958777	0.577726	0.513084	0.529397	1.39E-06	0.10597	0.000216	1.24E-05	0.731015	0.001703
PWY-5130: 2-oxobutanoate degradation I	-2.77185	-0.01147	2.760376	0.581196	0.516193	0.53172	1.85E-06	0.982267	2.09E-07	1.46E-05	1	1.86E-06
POLYAMINSYN3-PWY: superpathway of polyamine biosy	-2.6642	-1.10203	1.56217	0.570705	0.506793	0.524705	3.04E-06	0.029667	0.002909	2.7E-05	0.204652	0.022973
PWY-5005: biotin biosynthesis II	-2.40351	-0.81871	1.584801	0.571171	0.507211	0.525016	2.58E-05	0.106498	0.00254	0.000229	0.734654	0.020059
PWY-5676: acetyl-CoA fermentation to butanoate II	-2.08444	-0.69157	1.392868	0.625967	0.556309	0.561905	0.000869	0.213816	0.013181	0.007729	1	0.10411
PWY-5030: L-histidine degradation III	-2.08352	-0.02911	2.054413	0.575445	0.51104	0.527872	0.000294	0.954583	9.95E-05	0.002321	1	0.000885
PWY-6549: L-glutamine biosynthesis III	-2.04567	-1.02226	1.023413	0.635144	0.56453	0.568137	0.001278	0.07017	0.071648	0.011375	0.554221	0.554221
PWY-6292: superpathway of L-cysteine biosynthesis (m	-2.0423	-0.81918	1.223114	0.599108	0.532243	0.54375	0.000652	0.123776	0.024487	0.005804	0.853845	0.193405
PWY-7456: β-(1,4)-mannan degradation	-2.00699	-1.05181	0.955177	0.622533	0.553232	0.559577	0.001265	0.057273	0.087829	0.011252	0.452363	0.605871
PWY-5505: L-glutamate and L-glutamine biosynthesis	-1.97576	-0.92193	1.053829	0.589486	0.523622	0.53728	0.000803	0.078294	0.049831	0.007148	0.540093	0.39358
PWY-5022: 4-aminobutanoate degradation V	-1.75667	0.368619	2.125285	0.564019	0.500801	0.520246	0.001842	0.461695	4.4E-05	0.014551	1	0.000392
LACTOSECAT-PWY: lactose and galactose degradation I	-1.72587	-0.44498	1.280889	0.505359	0.448233	0.481563	0.000638	0.320837	0.007817	0.005673	1	0.061745
PWY-5690: TCA cycle II (plants and fungi)	-1.67397	0.021474	1.69544	0.612797	0.544509	0.552987	0.006301	0.968542	0.00217	0.049769	1	0.019306
PWY-5004: superpathway of L-citrulline metabolism	-1.49721	-0.2624	1.234812	0.475092	0.421107	0.461947	0.001625	0.533214	0.007516	0.014459	1	0.059366
HSERMETANA-PWY: L-methionine biosynthesis III	-1.12532	0.178442	1.303764	0.334497	0.295133	0.374602	0.000768	0.545435	0.000501	0.006063	1	0.004455
**IBD-Enriched Pathways in UFPF Cohort**												
pathway	lfc_IBD	lfc_PD	lfc_PDvsIBI	se_IBD	se_PD	se_PDvsIBI	pval_IBD	pval_PD	pval_PDvsI	adj_pval_IB	adj_pval_PI	adj_pval_PDvsIBD
PWY-5692: allantoin degradation to glyoxylate II	1.289297	-0.16539	-1.45469	0.397743	0.351786	0.413069	0.001189	0.638247	0.000429	0.00939	1	0.003816
URDEGR-PWY: superpathway of allantoin degradation ir	1.289297	-0.16539	-1.45469	0.397743	0.351786	0.413069	0.001189	0.638247	0.000429	0.00939	1	0.003816
PWY-7874: L-threonate degradation	1.355688	0.073086	-1.2826	0.435679	0.385784	0.436802	0.00186	0.849742	0.003321	0.016554	1	0.02623
PWY0-41: allantoin degradation IV (anaerobic)	1.372088	-0.28701	-1.65909	0.440956	0.390513	0.440141	0.001861	0.462372	0.000164	0.014696	1	0.001456
PWY0-1533: methylphosphonate degradation I	1.462361	0.083376	-1.37898	0.417104	0.369136	0.425121	0.000455	0.821304	0.00118	0.004048	1	0.009318
PWY-7616: methanol oxidation to carbon dioxide	1.522556	-0.0526	-1.57515	0.382602	0.338218	0.403734	6.91E-05	0.876421	9.56E-05	0.000615	1	0.000755
PWY-5705: allantoin degradation to glyoxylate III	1.560491	-0.1717	-1.73219	0.482525	0.42777	0.466741	0.001221	0.688134	0.000206	0.009641	1	0.001835
NAD-BIOSYNTHESIS-II: NAD salvage pathway III (to nico	1.61779	-0.18116	-1.79894	0.556164	0.493763	0.51502	0.003628	0.713703	0.000478	0.028653	1	0.004251
PWY-6708: ubiquinol-8 biosynthesis (early decarboxylat	1.628472	-0.10008	-1.72855	0.468164	0.414898	0.457493	0.000504	0.809396	0.000158	0.003984	1	0.001405
UBISYN-PWY: superpathway of ubiquinol-8 biosynthesis	1.668064	-0.10673	-1.7748	0.480648	0.426087	0.465529	0.00052	0.802206	0.000138	0.004104	1	0.001224

IBD-Enriched Pathways in UFPF Cohort												
pathway	lfc_IBD	lfc_PD	lfc_PDvsIBI	se_IBD	se_PD	se_PDvsIBI	pval_IBD	pval_PD	pval_PDvsI	adj_pval_IB	adj_pval_PI	adj_pval_PDvsIBD
METHGLYUT-PWY: superpathway of methylglyoxal degra	1.692688	-0.23607	-1.92876	0.514294	0.456241	0.487401	0.000997	0.604858	7.58E-05	0.007877	1	0.000675
GLYCOLYSIS-TCA-GLYOX-BYPASS: superpathway of gly	1.722109	-0.30828	-2.03039	0.572336	0.508254	0.525794	0.002622	0.544147	0.000113	0.020708	1	0.001002
PWY-7807: glyphosate degradation III	1.794567	0.076611	-1.71796	0.472904	0.419146	0.460539	0.000148	0.854972	0.000191	0.001315	1	0.00151
PWY-7409: phospholipid remodeling (phosphatidylethar	1.805401	0.295084	-1.51032	0.45064	0.399192	0.446291	6.17E-05	0.459784	0.000714	0.000549	1	0.005639
GLYCOL-GLYOXDEG-PWY: superpathway of glycol meta	1.8191	-0.61959	-2.43869	0.561534	0.498575	0.518591	0.001197	0.21397	2.57E-06	0.009457	1	2.29E-05
PWY-7805: (aminomethyl)phosphonate degradation	1.827317	0.171711	-1.65561	0.463879	0.411058	0.454746	8.18E-05	0.676146	0.000272	0.000727	1	0.002147
ECASYN-PWY: enterobacterial common antigen biosynt	1.83327	-0.45973	-2.293	0.511509	0.453746	0.48558	0.000338	0.310973	2.33E-06	0.002672	1	2.08E-05
PWY0-461: L-lysine degradation I	1.870458	0.065375	-1.80508	0.480422	0.425885	0.465383	9.89E-05	0.878001	0.000105	0.00088	1	0.00088
PWY-7269: mitochondrial NADPH production (yeast)	1.912939	-0.112	-2.02493	0.559451	0.496708	0.517205	0.000628	0.82161	9.03E-05	0.004958	1	0.000804
PWY-5837: 2-carboxy-1,4-naphthoquinol biosynthesis	1.92808	0.021483	-1.9066	0.681463	0.606027	0.599808	0.004665	0.971722	0.001479	0.036843	1	0.013165
SO4ASSIM-PWY: assimilatory sulfate reduction I	1.968545	0.027628	-1.94092	0.687785	0.61169	0.604156	0.004208	0.963975	0.001315	0.033234	1	0.011704
DARABCATK12-PWY: D-arabinose degradation I	1.995095	-0.01399	-2.00908	0.483424	0.428576	0.467322	3.68E-05	0.973963	1.71E-05	0.00029	1	0.000153
PWY1ZNC-1: assimilatory sulfate reduction IV	2.015787	0.335524	-1.68026	0.65324	0.580743	0.58047	0.00203	0.563433	0.003796	0.018062	1	0.029979
PWY-5747: 2-methylcitrate cycle II	2.027658	-0.20556	-2.23322	0.546045	0.484695	0.508307	0.000205	0.671494	1.12E-05	0.001615	1	9.93E-05
KDO-NAGLIPASYN-PWY: superpathway of (Kdo)2-lipid A	2.033566	-0.19375	-2.22731	0.502692	0.445844	0.479825	5.22E-05	0.663878	3.45E-06	0.000413	1	3.07E-05
PWY0-42: 2-methylcitrate cycle I	2.034024	-0.40602	-2.44004	0.66782	0.593805	0.590444	0.002321	0.49413	3.59E-05	0.018331	1	0.000319
P221-PWY: octane oxidation	2.04992	0.075564	-1.97436	0.554368	0.492153	0.513826	0.000218	0.877974	0.000122	0.001718	1	0.001084
PWY-7094: fatty acid salvage	2.051737	-0.40354	-2.45528	0.623067	0.55371	0.559938	0.000991	0.466127	1.16E-05	0.00783	1	0.000103
PWY-7204: pyridoxal 5'-phosphate salvage II (plants)	2.079721	0.360477	-1.71924	0.714027	0.635198	0.622266	0.003584	0.570372	0.005729	0.031888	1	0.045253
HOMOSER-METSYN-PWY: L-methionine biosynthesis I	2.092995	-0.34731	-2.44031	0.731434	0.650791	0.634331	0.004216	0.593566	0.00012	0.033303	1	0.001064
GALACTITOLCAT-PWY: galactitol degradation	2.094926	0.00421	-2.09072	0.699726	0.622388	0.612385	0.002754	0.994603	0.00064	0.021753	1	0.005695
PWY-7118: chitin deacetylation	2.114376	-0.47299	-2.58736	0.673333	0.598744	0.594225	0.001689	0.429549	1.34E-05	0.013337	1	0.000119
KETOGLUCONMET-PWY: ketogluconate metabolism	2.139616	0.229999	-1.90962	0.673311	0.598724	0.59421	0.001484	0.700869	0.00131	0.011723	1	0.01166
PWY-5855: ubiquinol-7 biosynthesis (early decarboxylat	2.154458	-0.02848	-2.18294	0.525265	0.466073	0.494597	4.1E-05	0.951279	1.02E-05	0.000324	1	9.05E-05
PWY-6803: phosphatidylcholine acyl editing	2.204471	-0.30518	-2.50965	0.78663	0.70023	0.67283	0.005072	0.662961	0.000191	0.04006	1	0.001704
PWY0-1337: oleate β-oxidation	2.248914	-0.2548	-2.50371	0.624634	0.555114	0.561001	0.000318	0.646231	8.08E-06	0.00251	1	7.19E-05
PWY0-166: superpathway of pyrimidine deoxyribonucle	2.276495	-0.22468	-2.50118	0.693363	0.616687	0.607997	0.001026	0.715605	3.89E-05	0.008105	1	0.000346
GLUCARDEG-PWY: D-glucarate degradation I	2.290937	-0.44246	-2.7334	0.684003	0.608303	0.601554	0.00081	0.467001	5.52E-06	0.006399	1	4.91E-05
PWY-5920: superpathway of heme b biosynthesis from g	2.294139	0.024869	-2.26927	0.74408	0.662118	0.643119	0.002048	0.970039	0.000418	0.016175	1	0.003718
PWY-5675: nitrate reduction V (assimilatory)	2.307861	-0.19429	-2.50215	0.757708	0.674325	0.652613	0.00232	0.773256	0.000126	0.018326	1	0.001122
PWY-6961: L-ascorbate degradation II (bacterial, aerobi	2.341562	-0.32957	-2.67114	0.669413	0.595232	0.591536	0.000469	0.579792	6.31E-06	0.003703	1	5.62E-05
PWY-5860: superpathway of demethylmenaquinol-6 bio	2.381059	-0.29201	-2.67307	0.693805	0.617084	0.608302	0.000599	0.63606	1.11E-05	0.004734	1	9.89E-05
GALACTARDEG-PWY: D-galactarate degradation I	2.381426	-0.31014	-2.69157	0.702929	0.625257	0.614595	0.000704	0.61988	1.19E-05	0.005564	1	0.000106
GLUCARGALACTSUPER-PWY: superpathway of D-glucar	2.381426	-0.31014	-2.69157	0.702929	0.625257	0.614595	0.000704	0.61988	1.19E-05	0.005564	1	0.000106
PWY-5850: superpathway of menaquinol-6 biosynthesis	2.443241	-0.31361	-2.75686	0.729594	0.649143	0.633053	0.000812	0.62901	1.33E-05	0.006411	1	0.000118
PWY-5896: superpathway of menaquinol-10 biosynthes	2.443241	-0.31361	-2.75686	0.729594	0.649143	0.633053	0.000812	0.62901	1.33E-05	0.006411	1	0.000118
PWY-5138: fatty acid β-oxidation IV (unsaturated, e	2.51919	-0.23589	-2.75508	0.697184	0.62011	0.610631	0.000302	0.703648	6.43E-06	0.002387	1	5.72E-05
PWY0-1338: polymyxin resistance	2.522506	-0.24346	-2.76596	0.62557	0.555953	0.561636	5.52E-05	0.661453	8.44E-07	0.000436	1	7.51E-06
PWY0-1415: superpathway of heme b biosynthesis from	2.533026	-0.20698	-2.74	0.736732	0.655536	0.63801	0.000586	0.752204	1.75E-05	0.004625	1	0.000156
PWY-5862: superpathway of demethylmenaquinol-9 bio	2.615212	0.302649	-2.31256	0.672447	0.59795	0.593617	0.000101	0.612756	9.79E-05	0.000871	1	0.000871
ARGDEG-PWY: superpathway of L-arginine, putrescine, a	2.658537	-0.15018	-2.80871	0.514034	0.456008	0.487231	2.32E-07	0.741909	8.18E-09	1.83E-06	1	7.28E-08
ORNARGDEG-PWY: superpathway of L-arginine and L-or	2.658537	-0.15018	-2.80871	0.514034	0.456008	0.487231	2.32E-07	0.741909	8.18E-09	1.83E-06	1	7.28E-08

IBD-Enriched Pathways in UFPF Cohort												
pathway	lfc_IBD	lfc_PD	lfc_PDvsIBI	se_IBD	se_PD	se_PDvsIBI	pval_IBD	pval_PD	pval_PDvsI	adj_pval_IB	adj_pval_PI	adj_pval_PDvsIBD
PWY-5845: superpathway of menaquinol-9 biosynthesis	2.701169	0.331825	-2.36934	0.705869	0.627891	0.616626	0.00013	0.597169	0.000122	0.001084	1	0.001084
PWY-7858: (5Z)-dodecenoate biosynthesis II	2.704787	-0.16747	-2.87225	0.697905	0.620756	0.611129	0.000106	0.787333	2.6E-06	0.00084	1	2.32E-05
PWY-5723: Rubisco shunt	2.718412	0.059937	-2.65848	0.845147	0.752639	0.714004	0.001298	0.936527	0.000197	0.010249	1	0.001749
FAO-PWY: fatty acid β-oxidation I (generic)	2.787142	-0.33828	-3.12542	0.80762	0.71903	0.68756	0.000558	0.638025	5.48E-06	0.00441	1	4.87E-05
PWY-6285: superpathway of fatty acids biosynthesis (E.	2.801724	-0.08568	-2.88741	0.778058	0.692553	0.666828	0.000317	0.901538	1.49E-05	0.002504	1	0.000133
AST-PWY: L-arginine degradation II (AST pathway)	2.921708	-0.24532	-3.16703	0.662172	0.588745	0.586576	1.02E-05	0.676912	6.69E-08	8.08E-05	1	5.96E-07
PWY0-1277: 3-phenylpropanoate and 3-(3-hydroxyphen	2.971232	0.005415	-2.96582	0.676148	0.601266	0.596157	1.11E-05	0.992815	6.53E-07	8.78E-05	1	5.81E-06
HCAMHPDEG-PWY: 3-phenylpropanoate and 3-(3-hydro	2.97847	0.163515	-2.81496	0.643734	0.572227	0.573985	3.71E-06	0.775068	9.38E-07	2.93E-05	1	8.35E-06
PWY-6690: cinnamate and 3-hydroxycinnamate degrada	2.97847	0.163515	-2.81496	0.643734	0.572227	0.573985	3.71E-06	0.775068	9.38E-07	2.93E-05	1	8.35E-06
PWY0-301: L-ascorbate degradation I (bacterial, anaero	3.020825	0.07418	-2.94664	0.632869	0.562493	0.566591	1.81E-06	0.895081	1.99E-07	1.43E-05	1	1.77E-06

Supplementary Table 5: Analysis of confounders on significant taxa in UFPF.

N= 54 PD, 26 IBD, and N= 16 control samples were examined. Nine variables, including Diagnosis and total read count per sample along with seven potential confounders, were included in a single model and tested simultaneously for association with bias corected relative abundances extracted from ANCOM-BC2 analysis of the 2 UFPF IBD associated species as identified from ANCOM-BC2 analysis. Multivariate linear regression was computed in R using the lm() funciton with BH multiple comparisons p-value adjustment. The variables included were sex (M vs F), Age (continuous variable, standardized using scale function in R), Diagnosis (PD, IBD, or Control), Reads aka total read count per sample (continuous variable, standardized using scale function in R), Indigestion_meds (Yes or No), Anti-TNF meds (Yes or No), Anti_inflammatories (Yes or No), Depression_anxiety_meds (Yes or No), and Iron_specific_supplement (Yes or No). Regression was performed with and without the influence of confounding variables, and the coefficients associated with the outcome variable, in this case association with IBD, were extracted. The ratio of coefficients with and without confounding variables were used to determine if confounders appeared to influence the findings (ratio < 1 or sign reversal (-)).

Taxa	Coefficient_Without_Cont	Coefficient_With_Con	Difference	Percent_Cha	Ratio	Adjustment moves coefficient towards null?
Klebsiella	5.983813307	5.407819358	0.575993949	9.62586765	**0.90374132**	**TRUE**
Faecalimonas	6.151747536	6.48050638	-0.328758844	-5.3441537	**1.05344154**	**FALSE**

Supplementary Table 6: Wallen PD demographics and metadata.

Full list of metadata associated with this project can be found on our GitHub [https://github.com/maevekrueger/UFPF_metagenomics] or from the original source where all Wallen PD data was accessed from [https://zenodo.org/record/7246185]

	Control (N=234)	PD (N=490)	p value
Age			< 0.001
Mean (SD)	65.825 (8.761)	68.659 (8.527)	
Range	35.000 - 88.000	23.000 - 90.000	
Sex			< 0.001
Female	164 (70.1%)	180 (36.7%)	
Male	70 (29.9%)	310 (63.3%)	
Constipation			< 0.001
N	199 (88.4%)	260 (55.6%)	
Y	26 (11.6%)	208 (44.4%)	
Diarrhea			0.033
N	195 (87.4%)	429 (92.5%)	
Y	28 (12.6%)	35 (7.5%)	
IBS			0.505
N	200 (89.7%)	428 (91.3%)	
Y	23 (10.3%)	41 (8.7%)	
IBD			0.636
N	217 (97.3%)	463 (97.9%)	
Y	6 (2.7%)	10 (2.1%)	
SIBO			0.488
N	224 (100.0%)	465 (99.8%)	
Y	0 (0.0%)	1 (0.2%)	
Celiac_disease			0.332
N	224 (100.0%)	475 (99.6%)	
Y	0 (0.0%)	2 (0.4%)	
Antibiotics_current			0.591
N	212 (93.4%)	441 (92.3%)	
Y	15 (6.6%)	37 (7.7%)	
Antibiotics_past_3_months			0.650
N	166 (73.8%)	355 (75.4%)	
Y	59 (26.2%)	116 (24.6%)	
Laxatives			< 0.001
N	201 (89.3%)	326 (68.6%)	
Y	24 (10.7%)	149 (31.4%)	
Indigestion_drugs			0.582
N	147 (65.6%)	299 (63.5%)	
Y	77 (34.4%)	172 (36.5%)	
Anti_inflammatory_drugs			0.317
N	155 (70.1%)	311 (66.3%)	

	Control (N=234)	PD (N=490)	p value
Y	66 (29.9%)	158 (33.7%)	
Radiation_Chemo			0.233
N	224 (100.0%)	472 (99.4%)	
Y	0 (0.0%)	3 (0.6%)	
Blood_thinners			0.152
N	180 (80.4%)	357 (75.5%)	
Y	44 (19.6%)	116 (24.5%)	
Cholesterol_med			0.829
N	127 (56.7%)	274 (57.6%)	
Y	97 (43.3%)	202 (42.4%)	
Blood_pressure_med			0.357
N	106 (47.1%)	242 (50.8%)	
Y	119 (52.9%)	234 (49.2%)	
Thyroid_med			0.373
N	180 (80.0%)	394 (82.8%)	
Y	45 (20.0%)	82 (17.2%)	
Asthma_or_COPD_med			0.899
N	206 (92.4%)	441 (92.6%)	
Y	17 (7.6%)	35 (7.4%)	
Diabetes_med			0.459
N	189 (84.4%)	409 (86.5%)	
Y	35 (15.6%)	64 (13.5%)	
Pain_med			0.03
N	188 (83.9%)	364 (76.8%)	
Y	36 (16.1%)	110 (23.2%)	
Depression_anxiety_mood_med			< 0.001
N	173 (77.2%)	297 (62.3%)	
Y	51 (22.8%)	180 (37.7%)	
Birth_control_or_estrogen			< 0.001
N	206 (91.6%)	456 (98.1%)	
Y	19 (8.4%)	9 (1.9%)	
Antihistamines			< 0.001
N	151 (67.4%)	397 (83.2%)	
Y	73 (32.6%)	80 (16.8%)	
Probiotic			0.011
N	182 (80.9%)	413 (88.1%)	
Y	43 (19.1%)	56 (11.9%)	
Co_Q_10			0.601
N	193 (85.8%)	416 (87.2%)	
Y	32 (14.2%)	61 (12.8%)	
Sleep_aid			< 0.001
N	165 (74.0%)	274 (58.3%)	
Y	58 (26.0%)	196 (41.7%)	

Supplementary Table 7: HMP2 IBD demographics and metadata.
Full list of metadata associated with this project can be found on our GitHub [https://github.com/maevekrueger/UFPF_metagenomics] or from the original source where all HMP2 data was accessed from [https://www.ibdmdb.org/results]

	nonIBD (N=139)	IBD (N=198)	p value
Age			0.77
Mean (SD)	54.367 (8.931)	54.025 (11.525)	
Range	40.000 - 69.000	41.000 - 76.000	
Sex			< 0.001
Female	25 (18.0%)	118 (59.6%)	
Male	114 (82.0%)	80 (40.4%)	
Diagnosis			< 0.001
CD	0 (0.0%)	101 (51.0%)	
UC	0 (0.0%)	97 (49.0%)	
non-IBD	139 (100.0%)	0 (0.0%)	
Site			< 0.001
Cedars-Sinai	20 (14.4%)	163 (82.3%)	
MGH	119 (85.6%)	35 (17.7%)	
Probiotic			< 0.001
N	130 (94.9%)	129 (65.8%)	
Y	7 (5.1%)	67 (34.2%)	
Immunosuppressants			< 0.001
N	139 (100.0%)	177 (89.4%)	
Y	0 (0.0%)	21 (10.6%)	
Antibiotics			< 0.001
N	139 (100.0%)	168 (84.8%)	
Y	0 (0.0%)	30 (15.2%)	
Chemotherapy			0.078
N	137 (98.6%)	188 (94.9%)	
Y	2 (1.4%)	10 (5.1%)	
Colonoscopy_past_2_weeks			0.207
N	131 (97.0%)	188 (98.9%)	
Y	4 (3.0%)	2 (1.1%)	
Diarrhea_past_2_weeks			< 0.001
N	129 (94.9%)	99 (52.1%)	
Y	7 (5.1%)	91 (47.9%)	
Bowel_surgery			< 0.001
N	136 (100.0%)	128 (68.8%)	
Y	0 (0.0%)	58 (31.2%)	

Supplementary Table 8: Wallen PD significant genera from ANCOM-BC2

Differential abundances of genera in PD (N = 490) vs Neurologically Healthy Control (N=234) from the Wallen et al. 2022 dataset (https://doi.org/10.1038/s41467-022-34667-x) were tested using ANCOM-BC2, while adjusting for covariates as described in their paper (stool sample collection method and total sequence count per sample). Shown are the significant genera that were detected by MetaPhlAn, however, only genera that were present in at least 10% of samples were included in ANCOM-BC2 analysis. LFC: indicates the log fold change compared to healthy control or in the case of lfc_PDvsIBD it is the log fold change of PD compared to IBD. SE: standard error; Pval: the uncorrected, two-sided P-value reported by ANCOM-BC2; Adj. Pval aka q-value from the false discovery rate, i.e., multiple-testing corrected significance q-value, calculated using the Benjamini-Hochberg method, alpha =0.05.

genus	lfc_PD	se_PD	pval_PD	adj_pval_PD
Enterococcus	1.44550219	0.42669316	0.00070485	0.01568292
Lactobacillus	2.0158137	0.50012583	5.5632E-05	0.00495121
Anaerostipes	-1.5086351	0.4437771	0.00067501	0.01568292
Fusicatenibacter	-1.6579696	0.51918078	0.00140591	0.02502528
Roseburia	-1.4924269	0.41117315	0.00028377	0.0126279

Supplementary Table 9: HMP2 IBD significant genera from ANCOM-BC2

Differential abundances of genera in IBD (N = 198) vs non-IBD (N=139) from the Human Microbiome Project 2 dataset (downloaded from https://www.ibdmdb.org/downloads/html/products_MGX_2017-08-12.html) were tested using ANCOM-BC2, while adjusting for covariates (diagnosis and total sequence count per sample). Shown are the significant genera that were detected by MetaPhlAn, however, only genera that were present in at least 10% of samples were included in ANCOM-BC2 analysis. LFC: indicates the log fold change compared to healthy control or in the case of lfc_PDvsIBD it is the log fold change of PD compared to IBD. SE: standard error; Pval: the uncorrected, two-sided P-value reported by ANCOM-BC2; Adj. Pval aka q-value from the false discovery rate, i.e., multiple-testing corrected significance q-value, calculated using the Benjamini-Hochberg method, alpha =0.05.

genus	lfc_IBD	se_IBD	pval_IBD	adj_pval_IBD
Propionibacteriaceae_unclass	1.386239909	0.6104943	0.02316609	0.04633217
Eggerthella	2.524736517	0.55936445	6.3742E-06	4.1432E-05
Bacteroides	1.607102252	0.512353	0.00170856	0.00492062
Barnesiella	-2.335730755	0.80769275	0.00382974	0.00929497
Paraprevotella	1.497886236	0.56005263	0.00748306	0.01691823
Alistipes	-2.367287547	0.69885498	0.00070564	0.00229331
Clostridiaceae_noname	2.510413358	0.50445864	6.4761E-07	4.8108E-06
Clostridium	4.982193319	0.64862156	1.576E-14	8.1953E-13
Clostridiales_noname	3.60287205	0.62037957	6.3402E-09	6.5938E-08
Flavonifractor	4.685476016	0.6637621	1.6774E-12	4.3613E-11
Pseudoflavonifractor	2.087114247	0.57137208	0.00025939	0.00096343
Anaerostipes	2.110060724	0.64088449	0.00099329	0.00303831
Blautia	1.905720406	0.50601272	0.00016578	0.00066313
Lachnospiraceae_noname	2.064972255	0.48431349	2.0106E-05	0.00010455
Oscillibacter	2.097038079	0.61454886	0.00064412	0.00223296
Faecalibacterium	-1.745661878	0.69606514	0.0121451	0.02631438
Ruminococcaceae_noname	2.280135077	0.58469504	9.6312E-05	0.00041735
Coprobacillus	3.532828945	0.53303676	3.4087E-11	5.9083E-10
Holdemania	1.924590438	0.65671097	0.00338253	0.00879458
Acidaminococcaceae_unclass	2.234501741	0.5709828	9.0994E-05	0.00041735
Acidaminococcus	4.352905756	0.7453279	5.2121E-09	6.5938E-08
Dialister	2.238027251	0.77614229	0.00393249	0.00929497
Burkholderiales_noname	3.048018436	0.70847898	1.6911E-05	9.7708E-05
Parasutterella	1.640081121	0.67109838	0.01453043	0.03022329
Escherichia	3.999681669	0.76775943	1.893E-07	1.6406E-06
Klebsiella	1.898276212	0.60808473	0.00179792	0.00492062

Supplementary Table 10: Wallen PD signficantly depleted pathways from ANCOM-BC2

Differential abundances of MetaCyc pathways in PD (N = 490) vs Neurologically Healthy Control (N=234) from the Wallen et al. 2022 dataset (https://doi.org/10.1038/s41467-022-34667-x) were tested using ANCOM-BC2, while adjusting for covariates as described in their paper (stool sample collection method and total sequence count per sample). Shown are the significantly depleted pathways that were detected by HUMAnN, however, only pathways that were present in at least 25% of samples were included in ANCOM-BC2 analysis. LFC: indicates the log fold change compared to healthy control or in the case of lfc_PDvsIBD it is the log fold change of PD compared to IBD. SE: standard error; Pval: the uncorrected, two-sided P-value reported by ANCOM-BC2; Adj. Pval aka q-value from the false discovery rate, i.e., multiple-testing corrected significance q-value, calculated using the Benjamini-Hochberg method, alpha =0.05.

pathway	lfc_PD	se_PD	pval_PD	adj_pval_PD
PWY-5505: L-glutamate and L-glutamine biosynthesis	-0.48359	0.043938	3.56656E-28	1.2697E-25
PWY-6863: pyruvate fermentation to hexanol (engineered)	-0.6692	0.072817	3.92412E-20	6.9849E-18
METH-ACETATE-PWY: methanogenesis from acetate	-0.69132	0.077487	4.59242E-19	5.4497E-17
PWY-5121: superpathway of geranylgeranyl diphosphate bios	-0.33097	0.04521	2.46669E-13	2.1954E-11
SALVADEHYPOX-PWY: adenosine nucleotides degradation II	-0.32702	0.045506	6.65817E-13	4.7406E-11
PWY-6527: stachyose degradation	-0.41508	0.059162	2.28347E-12	1.3549E-10
PWY66-422	-0.40258	0.057644	2.87244E-12	1.4608E-10
PWY-7323: superpathway of GDP-mannose-derived O-antige	-0.43304	0.063536	9.37618E-12	4.1724E-10
PWY-6317: D-galactose degradation I (Leloir pathway)	-0.38296	0.056759	1.50782E-11	5.9643E-10
PWY-6168: flavin biosynthesis III (fungi)	-0.36189	0.054955	4.54195E-11	1.6169E-09
PWY0-1319: CDP-diacylglycerol biosynthesis II	-0.34575	0.053611	1.12327E-10	3.3403E-09
PWY-5667: CDP-diacylglycerol biosynthesis I	-0.34573	0.05361	1.12595E-10	3.3403E-09
FASYN-INITIAL-PWY: superpathway of fatty acid biosynthesis	-0.3387	0.053349	2.16981E-10	5.9419E-09
PWY-7357: thiamine phosphate formation from pyrithiamine	-0.34266	0.054233	2.64665E-10	6.73E-09
COBALSYN-PWY: superpathway of adenosylcobalamin salva	-0.37034	0.059211	3.98757E-10	9.4638E-09
RIBOSYN2-PWY: flavin biosynthesis I (bacteria and plants)	-0.33558	0.053753	4.2909E-10	9.5473E-09
PWY-7237: myo-, chiro- and scyllo-inositol degradation	-0.51957	0.083416	4.70358E-10	9.8499E-09
PWY-6305: superpathway of putrescine biosynthesis	-0.42735	0.069741	8.91917E-10	1.6828E-08
PWY-7242: D-fructuronate degradation	-0.42541	0.069437	8.98124E-10	1.6828E-08
GLUCUROCAT-PWY: superpathway of β-D-glucuronosid	-0.40823	0.066789	9.82309E-10	1.7485E-08
THISYNARA-PWY: superpathway of thiamine diphosphate bio	-0.35346	0.057966	1.07547E-09	1.7568E-08
GLYCOGENSYNTH-PWY: glycogen biosynthesis I (from ADP-D	-0.34797	0.05708	1.08567E-09	1.7568E-08
PANTOSYN-PWY: superpathway of coenzyme A biosynthesis	-0.31701	0.052733	1.83603E-09	2.8419E-08
PWY-6897: thiamine diphosphate salvage II	-0.32669	0.054446	1.97044E-09	2.9228E-08
PWY-5659: GDP-mannose biosynthesis	-0.35502	0.059513	2.43931E-09	3.4736E-08
PANTO-PWY: phosphopantothenate biosynthesis I	-0.31706	0.053638	3.39971E-09	4.655E-08
TEICHOICACID-PWY: poly(glycerol phosphate) wall teichoic a	-0.39885	0.067826	4.09084E-09	5.3939E-08
PWY-7456: β-(1,4)-mannan degradation	-0.43044	0.073783	5.41782E-09	6.8884E-08
PWY0-1296: purine ribonucleosides degradation	-0.32234	0.055771	7.48097E-09	9.1835E-08
GLUTORN-PWY: L-ornithine biosynthesis I	-0.3137	0.054558	8.93755E-09	1.0606E-07
PWY-1042: glycolysis IV	-0.29756	0.052171	1.17355E-08	1.3477E-07
GALACT-GLUCUROCAT-PWY: superpathway of hexuronide ar	-0.39838	0.070417	1.53715E-08	1.7101E-07
PWY-5154: L-arginine biosynthesis III (via N-acetyl-L-citrullin	-0.35385	0.062616	1.59419E-08	1.7198E-07
PWY-6163: chorismate biosynthesis from 3-dehydroquinate	-0.29236	0.052126	2.03913E-08	2.1351E-07
PWY-6609: adenine and adenosine salvage III	-0.29067	0.052034	2.32138E-08	2.3612E-07
PWY-6700: queuosine biosynthesis I (de novo)	-0.29051	0.052717	3.57454E-08	3.5348E-07
PWY-6737: starch degradation V	-0.29141	0.053123	4.12186E-08	3.9659E-07
GALACTUROCAT-PWY: D-galacturonate degradation I	-0.355	0.065544	6.08781E-08	5.7033E-07
ASPASN-PWY: superpathway of L-aspartate and L-asparagine	-0.30915	0.05722	6.55808E-08	5.9863E-07
COMPLETE-ARO-PWY: superpathway of aromatic amino acid	-0.28087	0.05228	7.77039E-08	6.9157E-07
ILEUSYN-PWY: L-isoleucine biosynthesis I (from threonine)	-0.28166	0.052519	8.18148E-08	6.9348E-07
VALSYN-PWY: L-valine biosynthesis	-0.28166	0.052519	8.18148E-08	6.9348E-07
GLCMANNANAUT-PWY: superpathway of N-acetylglucosami	-0.32322	0.06045	8.95049E-08	7.2658E-07
PWY-7111: pyruvate fermentation to isobutanol (engineered)	-0.28162	0.052675	8.98016E-08	7.2658E-07
CALVIN-PWY: Calvin-Benson-Bassham cycle	-0.28289	0.052958	9.20141E-08	7.2793E-07
PWY-4242	-0.27952	0.052426	9.73121E-08	7.5311E-07

pathway	lfc_PD	se_PD	pval_PD	adj_pval_PD
ARO-PWY: chorismate biosynthesis I	-0.27773	0.052381	1.14423E-07	8.667E-07
TRPSYN-PWY: L-tryptophan biosynthesis	-0.29497	0.055696	1.18328E-07	8.776E-07
NONMEVIPP-PWY: methylerythritol phosphate pathway I	-0.27219	0.051669	1.37867E-07	1.0016E-06
COA-PWY: coenzyme A biosynthesis I (prokaryotic)	-0.27386	0.052272	1.61232E-07	1.1255E-06
PWY-5097: L-lysine biosynthesis VI	-0.27139	0.051787	1.6022E-07	1.1255E-06
RHAMCAT-PWY: L-rhamnose degradation I	-0.31224	0.060033	1.98088E-07	1.3306E-06
PWY-5695: inosine 5'-phosphate degradation	-0.27598	0.053028	1.94649E-07	1.3306E-06
PWY-7219: adenosine ribonucleotides de novo biosynthesis	-0.26838	0.051881	2.3048E-07	1.5195E-06
PWY-6387: UDP-N-acetylmuramoyl-pentapeptide biosynthes	-0.26705	0.051703	2.40221E-07	1.5549E-06
PYRIDNUCSYN-PWY: NAD de novo biosynthesis I (from aspar	-0.27064	0.052539	2.58719E-07	1.6447E-06
PWY-5188: tetrapyrrole biosynthesis I (from glutamate)	-0.31542	0.061306	2.67535E-07	1.6709E-06
PWY-6386: UDP-N-acetylmuramoyl-pentapeptide biosynthes	-0.26623	0.0518	2.75301E-07	1.6898E-06
DTDPRHAMSYN-PWY: dTDP-β-L-rhamnose biosynthesis	-0.27196	0.052994	2.86729E-07	1.7301E-06
PWY-6121: 5-aminoimidazole ribonucleotide biosynthesis I	-0.26551	0.051842	3.03168E-07	1.7938E-06
PWY-6385: peptidoglycan biosynthesis III (mycobacteria)	-0.26497	0.051764	3.07367E-07	1.7938E-06
PWY-5686: UMP biosynthesis I	-0.26435	0.051744	3.24091E-07	1.8609E-06
PWY-6122: 5-aminoimidazole ribonucleotide biosynthesis II	-0.26462	0.05199	3.58266E-07	1.9929E-06
PWY-6277: superpathway of 5-aminoimidazole ribonucleotid	-0.26462	0.05199	3.58266E-07	1.9929E-06
PWY-7221: guanosine ribonucleotides de novo biosynthesis	-0.26121	0.051533	4.00541E-07	2.1937E-06
PEPTIDOGLYCANSYN-PWY: peptidoglycan biosynthesis I (me	-0.26116	0.051671	4.31958E-07	2.33E-06
PWY-6507: 4-deoxy-L-threo-hex-4-enopyranuronate degrada	-0.34376	0.068093	4.45684E-07	2.3681E-06
PWY-6151: S-adenosyl-L-methionine salvage I	-0.26983	0.053798	5.28577E-07	2.7271E-06
1CMET2-PWY: folate transformations III (E. coli)	-0.25972	0.051765	5.24222E-07	2.7271E-06
BRANCHED-CHAIN-AA-SYN-PWY: superpathway of branched	-0.26489	0.052875	5.44771E-07	2.7706E-06
PWY0-1586: peptidoglycan maturation (meso-diaminopimela	-0.29955	0.059886	5.6745E-07	2.8452E-06
PWY-724: superpathway of L-lysine, L-threonine and L-methi	-0.26022	0.052109	5.92014E-07	2.9272E-06
PWY-7199: pyrimidine deoxyribonucleosides salvage	-0.26737	0.053588	6.05875E-07	2.9547E-06
ARGSYNBSUB-PWY: L-arginine biosynthesis II (acetyl cycle)	-0.26995	0.054317	6.69964E-07	3.2231E-06
PWY-7400: L-arginine biosynthesis IV (archaebacteria)	-0.26726	0.053986	7.40325E-07	3.5141E-06
ARGSYN-PWY: L-arginine biosynthesis I (via L-ornithine)	-0.26689	0.054027	7.81315E-07	3.6598E-06
PWY-6608: guanosine nucleotides degradation III	-0.32567	0.066258	8.86721E-07	4.0511E-06
HSERMETANA-PWY: L-methionine biosynthesis III	-0.26788	0.054501	8.87594E-07	4.0511E-06
PWY-5103: L-isoleucine biosynthesis III	-0.26055	0.053268	1.00167E-06	4.5138E-06
PWY-5973: cis-vaccenate biosynthesis	-0.285	0.059227	1.49497E-06	6.6526E-06
PWY-2942: L-lysine biosynthesis III	-0.25022	0.052191	1.63157E-06	7.1708E-06
TRNA-CHARGING-PWY: tRNA charging	-0.24706	0.051629	1.70756E-06	7.4133E-06
PHOSLIPSYN-PWY: superpathway of phospholipid biosynthe	-0.32032	0.066992	1.73981E-06	7.4623E-06
PWY-6270: isoprene biosynthesis I	-0.26402	0.055314	1.81496E-06	7.692E-06
COA-PWY-1: superpathway of coenzyme A biosynthesis III (m	-0.24742	0.052052	2.00131E-06	8.3819E-06
PWY-7560: methylerythritol phosphate pathway II	-0.26549	0.056062	2.18302E-06	9.0367E-06
PWY-6353: purine nucleotides degradation II (aerobic)	-0.32985	0.070415	2.80744E-06	1.1481E-05
UNINTEGRATED	-0.25185	0.053788	2.83806E-06	1.1481E-05
PWY490-3: nitrate reduction VI (assimilatory)	-0.54285	0.116107	2.93289E-06	1.1732E-05
PWY-7539: 6-hydroxymethyl-dihydropterin diphosphate bios	-0.32447	0.069735	3.27185E-06	1.28E-05
PWY-3001: superpathway of L-isoleucine biosynthesis I	-0.24752	0.053178	3.2473E-06	1.28E-05
PWY-6123: inosine-5'-phosphate biosynthesis I	-0.24265	0.052548	3.88164E-06	1.502E-05
PWY4FS-7: phosphatidylglycerol biosynthesis I (plastidic)	-0.3236	0.070312	4.17833E-06	1.5824E-05
PWY4FS-8: phosphatidylglycerol biosynthesis II (non-plastidi	-0.32359	0.070311	4.17828E-06	1.5824E-05
NONOXIPENT-PWY: pentose phosphate pathway (non-oxidat	-0.2611	0.057215	5.02983E-06	1.8711E-05
HISTSYN-PWY: L-histidine biosynthesis	-0.23914	0.052409	5.04558E-06	1.8711E-05
PWY-3841: folate transformations II (plants)	-0.23312	0.051598	6.24376E-06	2.2915E-05
PWY-6124: inosine-5'-phosphate biosynthesis II	-0.23774	0.052782	6.66072E-06	2.4196E-05
THRESYN-PWY: superpathway of L-threonine biosynthesis	-0.24101	0.053803	7.48565E-06	2.6918E-05
COLANSYN-PWY: colanic acid building blocks biosynthesis	-0.26212	0.058605	7.72707E-06	2.7508E-05
PWY-7663: gondoate biosynthesis (anaerobic)	-0.26885	0.060645	9.28569E-06	3.273E-05

pathway	lfc_PD	se_PD	pval_PD	adj_pval_PD
PWY-6703: preQ0 biosynthesis	-0.25511	0.057885	1.04728E-05	3.6552E-05
PWY-6147: 6-hydroxymethyl-dihydropterin diphosphate bios	-0.30689	0.069914	1.13606E-05	3.9266E-05
PWY-5304: superpathway of sulfur oxidation (Acidianus amb	-0.77182	0.178712	1.56901E-05	5.3709E-05
ANAGLYCOLYSIS-PWY: glycolysis III (from glucose)	-0.22784	0.052999	1.71526E-05	5.8155E-05
POLYISOPRENSYN-PWY: polyisoprenoid biosynthesis (E. coli	-0.226	0.052628	1.75331E-05	5.8885E-05
PWY-5676: acetyl-CoA fermentation to butanoate II	-0.29927	0.071867	3.12456E-05	0.00010396
PWY-5100: pyruvate fermentation to acetate and lactate II	-0.23385	0.057603	4.91406E-05	0.0001605
SER-GLYSYN-PWY: superpathway of L-serine and glycine bios	-0.21743	0.053556	4.91114E-05	0.0001605
PWY-6606: guanosine nucleotides degradation II	-0.29096	0.072241	5.63456E-05	0.00018235
PWY-5104: L-isoleucine biosynthesis IV	-0.28291	0.072693	9.94929E-05	0.00031909
PWY-6470: peptidoglycan biosynthesis V (β-lactam resi	-0.29548	0.077354	0.000133509	0.00042061
CITRULBIO-PWY: L-citrulline biosynthesis	-0.17037	0.044576	0.000132371	0.00042061
PWY-841: superpathway of purine nucleotides de novo biosy	-0.21202	0.055773	0.000143821	0.00044913
PWY-4984: urea cycle	-0.1695	0.045516	0.000196148	0.00060721
PWY-5005: biotin biosynthesis II	-0.45009	0.124227	0.000291088	0.0008782
GOLPDLCAT-PWY: superpathway of glycerol degradation to 1	-0.26755	0.07383	0.000290163	0.0008782
PWY-7208: superpathway of pyrimidine nucleobases salvage	-0.2107	0.058538	0.000319017	0.00095437
PWY-5030: L-histidine degradation III	-0.26229	0.073915	0.000387387	0.00114259
PWY-7229: superpathway of adenosine nucleotides de novo	-0.19994	0.056356	0.000388352	0.00114259
PWY-5347: superpathway of L-methionine biosynthesis (tran	-0.14811	0.041941	0.000413618	0.00120695
ANAEROFRUCAT-PWY: homolactic fermentation	-0.19105	0.055274	0.000547554	0.00157201
ARGININE-SYN4-PWY: L-ornithine biosynthesis II	-0.23259	0.068592	0.000696689	0.00196842
HISDEG-PWY: L-histidine degradation I	-0.21172	0.062905	0.000763554	0.00214036
PWY-6126: superpathway of adenosine nucleotides de novo	-0.19645	0.058471	0.000779764	0.00216872
PWY-7282: 4-amino-2-methyl-5-diphosphomethylpyrimidine	-0.20731	0.061826	0.000799039	0.0022051
PWY-1269: CMP-3-deoxy-D-manno-octulosonate biosynthes	-0.2499	0.075516	0.000935625	0.00254262
PWY-7228: superpathway of guanosine nucleotides de novo	-0.19453	0.058962	0.000969279	0.00261412
P185-PWY: formaldehyde assimilation III (dihydroxyacetone	-0.27323	0.08367	0.001092537	0.00288106
PWY-6125: superpathway of guanosine nucleotides de novo	-0.19345	0.059993	0.001261494	0.00330215
PWY-6471: peptidoglycan biosynthesis IV (Enterococcus fae	-0.22083	0.068722	0.001311904	0.00340903
UNMAPPED	-0.17189	0.053597	0.001340746	0.00345874
PWY-6892: thiazole component of thiamine diphosphate bios	-0.37914	0.120109	0.001595905	0.00405816
THISYN-PWY: superpathway of thiamine diphosphate biosynt	-0.38263	0.12143	0.00162689	0.00410761
MET-SAM-PWY: superpathway of S-adenosyl-L-methionine bi	-0.13744	0.04387	0.001731015	0.00433973
GLYCOLYSIS-E-D: superpathway of glycolysis and the Entner	-0.20808	0.069195	0.002637228	0.0064305
P164-PWY: purine nucleobases degradation I (anaerobic)	-0.20206	0.067419	0.002725614	0.00660081
FUCCAT-PWY: fucose degradation	-0.14186	0.047882	0.003050708	0.00724035
METSYN-PWY: superpathway of L-homoserine and L-methion	-0.13509	0.045903	0.003251646	0.00766613
POLYAMSYN-PWY: superpathway of polyamine biosynthesis	-0.25849	0.088222	0.0033897	0.00788714
PWY-7220: adenosine deoxyribonucleotides de novo biosynt	-0.17914	0.062266	0.004015566	0.00910536
PWY-7222: guanosine deoxyribonucleotides de novo biosynt	-0.17914	0.062266	0.004015566	0.00910536
GLUCONEO-PWY: gluconeogenesis I	-0.1672	0.058327	0.004149482	0.0092326
ARG+POLYAMINE-SYN: superpathway of arginine and polyam	-0.2417	0.084999	0.004461684	0.00980469
PYRIDOXSYN-PWY: pyridoxal 5'-phosphate biosynthesis I	-0.11858	0.042534	0.005306671	0.01151936
PWY-6936: seleno-amino acid biosynthesis (plants)	-0.1878	0.068498	0.006111901	0.01310745
PWY-7197: pyrimidine deoxyribonucleotide phosphorylation	-0.15819	0.059721	0.008075618	0.01671465
PENTOSE-P-PWY: pentose phosphate pathway	-0.16134	0.062006	0.009266264	0.01874312
OANTIGEN-PWY: O-antigen building blocks biosynthesis (E. c	-0.14447	0.058941	0.014240968	0.02785596
PWY0-1261: anhydromuropeptides recycling I	-0.11077	0.046255	0.016631096	0.03235339
HEXITOLDEGSUPER-PWY: superpathway of hexitol degradati	-0.15487	0.066679	0.020203147	0.03746
PWY0-162: superpathway of pyrimidine ribonucleotides de no	-0.13591	0.060136	0.023824331	0.0439454

Supplementary Table 11: HMP2 IBD significantly depleted pathways from ANCOM-BC2

Differential abundances of MetaCyc pathways in IBD (N = 198) vs non-IBD (N=139) from the Human Microbiome Project 2 dataset (downloaded from https://www.ibdmdb.org/downloads/html/products_MGX_2017-08-12.html) were tested using ANCOM-BC2, while adjusting for covariates (age, diagnosis, and total sequence count per sample). Shown are the significantly depleted pathways that were detected by HUMAnN, however, only pathways that were present in at least 10% of samples were included in ANCOM-BC2 analysis. LFC: indicates the log fold change compared to healthy control or in the case of lfc_PDvsIBD it is the log fold change of PD compared to IBD. SE: standard error; Pval: the uncorrected, two-sided P-value reported by ANCOM-BC2; Adj. Pval aka q-value from the false discovery rate, i.e., multiple-testing corrected significance q-value, calculated using the Benjamini-Hochberg method, alpha =0.05.

pathway	lfc_IBD	se_IBD	pval_IBD	adj_pval_IBD
ARO-PWY: chorismate biosynthesis I	-0.29148	0.122171	0.017041	0.032766
BRANCHED-CHAIN-AA-SYN-PWY: superpathway of branched ami	-0.65595	0.106345	6.91E-10	2.90E-08
CALVIN-PWY: Calvin-Benson-Bassham cycle	-0.32084	0.119924	0.007465	0.016137
CENTFERM-PWY: pyruvate fermentation to butanoate	-0.85652	0.187697	5.04E-06	3.52E-05
COBALSYN-PWY: adenosylcobalamin salvage from cobinamide I	-0.8578	0.164225	1.76E-07	2.25E-06
COMPLETE-ARO-PWY: superpathway of aromatic amino acid bios	-0.26981	0.120141	0.024718	0.045137
DAPLYSINESYN-PWY: L-lysine biosynthesis I	-0.95317	0.174156	4.42E-08	8.67E-07
DENOVOPURINE2-PWY: superpathway of purine nucleotides de n	-0.69717	0.133262	1.68E-07	2.25E-06
GALACT-GLUCUROCAT-PWY: superpathway of hexuronide and he	-0.87943	0.18829	3.00E-06	2.39E-05
GALACTUROCAT-PWY: D-galacturonate degradation I	-0.8727	0.178319	9.88E-07	1.04E-05
GLUCONEO-PWY: gluconeogenesis I	-1.40898	0.267139	1.33E-07	1.87E-06
GLUCUROCAT-PWY: superpathway of β-D-glucuronide and I	-0.74039	0.175188	2.38E-05	0.00012
GLYCOGENSYNTH-PWY: glycogen biosynthesis I (from ADP-D-Glu	-0.66634	0.125074	9.96E-08	1.54E-06
HEMESYN2-PWY: heme biosynthesis II (anaerobic)	-0.49863	0.217206	0.021695	0.040625
HOMOSER-METSYN-PWY: L-methionine biosynthesis I	-0.91622	0.172744	1.13E-07	1.67E-06
HSERMETANA-PWY: L-methionine biosynthesis III	-1.27925	0.144284	7.57E-19	2.22E-16
MET-SAM-PWY: superpathway of S-adenosyl-L-methionine biosyn	-0.91905	0.17217	9.40E-08	1.54E-06
METSYN-PWY: L-homoserine and L-methionine biosynthesis	-0.95731	0.16778	1.16E-08	2.84E-07
NONOXIPENT-PWY: pentose phosphate pathway (non-oxidative b	-0.28291	0.123575	0.022057	0.041043
OANTIGEN-PWY: O-antigen building blocks biosynthesis (E. coli)	-0.75381	0.195226	0.000113	0.000377
P108-PWY: pyruvate fermentation to propanoate I	-1.02684	0.228096	6.74E-06	4.50E-05
P162-PWY: L-glutamate degradation V (via hydroxyglutarate)	-0.56084	0.225589	0.012914	0.025829
P4-PWY: superpathway of L-lysine, L-threonine and L-methionine	-0.70187	0.22	0.001421	0.003482
POLYAMINSYN3-PWY: superpathway of polyamine biosynthesis II	-0.43317	0.182476	0.017604	0.033391
POLYAMSYN-PWY: superpathway of polyamine biosynthesis I	-0.38197	0.167875	0.022888	0.042056
PRPP-PWY: superpathway of histidine, purine, and pyrimidine bios	-1.13698	0.234849	1.29E-06	1.26E-05
PWY-3001: superpathway of L-isoleucine biosynthesis I	-0.37103	0.129881	0.004281	0.00991
PWY-5030: L-histidine degradation III	-0.90291	0.182239	7.25E-07	8.20E-06
PWY-5100: pyruvate fermentation to acetate and lactate II	-0.57761	0.107856	8.54E-08	1.54E-06
PWY-5103: L-isoleucine biosynthesis III	-0.64729	0.114225	1.45E-08	3.29E-07
PWY-5104: L-isoleucine biosynthesis IV	-0.8076	0.177297	5.24E-06	3.58E-05
PWY-5177: glutaryl-CoA degradation	-1.26424	0.204318	6.11E-10	2.90E-08
PWY-5188: tetrapyrrole biosynthesis I (from glutamate)	-0.33798	0.125421	0.007044	0.01557
PWY-5345: superpathway of L-methionine biosynthesis (by sulfhy	-1.38734	0.228617	1.29E-09	4.75E-08
PWY-5347: superpathway of L-methionine biosynthesis (transsulf	-0.87051	0.163216	9.64E-08	1.54E-06
PWY-5367: petroselinate biosynthesis	-0.94457	0.212399	8.70E-06	5.44E-05
PWY-5676: acetyl-CoA fermentation to butanoate II	-0.67808	0.25541	0.007934	0.016942
PWY-5690: TCA cycle II (plants and fungi)	-0.89279	0.219612	4.80E-05	0.000191
PWY-6121: 5-aminoimidazole ribonucleotide biosynthesis I	-0.39672	0.121371	0.001081	0.002812

pathway	lfc_IBD	se_IBD	pval_IBD	adj_pval_IBD
PWY-6122: 5-aminoimidazole ribonucleotide biosynthesis II	-0.39753	0.12382	0.001325	0.003273
PWY-621: sucrose degradation III (sucrose invertase)	-0.67967	0.142085	1.72E-06	1.53E-05
PWY-6277: superpathway of 5-aminoimidazole ribonucleotide bio	-0.39753	0.12382	0.001325	0.003273
PWY-6305: putrescine biosynthesis IV	-0.29797	0.125102	0.017228	0.032889
PWY-6317: galactose degradation I (Leloir pathway)	-0.60451	0.096564	3.84E-10	2.56E-08
PWY-6507: 4-deoxy-L-threo-hex-4-enopyranuronate degradation	-0.87359	0.199124	1.15E-05	6.89E-05
PWY-6527: stachyose degradation	-0.58024	0.092017	2.87E-10	2.56E-08
PWY-6545: pyrimidine deoxyribonucleotides de novo biosynthesis	-0.29495	0.079209	0.000196	0.000591
PWY-6590: superpathway of Clostridium acetobutylicum acidoge	-0.89222	0.199817	8.00E-06	5.11E-05
PWY-6595: superpathway of guanosine nucleotides degradation (	-0.40745	0.166594	0.014454	0.02852
PWY-6606: guanosine nucleotides degradation II	-0.7247	0.145599	6.45E-07	7.58E-06
PWY-6737: starch degradation V	-0.4676	0.134439	0.000505	0.001387
PWY-6936: seleno-amino acid biosynthesis	-0.68466	0.161359	2.20E-05	0.000114
PWY-6969: TCA cycle V (2-oxoglutarate:ferredoxin oxidoreductase	-0.98746	0.235412	2.73E-05	0.000132
PWY-7187: pyrimidine deoxyribonucleotides de novo biosynthesis	-0.37541	0.133957	0.005072	0.01165
PWY-7196: superpathway of pyrimidine ribonucleosides salvage	-1.31984	0.220523	2.16E-09	6.36E-08
PWY-7199: pyrimidine deoxyribonucleosides salvage	-0.43523	0.126335	0.000571	0.00154
PWY-7211: superpathway of pyrimidine deoxyribonucleotides de r	-0.74353	0.185669	6.21E-05	0.000243
PWY-7234: inosine-5'-phosphate biosynthesis III	-1.17698	0.18826	4.06E-10	2.56E-08
PWY-7242: D-fructuronate degradation	-0.6141	0.170857	0.000325	0.000947
PWY-7357: thiamin formation from pyrithiamine and oxythiamine	-0.3579	0.13302	0.007133	0.01565
PWY-7383: anaerobic energy metabolism (invertebrates, cytosol)	-0.55289	0.16117	0.000602	0.001604
PWY-7456: mannan degradation	-1.70361	0.284318	2.07E-09	6.36E-08
PWY0-1298: superpathway of pyrimidine deoxyribonucleosides de	-0.4967	0.17966	0.005698	0.012986
PWY0-781: aspartate superpathway	-0.82591	0.220523	0.00018	0.000552
PWY4LZ-257: superpathway of fermentation (Chlamydomonas re	-0.49055	0.178714	0.006053	0.013481
PWY66-422: D-galactose degradation V (Leloir pathway)	-0.57124	0.091532	4.35E-10	2.56E-08
PYRIDNUCSYN-PWY: NAD biosynthesis I (from aspartate)	-0.62871	0.114629	4.14E-08	8.67E-07
SER-GLYSYN-PWY: superpathway of L-serine and glycine biosynth	-0.52327	0.121416	1.63E-05	9.10E-05
SO4ASSIM-PWY: sulfate reduction I (assimilatory)	-0.53457	0.221454	0.015782	0.030727
TCA: TCA cycle I (prokaryotic)	-1.00762	0.235507	1.88E-05	0.000101
TRNA-CHARGING-PWY: tRNA charging	-0.43814	0.097426	6.89E-06	4.50E-05
TRPSYN-PWY: L-tryptophan biosynthesis	-0.39722	0.156557	0.011173	0.0225
UDPNAGSYN-PWY: UDP-N-acetyl-D-glucosamine biosynthesis I	-0.62649	0.210158	0.002873	0.006811

Supplementary Table 12: Wallen PD significantly enriched pathways from ANCOM-BC2

Differential abundances of MetaCyc pathways in PD (N = 490) vs Neurologically Healthy Control (N=234) from the Wallen et al. 2022 dataset (https://doi.org/10.1038/s41467-022-34667-x) were tested using ANCOM-BC2, while adjusting for covariates as described in their paper (stool sample collection method and total sequence count per sample). Shown are the significantly enriched pathways that were detected by HUMAnN, however, only pathways that were present in at least 25% of samples were included in ANCOM-BC2 analysis. LFC: indicates the log fold change compared to healthy control or in the case of lfc_PDvsIBD it is the log fold change of PD compared to IBD. SE: standard error; Pval: the uncorrected, two-sided P-value reported by ANCOM-BC2; Adj. Pval aka q-value from the false discovery rate, i.e., multiple-testing corrected significance q-value, calculated using the Benjamini-Hochberg method, alpha =0.05.

pathway	lfc_PD	se_PD	pval_PD	adj_pval_PD
PWY66-389: phytol degradation	0.69421883	0.18841941	0.00022921	0.00070344
PWY3DJ-35471: L-ascorbate biosynthesis IV (animals, D-glucuronate pathway)	0.67300283	0.19344038	0.00050306	0.00145601
PWY-7209: superpathway of pyrimidine ribonucleosides degradation	0.71580178	0.21074501	0.00068246	0.00194365
PWY-5464: superpathway of cytosolic glycolysis (plants), pyruvate dehydrogenase a	0.62722395	0.18829649	0.00086521	0.00236934
PWY-7328: superpathway of UDP-glucose-derived O-antigen building blocks biosyn	0.51659028	0.15792054	0.00107091	0.00286649
GLUCOSE1PMETAB-PWY: glucose and glucose-1-phosphate degradation	0.57974885	0.17750482	0.00109039	0.00288106
PWY-2723: trehalose degradation V	0.5835063	0.18419782	0.00153584	0.00393353
PWY-7389: superpathway of anaerobic energy metabolism (invertebrates)	0.65499976	0.21058479	0.00186844	0.00465149
P125-PWY: superpathway of (R,R)-butanediol biosynthesis	0.54361078	0.17775791	0.00222706	0.0055058
PWY-5845: superpathway of menaquinol-9 biosynthesis	0.62127357	0.20384522	0.00230545	0.00566029
PWY-5862: superpathway of demethylmenaquinol-9 biosynthesis	0.58004819	0.19439989	0.00284703	0.00684827
PWY-5265: peptidoglycan biosynthesis II (staphylococci)	0.70113094	0.23565387	0.00292743	0.00699439
GLYCOCAT-PWY: glycogen degradation I	0.56749634	0.19331406	0.00332883	0.00779648
PWY-6731: starch degradation III	0.52256477	0.179255	0.00355457	0.00821706
PWY0-166: superpathway of pyrimidine deoxyribonucleotides de novo biosynthesis	0.67449698	0.23321861	0.00382648	0.00878855
PWY-7198: pyrimidine deoxyribonucleotides de novo biosynthesis IV	0.11936442	0.04155895	0.00407662	0.00912752
PWY-7384: anaerobic energy metabolism (invertebrates, mitochondrial)	0.56842526	0.19783641	0.00406326	0.00912752
FOLSYN-PWY: superpathway of tetrahydrofolate biosynthesis and salvage	0.48300459	0.16917059	0.00430188	0.00951222
THREOCAT-PWY: superpathway of L-threonine metabolism	0.55821402	0.199099	0.00505198	0.01103376
PWY-6285: superpathway of fatty acids biosynthesis (E. coli)	0.71621048	0.25913569	0.00571249	0.01232514
PWY-6612: superpathway of tetrahydrofolate biosynthesis	0.44327813	0.16199791	0.00621306	0.0132446
PWY66-367: ketogenesis	0.44058203	0.16133359	0.00631662	0.01330602
P124-PWY: Bifidobacterium shunt	0.61452594	0.22492338	0.00629212	0.01330602
PWY-7196: superpathway of pyrimidine ribonucleosides salvage	0.58813482	0.21875443	0.00717605	0.0150275
PWY0-881: superpathway of fatty acid biosynthesis I (E. coli)	0.53316363	0.20040929	0.0078054	0.01624983
PWY-5838: superpathway of menaquinol-8 biosynthesis I	0.46893402	0.17894417	0.00877854	0.01806451
P161-PWY: acetylene degradation (anaerobic)	0.28007086	0.10733915	0.00907504	0.01856732
PWY-5656: mannosylglycerate biosynthesis I	0.49482015	0.18997666	0.00919718	0.01870968
PWY-5861: superpathway of demethylmenaquinol-8 biosynthesis I	0.44662823	0.17248517	0.00961512	0.01933889
KETOGLUCONMET-PWY: ketogluconate metabolism	0.54102003	0.21244486	0.01087664	0.02175327
PWY-821: superpathway of sulfur amino acid biosynthesis (Saccharomyces cerevisi	0.55301471	0.21887513	0.01151654	0.0229044
PWY4LZ-257: superpathway of fermentation (Chlamydomonas reinhardtii)	0.2576591	0.1036094	0.01288865	0.02549088
P23-PWY: reductive TCA cycle I	0.49987046	0.2031848	0.01388687	0.0273134
ORNDEG-PWY: superpathway of ornithine degradation	0.56209545	0.23678562	0.01760332	0.03405859
PWY-5897: superpathway of menaquinol-11 biosynthesis	0.41356388	0.17515666	0.0182204	0.03450246
PWY-5898: superpathway of menaquinol-12 biosynthesis	0.41356388	0.17515666	0.0182204	0.03450246
PWY-5899: superpathway of menaquinol-13 biosynthesis	0.41356388	0.17515666	0.0182204	0.03450246
REDCITCYC: TCA cycle VI (Helicobacter)	0.47211181	0.19971822	0.01808421	0.03450246
ARGDEG-PWY: superpathway of L-arginine, putrescine, and 4-aminobutanoate degr	0.50155812	0.21346173	0.0187916	0.03520952
ORNARGDEG-PWY: superpathway of L-arginine and L-ornithine degradation	0.50155812	0.21346173	0.0187916	0.03520952
P221-PWY: octane oxidation	0.39517703	0.16940993	0.01966561	0.03665422
PWY-6876: isopropanol biosynthesis (engineered)	0.37228057	0.16606913	0.02497944	0.04583856
PWY-5837: 2-carboxy-1,4-naphthoquinol biosynthesis	0.35062821	0.15655862	0.0251171	0.04585481
PWY-6595: superpathway of guanosine nucleotides degradation (plants)	0.27874976	0.12641552	0.02745213	0.04978792
P122-PWY: heterolactic fermentation	0.46835057	0.21253704	0.02755118	0.04978792
PWY-5392: reductive TCA cycle II	0.37374588	0.16986856	0.02779226	0.04996992

Supplementary Table 13: HMP2 IBD significantly enriched pathways from ANCOM-BC2

Differential abundances of MetaCyc pathways in IBD (N = 198) vs non-IBD (N=139) from the Human Microbiome Project 2 dataset (downloaded from https://www.ibdmdb.org/downloads/html/products_MGX_2017-08-12.html) were tested using ANCOM-BC2, while adjusting for covariates (age, diagnosis, and total sequence count per sample). Shown are the significantly enriched pathways that were detected by HUMAnN, however, only pathways that were present in at least 10% of samples were included in ANCOM-BC2 analysis. LFC: indicates the log fold change compared to healthy control or in the case of lfc_PDvsIBD it is the log fold change of PD compared to IBD. SE: standard error; Pval: the uncorrected, two-sided P-value reported by ANCOM-BC2; Adj. Pval aka q-value from the false discovery rate, i.e., multiple-testing corrected significance q-value, calculated using the Benjamini-Hochberg method, alpha =0.05.

pathway	lfc_IBD	se_IBD	pval_IBD	adj_pval_IBD
ANAEROFRUCAT-PWY: homolactic fermentation	0.23777702	0.10631361	0.02531496	0.04594197
AST-PWY: L-arginine degradation II (AST pathway)	0.8589247	0.179978	1.82E-06	1.57E-05
BIOTIN-BIOSYNTHESIS-PWY: biotin biosynthesis I	0.86357408	0.21775385	7.31E-05	0.00026269
CITRULBIO-PWY: L-citrulline biosynthesis	0.46716095	0.17601599	0.00795244	0.01694215
COLANSYN-PWY: colanic acid building blocks biosynthesis	0.49360487	0.15203522	0.00116773	0.00298532
ENTBACSYN-PWY: enterobactin biosynthesis	1.10087198	0.23777903	3.66E-06	2.76E-05
FASYN-ELONG-PWY: fatty acid elongation -- saturated	0.92630631	0.22018364	2.59E-05	0.00012897
FASYN-INITIAL-PWY: superpathway of fatty acid biosynthesis initiatio	0.88874457	0.21380201	3.23E-05	0.00014885
GALACTARDEG-PWY: D-galactarate degradation I	0.81436693	0.17711672	4.27E-06	3.06E-05
GLUCARDEG-PWY: D-glucarate degradation I	0.87592209	0.18657045	2.67E-06	2.18E-05
GLUCARGALACTSUPER-PWY: superpathway of D-glucarate and D-ga	0.81436693	0.17711672	4.27E-06	3.06E-05
GLUCOSE1PMETAB-PWY: glucose and glucose-1-phosphate degrada	0.8455685	0.23668216	0.00035347	0.00100892
GLYCOCAT-PWY: glycogen degradation I (bacterial)	0.83928462	0.22360708	0.00017446	0.0005399
GLYCOL-GLYOXDEG-PWY: superpathway of glycol metabolism and d	0.94669828	0.19703143	1.55E-06	1.47E-05
GLYCOLYSIS-TCA-GLYOX-BYPASS: superpathway of glycolysis, pyruv	0.8611239	0.24676783	0.00048372	0.00134165
GLYCOLYSIS: glycolysis I (from glucose 6-phosphate)	0.45549926	0.12264712	0.00020409	0.00060608
GLYOXYLATE-BYPASS: glyoxylate cycle	0.75745325	0.23134605	0.00105994	0.00278234
HEME-BIOSYNTHESIS-II: heme biosynthesis I (aerobic)	0.55011459	0.20955959	0.00866239	0.01819102
KDO-NAGLIPASYN-PWY: superpathway of (Kdo)2-lipid A biosynthesis	0.90744764	0.18726942	1.26E-06	1.26E-05
KETOGLUCONMET-PWY: ketogluconate metabolism	0.76862589	0.1928849	6.75E-05	0.00025446
METHGLYUT-PWY: superpathway of methylglyoxal degradation	0.47856957	0.19589413	0.0145657	0.02854876
NAD-BIOSYNTHESIS-II: NAD salvage pathway II	0.87700108	0.25076555	0.00047	0.00131601
P105-PWY: TCA cycle IV (2-oxoglutarate decarboxylase)	0.89698974	0.2386644	0.00017102	0.0005349
PHOSLIPSYN-PWY: superpathway of phospholipid biosynthesis I (bac	0.42807525	0.17944191	0.01705164	0.03276589
POLYISOPRENSYN-PWY: polyisoprenoid biosynthesis (E. coli)	0.89869445	0.20456167	1.12E-05	6.84E-05
PWY-2723: trehalose degradation V	0.82401089	0.19123675	1.64E-05	9.10E-05
PWY-4041: γ-glutamyl cycle	0.96535918	0.23175559	3.11E-05	0.00014737
PWY-4984: urea cycle	0.52548695	0.17476696	0.00264025	0.00631084
PWY-5083: NAD/NADH phosphorylation and dephosphorylation	0.67756937	0.23482469	0.00390884	0.00919359
PWY-5173: superpathway of acetyl-CoA biosynthesis	0.63033258	0.17575127	0.00033514	0.000966
PWY-5189: tetrapyrrole biosynthesis II (from glycine)	1.00563326	0.2348497	1.85E-05	0.00010058
PWY-5484: glycolysis II (from fructose 6-phosphate)	0.48365008	0.12086146	6.29E-05	0.00024329
PWY-5505: L-glutamate and L-glutamine biosynthesis	0.71382089	0.20719074	0.00057056	0.00154015
PWY-561: superpathway of glyoxylate cycle and fatty acid degradatio	0.55905846	0.18522689	0.00254251	0.00612703
PWY-5705: allantoin degradation to glyoxylate III	0.75586355	0.17419929	1.43E-05	8.41E-05
PWY-5723: Rubisco shunt	1.00937202	0.23675124	2.01E-05	0.00010569
PWY-5838: superpathway of menaquinol-8 biosynthesis I	0.94766035	0.24590323	0.0001163	0.00038419
PWY-5845: superpathway of menaquinol-9 biosynthesis	0.94152312	0.23743326	7.33E-05	0.00026269
PWY-5850: superpathway of menaquinol-6 biosynthesis I	0.94152312	0.23743326	7.33E-05	0.00026269
PWY-5855: ubiquinol-7 biosynthesis (prokaryotic)	0.82259193	0.20808258	7.71E-05	0.00026362
PWY-5856: ubiquinol-9 biosynthesis (prokaryotic)	0.82259193	0.20808258	7.71E-05	0.00026362
PWY-5857: ubiquinol-10 biosynthesis (prokaryotic)	0.82259193	0.20808258	7.71E-05	0.00026362
PWY-5860: superpathway of demethylmenaquinol-6 biosynthesis I	0.91784469	0.22541354	4.66E-05	0.00019047
PWY-5861: superpathway of demethylmenaquinol-8 biosynthesis	0.92057634	0.23315713	7.87E-05	0.00026597
PWY-5862: superpathway of demethylmenaquinol-9 biosynthesis	0.91784469	0.22541354	4.66E-05	0.00019047
PWY-5896: superpathway of menaquinol-10 biosynthesis	0.94152312	0.23743326	7.33E-05	0.00026269
PWY-5913: TCA cycle VI (obligate autotrophs)	1.00148294	0.24097895	3.24E-05	0.00014885

pathway	lfc_IBD	se_IBD	pval_IBD	adj_pval_IBD
PWY-5918: superpathay of heme biosynthesis from glutamate	0.6498427	0.21502499	0.00250967	0.00609788
PWY-6147: 6-hydroxymethyl-dihydropterin diphosphate biosynthesis	0.59193842	0.22096213	0.00738615	0.01608539
PWY-6282: palmitoleate biosynthesis I (from (5Z)-dodec-5-enoate)	0.82735505	0.21485547	0.00011776	0.00038467
PWY-6519: 8-amino-7-oxononanoate biosynthesis I	0.87389907	0.21488787	4.77E-05	0.00019059
PWY-6628: superpathway of L-phenylalanine biosynthesis	0.98786529	0.26690813	0.00021463	0.00063101
PWY-6629: superpathway of L-tryptophan biosynthesis	0.95990679	0.23556533	4.60E-05	0.00019047
PWY-6630: superpathway of L-tyrosine biosynthesis	0.90226697	0.26312555	0.00060573	0.00160436
PWY-6708: ubiquinol-8 biosynthesis (prokaryotic)	0.82259193	0.20808258	7.71E-05	0.00026362
PWY-6803: phosphatidylcholine acyl editing	0.90668972	0.23765433	0.00013609	0.00043491
PWY-6859: all-trans-farnesol biosynthesis	0.81399107	0.19892251	4.28E-05	0.00018782
PWY-6891: thiazole biosynthesis II (Bacillus)	0.81716907	0.21507868	0.00014505	0.00045854
PWY-6892: thiazole biosynthesis I (E. coli)	0.55873752	0.24533487	0.02275946	0.04205629
PWY-6895: superpathway of thiamin diphosphate biosynthesis II	0.88351223	0.27067973	0.00109832	0.0028325
PWY-7198: pyrimidine deoxyribonucleotides de novo biosynthesis IV	0.61012987	0.24891244	0.01423877	0.02828512
PWY-7204: pyridoxal 5'-phosphate salvage II (plants)	0.93395089	0.22859492	4.40E-05	0.00019006
PWY-7210: pyrimidine deoxyribonucleotides biosynthesis from CTP	0.59727177	0.25581059	0.01955281	0.03684952
PWY-7220: adenosine deoxyribonucleotides de novo biosynthesis II	0.31600772	0.12366475	0.01060786	0.02150835
PWY-7222: guanosine deoxyribonucleotides de novo biosynthesis II	0.31600772	0.12366475	0.01060786	0.02150835
PWY-7254: TCA cycle VII (acetate-producers)	0.87350431	0.21889966	6.60E-05	0.00025182
PWY-7269: NAD/NADP-NADH/NADPH mitochondrial interconversion	1.00605521	0.2172849	3.65E-06	2.76E-05
PWY-7315: dTDP-N-acetylthomosamine biosynthesis	1.14905876	0.22335961	2.68E-07	3.29E-06
PWY-7323: superpathway of GDP-mannose-derived O-antigen buildir	0.98753529	0.16866085	4.77E-09	1.27E-07
PWY-7388: octanoyl-[acyl-carrier protein] biosynthesis (mitochondria	0.91156639	0.21076453	1.52E-05	8.79E-05
PWY-7409: phospholipid remodeling (phosphatidylethanolamine, yea	0.86683026	0.20624415	2.63E-05	0.0001291
PWY-7539: 6-hydroxymethyl-dihydropterin diphosphate biosynthesis	0.56200401	0.21581118	0.00921033	0.01920452
PWY-7664: oleate biosynthesis IV (anaerobic)	0.89892861	0.2184921	3.88E-05	0.0001757
PWY0-1241: ADP-L-glycero-β-D-manno-heptose biosynthesis	0.62760387	0.21776616	0.00395149	0.00922015
PWY0-1297: superpathway of purine deoxyribonucleosides degradati	0.51750149	0.13900323	0.00019691	0.00059074
PWY0-1338: polymyxin resistance	0.89901823	0.18359575	9.74E-07	1.04E-05
PWY0-1415: superpathway of heme biosynthesis from uroporphyrino	0.85137548	0.17989842	2.22E-06	1.86E-05
PWY0-1533: methylphosphonate degradation I	0.9346665	0.19479463	1.60E-06	1.47E-05
PWY0-862: (5Z)-dodec-5-enoate biosynthesis	0.88039239	0.21610537	4.62E-05	0.00019047
PWY4FS-7: phosphatidylglycerol biosynthesis I (plastidic)	0.44258618	0.13745614	0.00128264	0.00322305
PWY4FS-8: phosphatidylglycerol biosynthesis II (non-plastidic)	0.44258727	0.13745611	0.0012826	0.00322305
PWY66-400: glycolysis VI (metazoan)	0.46562216	0.17671357	0.00841627	0.01780132
PWY66-409: superpathway of purine nucleotide salvage	0.84675943	0.24088894	0.00043951	0.00124247
PWYG-321: mycolate biosynthesis	0.57111836	0.22002174	0.00943885	0.01954241
PYRIDNUCSAL-PWY: NAD salvage pathway I	0.68922645	0.24956627	0.00575018	0.01300425
REDCITCYC: TCA cycle VIII (helicobacter)	0.51500141	0.20116699	0.01046513	0.02150835
RHAMCAT-PWY: L-rhamnose degradation I	0.35029287	0.12759863	0.00604609	0.01348101
TCA-GLYOX-BYPASS: superpathway of glyoxylate bypass and TCA	0.89512562	0.23453497	0.0001353	0.00043491
UBISYN-PWY: superpathway of ubiquinol-8 biosynthesis (prokaryotic	0.81490439	0.19915503	4.28E-05	0.00018782

Supplementary Table 14: Analysis of confounders on significant taxa in Wallen PD.

N= 490 PD, N= 234 control samples were examined. Eleven variables, including Diagnosis and total sequence count per sample along with nine potential confounders, were included in a single model and tested simultaneously for association with bias corected relative abundances extracted from ANCOM-BC2 analysis of the 11 PD associated species as identified from ANCOM-BC2 analysis. Multivariate linear regression was computed in R using the lm() funciton with BH multiple comparisons p-value adjustment. The variables included were sex (M vs F), Age (continuous variable, standardized using scale function in R), Diagnosis (PD or Control), total sequences aka total read count per sample (continuous variable, standardized using scale function in R), Laxatives (Yes or No), Pain meds (Yes or No), Depression_anxiety__mood_meds (Yes or No), and Birth control_or_estrogen (Yes or No), Antihistamines (Yes or No), Probiotic (Yes or No), and Sleep aids (Yes or No). Regression was performed with and without the influence of confounding variables, and the coefficients associated with the outcome variable, in this case association with PD, were extracted. The ratio of coefficients with and without confounding variables were used to determine if confounders appeared to influence the findings (ratio < 1 or sign reversal (-)).

Taxa	Coefficient_Crude	Coefficient_Adjusted	Difference	Percent_Change	Ratio	Adjustment moves coefficient towards null?
Actinomyces_oris	2.063411202	2.091064729	-0.027653526	-1.32246152	**1.013402**	**FALSE**
Bifidobacterium_dentium	2.111989116	2.005224598	0.106764518	5.324317191	**0.949448**	**TRUE**
Streptococcus_mutans	1.885920798	1.65592117	0.229999628	13.88952761	**0.878044**	**TRUE**
Anaerostipes_hadrus	-1.514958085	-1.665926327	0.150968242	-9.062119954	**1.099652**	**FALSE**
Blautia_wexlerae	-1.44586286	-1.323543299	-0.122319561	9.241825428	**0.9154**	**TRUE**
Eisenbergiella_tayi	1.431777097	1.382986451	0.048790646	3.52791931	**0.965923**	**TRUE**
Roseburia_intestinalis	-1.902184459	-1.723662726	-0.178521733	10.35711513	**0.906149**	**TRUE**
Clostridium_leptum	1.618515651	1.762449098	-0.143933447	-8.166672562	**1.088929**	**FALSE**
Ruminococcaceae_bacte	1.564841925	1.781916305	-0.21707438	-12.18207498	**1.13872**	**FALSE**
Ruminococcus_lactaris	-1.646043158	-1.388598373	-0.257444784	18.53990246	**0.843598**	**TRUE**
Escherichia_coli	1.609898208	0.844779862	0.765118346	90.570145	**0.524741**	**TRUE**

Supplementary Table 15: Analysis of confounders on significant taxa in HMP2 IBD.

N= 198 IBD, 138 non-IBD samples were examined. Eight variables, including Diagnosis and total read count per sample along with six potential confounders, were included in a single model and tested simultaneously for association with bias corected relative abundances extracted from ANCOM-BC2 analysis of the HMP2 IBD associated species as identified from ANCOM-BC2 analysis. Multivariate linear regression was computed in R using the lm() funciton with BH multiple comparisons p-value adjustment. The variables included were sex (M vs F), Age (continuous variable, standardized using scale function in R), Diagnosis (diagnosis2) (IBD or non-IBD), Reads aka total read count per sample (continuous variable, standardized using scale function in R), Site (MGH or Cedar-Sinai), Probiotic (Yes or No), Immunosuppressants (Yes or No), and Antibiotics (Yes or No). Regression was performed with and without the influence of confounding variables, and the coefficients associated with the outcome variable, in this case association with IBD, were extracted. The ratio of coefficients with and without confounding variables were used to determine if confounders appeared to influence the findings (ratio < 1 or sign reversal (-)).

Taxa	Coefficient_Without_Confoundin	Coefficient_With_Confoundin	Difference	Percent_Change	Ratio	Adjustment moves coefficient towards null?
Bifidobacterium_adolesc	-3.235483295	-2.697783842	-0.5377	16.61882953	**0.833812**	**TRUE**
Bacteroides_eggerthii	-3.105027743	0.469151621	-3.57418	115.109418	**-0.15109**	**TRUE**
Bacteroides_fragilis	-2.569776069	-4.970038042	2.400262	-93.40354599	**1.934035**	**FALSE**
Bacteroides_massiliensis	-3.003415897	-1.243718026	-1.7597	58.58988335	**0.414101**	**TRUE**
Bacteroides_nordii	-1.634051586	-2.133395663	0.499344	-30.55864824	**1.305586**	**FALSE**
Bacteroides_stercoris	2.140412236	2.91122438	-0.77081	-36.01232188	**1.360123**	**FALSE**
Bacteroides_thetaiotaom	-2.159402446	-2.611628256	0.452226	-20.94217366	**1.209422**	**FALSE**
Bacteroidales_bacterium	-3.760089465	-0.124157932	-3.63593	96.69800592	**0.03302**	**TRUE**
Barnesiella_intestinihomi	-4.549457355	-0.006904162	-4.54255	99.84824207	**0.001518**	**TRUE**
Coprobacter_fastidiosus	-1.676722754	-3.031307704	1.354585	-80.78765236	**1.807877**	**FALSE**
Odoribacter_laneus	-2.589105737	-2.145629742	-0.44348	17.12853934	**0.828715**	**TRUE**
Odoribacter_unclassified	-1.83104596	-3.076118174	1.245072	-67.99786792	**1.679979**	**FALSE**
Parabacteroides_distaso	1.868250576	1.075420681	0.79283	42.43702132	**0.57563**	**TRUE**
Parabacteroides_goldstei	-2.744941719	-1.346914113	-1.39803	50.93104879	**0.49069**	**TRUE**
Parabacteroides_unclass	1.974673452	0.809435007	1.165238	59.00917157	**0.409908**	**TRUE**
Prevotella_copri	-2.87878212	0.116160213	-2.99494	104.0350471	**-0.04035**	**TRUE**
Alistipes_indistinctus	-2.514432246	-2.457615665	-0.05682	2.259618702	**0.977404**	**TRUE**
Alistipes_onderdonkii	-3.571957625	-3.940378804	0.368421	-10.3142651	**1.103143**	**FALSE**
Alistipes_putredinis	-6.512435454	-6.800452025	0.288017	-4.422563159	**1.044226**	**FALSE**
Alistipes_senegalensis	-1.708834688	-0.744913058	-0.96392	56.40812633	**0.435919**	**TRUE**
Alistipes_shahii	-5.115230943	-1.717042982	-3.39819	66.43273782	**0.335673**	**TRUE**
Clostridium_asparagiform	1.628962679	-1.027595034	2.656558	163.0827856	**-0.63083**	**TRUE**
Clostridium_bolteae	4.062987081	0.52260131	3.540386	87.13750992	**0.128625**	**TRUE**
Clostridium_clostridiofor	3.321887283	-0.372179562	3.694067	111.2038588	**-0.11204**	**TRUE**
Clostridium_hathewayi	2.453470565	2.713964008	-0.26049	-10.61734536	**1.106173**	**FALSE**
Clostridium_symbiosum	1.629487279	0.53976959	1.089718	66.87488166	**0.331251**	**TRUE**
Clostridiales_bacterium_	1.389028017	-0.09617688	1.485205	106.9240418	**-0.06924**	**TRUE**
Flavonifractor_plautii	2.472134077	-0.299479943	2.771614	112.1142274	**-0.12114**	**TRUE**
Eubacterium_eligens	-2.715153892	-2.395744706	-0.31941	11.76394411	**0.882361**	**TRUE**
Eubacterium_hallii	-2.73365794	-1.444125533	-1.28953	47.17241277	**0.528276**	**TRUE**
Eubacterium_ramulus	-3.644731739	-3.844521341	0.19979	-5.481599639	**1.054816**	**FALSE**
Eubacterium_rectale	-3.684156668	-2.072450619	-1.61171	43.74694655	**0.562531**	**TRUE**
Eubacterium_ventriosum	-3.127448949	0.597599234	-3.72505	119.1082011	**-0.19108**	**TRUE**
Anaerostipes_hadrus	-1.69728954	-2.362495227	0.665206	-39.19223396	**1.391922**	**FALSE**
Ruminococcus_gnavus	2.951462941	-0.288099082	3.239562	109.7612299	**-0.09761**	**TRUE**
Ruminococcus_obeum	-1.389333652	-0.990614395	-0.39872	28.69859635	**0.713014**	**TRUE**
Butyrivibrio_crossotus	-2.315150035	0.706740751	-3.02189	130.5267797	**-0.30527**	**TRUE**
Coprococcus_catus	-2.147450092	0.38668845	-2.53414	118.0068655	**-0.18007**	**TRUE**
Coprococcus_comes	-2.585180266	-0.327263002	-2.25792	87.34080535	**0.126592**	**TRUE**

Taxa	Coefficient_Without_Confoundin	Coefficient_With_Confounding	Difference	Percent_Change	Ratio	Adjustment moves coefficient towards null?
Coprococcus_sp_ART55_	-2.672500239	0.244024913	-2.91653	109.1309594	**-0.09131**	**TRUE**
Dorea_formicigenerans	-3.483214275	-1.175629253	-2.30759	66.2487243	**0.337513**	**TRUE**
Dorea_unclassified	-1.848933803	-3.269762939	1.420829	-76.84586295	**1.768459**	**FALSE**
Lachnospiraceae_bacteri	-1.822841252	-3.786255399	1.963414	-107.7117464	**2.077117**	**FALSE**
Lachnospiraceae_bacteri	-3.222074681	-3.558849906	0.336775	-10.45212352	**1.104521**	**FALSE**
Roseburia_hominis	-4.710984338	-4.321085356	-0.3899	8.276380348	**0.917236**	**TRUE**
Roseburia_intestinalis	-3.629692582	-3.998875081	0.369182	-10.17117816	**1.101712**	**FALSE**
Roseburia_inulinivorans	-4.682340311	-4.226690072	-0.45565	9.731249935	**0.902688**	**TRUE**
Peptostreptococcaceae_	-1.519425341	-1.561654857	0.04223	-2.779308406	**1.027793**	**FALSE**
Faecalibacterium_prausn	-3.959259231	-2.230798167	-1.72846	43.65617312	**0.563438**	**TRUE**
Ruminococcus_bromii	-2.576639598	0.148844138	-2.72548	105.7766766	**-0.05777**	**TRUE**
Ruminococcus_callidus	-1.659803554	-1.989793606	0.32999	-19.88127158	**1.198813**	**FALSE**
Subdoligranulum_unclass	-2.354512408	-2.513655683	0.159143	-6.759075649	**1.067591**	**FALSE**
Coprobacillus_unclassifi	1.31929129	0.648514029	0.670777	50.84375723	**0.491562**	**TRUE**
Eubacterium_biforme	-2.660222844	-2.312127574	-0.3481	13.08519214	**0.869148**	**TRUE**
Veillonella_atypica	-1.297215208	-1.623688458	0.326473	-25.16723889	**1.251672**	**FALSE**
Veillonella_dispar	-1.487179254	-1.620617056	0.133438	-8.972543304	**1.089725**	**FALSE**
Veillonella_parvula	-2.271395117	-2.576422623	0.305028	-13.4290817	**1.134291**	**FALSE**
Veillonella_unclassified	-2.529646258	-2.382963771	-0.14668	5.798537511	**0.942015**	**TRUE**
Oxalobacter_formigenes	-1.645262984	-0.493818389	-1.15144	69.98544344	**0.300146**	**TRUE**
Sutterella_wadsworthens	-2.301744879	-1.006476617	-1.29527	56.27332005	**0.437267**	**TRUE**
Bilophila_unclassified	-2.872218124	-1.330005467	-1.54221	53.69413429	**0.463059**	**TRUE**
Escherichia_coli	1.857759916	4.559255339	-2.7015	-145.4168217	**2.454168**	**FALSE**
Haemophilus_parainfluer	-1.915340614	-2.397967553	0.482627	-25.19796923	**1.25198**	**FALSE**
Akkermansia_muciniphila	-2.281341941	-0.682440075	-1.5989	70.08602425	**0.29914**	**TRUE**

Supplementary Table 16: Analysis of confounders on beta diversity in UFPF.

To assess the influence of demographic and clinical covariates on the variation in microbial beta-diversity, we performed a Permutational Multivariate Analysis of Variance (PERMANOVA) using the adonis2 function from the vegan R package. The covariates included were Sex (M vs F), Age (continuous variable, Diagnosis (PD, IBD, or control), standardized using scale function in R), Reads aka total read count per sample (continuous variable, standardized using scale function in R), Indigestion_meds (Yes or No), Anti_TNF meds (Yes or No), Anti_inflammatories (Yes or No), Depression_anxiety_meds (Yes or No), Iron_specific_supplement (Yes or No). A PERMANOVA was conducted on the Euclidean distance matrix of the centered log-ratio (CLR) transformed microbial abundances to evaluate the extent to which these covariates explained the variation in microbial communities across samples. Statistical significance was assessed using 999 permutations. Covariates tested in the model showed significant associations with beta-diversity when Pr(>F) < 0.05, indicating that differences in microbial composition were significantly influenced by the combined effects of demographic factors, clinical factors, and/or technical factors.

Covariate	Df	SumOfSqs	R2	F	Pr(>F)	
Sex	1	79442.43	0.013464	1.343958	0.044	*
Age	1	105501.3	0.017881	1.784806	0.002	*
Diagnosis	2	280909.2	0.04761	2.376125	0.001	*
Reads	1	169154.9	0.02867	2.861658	0.001	*
Indigestion_meds	1	57399.23	0.009728	0.971045	0.489	
Anti_TNF	1	41255.35	0.006992	0.697933	0.977	
Anti_inflammatories	1	47409.79	0.008035	0.80205	0.866	
Depression_anxiety_meds	1	54070.36	0.009164	0.914729	0.675	
Iron_specific_supplement	1	40594.44	0.00688	0.686752	0.978	
Residual	85	5024418	0.851574			
Total	95	5900155	1			

Supplementary Table 17: Analysis of confounders on beta diversity in Wallen PD.

To assess the influence of demographic and clinical covariates on the variation in microbial beta-diversity, we performed a Permutational Multivariate Analysis of Variance (PERMANOVA) using the adonis2 function from the vegan R package. The covariates included were diagnosis (PD or control), sex (M vs F), Age (continuous variable, standardized using scale function in R), total_sequences aka total read count per sample (continuous variable, standardized using scale function in R), Laxatives (Yes or No), Pain_med (Yes or No), Depression_anxiety_mood_med (Yes or No), Birth_control_or_estrogen (Yes or No), Antihistamines (Yes or No), Probiotic (Yes or No), Sleep_aid (Yes or No). A PERMANOVA was conducted on the Euclidean distance matrix of the centered log-ratio (CLR) transformed microbial abundances to evaluate the extent to which these covariates explained the variation in microbial communities across samples. Statistical significance was assessed using 999 permutations. Covariates tested in the model showed significant associations with beta-diversity when Pr(>F) < 0.05, indicating that differences in microbial composition were significantly influenced by the combined effects of demographic factors, clinical factors, and/or technical factors.

Covariate	Df	SumOfSqs	R2	F	Pr(>F)	
Diagnosis	1	171028.6	0.005718	3.808252	0.001	*
Sex	1	265385.2	0.008872	5.909268	0.001	*
Age	1	133678.9	0.004469	2.976596	0.001	*
total_sequences	1	171429.7	0.005731	3.817185	0.001	*
Laxatives	1	104264.3	0.003486	2.321629	0.001	*
Pain_med	1	81381.57	0.002721	1.812104	0.002	
Depression_anxiety_mood_med	1	105001.3	0.00351	2.338039	0.001	*
Birth_control_or_estrogen	1	49478.66	0.001654	1.10173	0.204	
Antihistamines	1	36714.98	0.001227	0.817524	0.904	
Probiotic	1	55489.28	0.001855	1.235567	0.081	
Sleep_aid	1	39942.63	0.001335	0.889393	0.765	
Residual	639	28697482	0.95942			
Total	650	29911277	1			

Supplementary Table 18: Analysis of confounders on beta diversity in HMP2 IBD.

To assess the influence of demographic and clinical covariates on the variation in microbial beta-diversity, we performed a Permutational Multivariate Analysis of Variance (PERMANOVA) using the adonis2 function from the vegan R package. The covariates included were Sex (M vs F), Age (continuous variable, Diagnosis (IBD or non-IBD), standardized using scale function in R), Reads aka total read count per sample (continuous variable, standardized using scale function in R), Site (MGH or Cedar-Sinai), Probiotic (Yes or No), Immunosuppressants (Yes or No), and Antibiotics (Yes or No). A PERMANOVA was conducted on the Euclidean distance matrix of the centered log-ratio (CLR) transformed microbial abundances to evaluate the extent to which these covariates explained the variation in microbial communities across samples. Statistical significance was assessed using 999 permutations. Covariates tested in the model showed significant associations with beta-diversity when Pr(>F) < 0.05, indicating that differences in microbial composition were significantly influenced by the combined effects of demographic factors, clinical factors, and/or technical factors.

Covariate	Df	SumOfSqs	R2	F	Pr(>F)	
Sex	1	2204.067	0.029814	11.13019	0.001	*
Age	1	1137.702	0.015389	5.745214	0.001	*
Diagnosis	1	1936.184	0.02619	9.777423	0.001	*
Reads	1	555.7443	0.007517	2.806421	0.001	*
Site	1	1426.906	0.019301	7.205647	0.001	*
Probiotic	1	1056.216	0.014287	5.333725	0.001	*
Immunosuppressants	1	968.5323	0.013101	4.890935	0.001	*
Antibiotics	1	481.8284	0.006518	2.433157	0.001	*
Residual	324	64160.43	0.867882			
Total	332	73927.61	1			

WGCNA Module List - UFPF Dataset (Species)

Below is the module list extracted from our WGCNA analysis (of the UFPF dataset) displaying specific microbial species and their module assignments- ns (not-significant), down, or up.

Species	Module
Corynebacterium_amycolatum	ns
GGB2945_SGB3917	ns
Fenollaria_timonensis	ns
Ezakiella_SGB6726	ns
Varibaculum_cambriense	ns
Porphyromonas_SGB1983	ns
Clostridiales_bacterium_SIT11	ns
Prevotella_buccalis	ns
Collinsella_aerofaciens	ns
Peptoniphilus_grossensis	ns
Corynebacterium_aurimucosum	ns
Mobiluncus_SGB15488	ns
GGB9715_SGB15265	ns
Porphyromonas_SGB1977	ns
Alterileibacterium_massiliense	ns
Peptococcus_niger	ns
Gemmiger_formicilis	ns
Peptoniphilus_sp_Marseille_P3761	ns
GGB1456_SGB2019	ns
Oscillibacter_sp_ER4	ns
Peptoniphilus_pacaensis	ns
Alistipes_putredinis	ns
Ruminococcus_bicirculans	ns
Evtepia_gabavorous	ns
Blautia_obeum	ns
Faecalibacterium_prausnitzii	ns
Phascolarctobacterium_faecium	ns
GGB9512_SGB14909	ns
Porphyromonas_bennonis	ns
Bacteroides_uniformis	ns
Bifidobacterium_adolescentis	ns
Wujia_chipingensis	ns
Dorea_longicatena	ns
Eubacterium_hallii	ns
Lagierella_massiliensis	ns
Clostridiaceae_unclassified_SGB150[illegible]	ns
Acidaminococcus_intestini	ns
Barnesiella_intestinihominis	ns
Adlercreutzia_equolifaciens	ns
Mediterraneibacter_faecis	ns

Species	Module
Blautia_faecis	ns
Candidatus_Cibionibacter_quicibialis	ns
Eubacterium_rectale	ns
Faecalicatena_fissicatena	ns
Fusicatenibacter_saccharivorans	ns
Ruminococcus_bromii	ns
Agathobaculum_butyriciproducens	ns
Phocaeicola_vulgatus	ns
Blautia_sp_MCC283	ns
Brotolimicola_acetigignens	ns
Eubacteriales_Family_XIII_Incertae_Se	ns
GGB9715_SGB15260	ns
Olegusella_massiliensis	ns
GGB4277_SGB5832	ns
Phocaeicola_dorei	ns
Ruminococcus_lactaris	down
Alistipes_indistinctus	ns
Dorea_formicigenerans	ns
Oscillibacter_valericigenes	ns
Oscillospiraceae_bacterium_CLA_AA_	ns
Oliverpabstia_intestinalis	ns
Blautia_wexlerae	down
Alistipes_communis	ns
Alistipes_shahii	ns
Blautia_massiliensis	ns
Coprococcus_comes	ns
Parabacteroides_merdae	ns
Lawsonella_SGB3665	ns
Bacteroides_massiliensis	ns
Ruminococcus_torques	ns
Alistipes_onderdonkii	ns
Negativibacillus_massiliensis	ns
GGB3433_SGB4573	ns
Bacteroides_intestinalis	ns
Escherichia_coli	up
Dysosmobacter_welbionis	ns
Bacteroides_stercoris	ns
Parabacteroides_distasonis	ns
Paraprevotella_clara	ns
GGB3167_SGB4181	ns
Bacteroides_faecis	ns
Monoglobus_pectinilyticus	ns
Streptococcus_parasanguinis	ns
Bacteroides_finegoldii	ns
GGB51647_SGB4348	ns

Species	Module
Clostridiales_bacterium	ns
Eisenbergiella_tayi	up
Corynebacterium_simulans	ns
Blautia_caecimuris	ns
Lawsonibacter_hominis	ns
Bacteroides_ovatus	ns
Anaerostipes_hadrus	down
Bacteroides_caccae	ns
Faecalibacterium_sp_CLA_AA_H233	ns
Ruthenibacterium_lactatiformans	ns
Eggerthella_lenta	ns
Bilophila_wadsworthia	ns
Clostridium_sp_AF36_4	ns
Clostridium_clostridioforme	ns
Clostridium_scindens	ns
Roseburia_inulinivorans	ns
Parasutterella_excrementihominis	ns
Anaerotruncus_massiliensis	ns
Akkermansia_muciniphila	ns
Blautia_hansenii	ns
Clostridium_symbiosum	ns
Enterocloster_hominis	ns
Blautia_hydrogenotrophica	ns
Eubacterium_ramulus	ns
Blautia_producta	ns
Intestinimonas_massiliensis	ns
Ruminococcus_gnavus	ns
Clostridium_bolteae	ns
Eubacterium_eligens	ns
Neglectibacter_timonensis	ns
Roseburia_hominis	ns
Corynebacterium_tuberculostearicum	ns
Klebsiella_pneumoniae	ns
Porphyromonas_uenonis	ns
Lawsonibacter_asaccharolyticus	ns
Blautia_glucerasea	ns
Roseburia_intestinalis	down
Roseburia_faecis	ns
Faecalimonas_umbilicata	ns
Flavonifractor_plautii	ns
Dialister_invisus	ns
Lachnospira_pectinoschiza	ns
Bifidobacterium_longum	ns
Eisenbergiella_massiliensis	ns
Finegoldia_magna	ns

Species	Module
Streptococcus_salivarius	ns
Corynebacterium_pseudogenitalium	ns
Enterocloster_aldenensis	ns
Anaerococcus_prevotii	ns
Clostridium_leptum	up
Faecalibacterium_SGB15346	ns
GGB9342_SGB14306	ns
GGB9708_SGB15234	ns
GGB3653_SGB4964	ns
GGB36331_SGB15121	ns
Coprococcus_catus	ns
GGB9365_SGB14341	ns
Blautia_luti	ns
Clostridiaceae_bacterium	ns
GGB9730_SGB15291	ns
Faecalibacterium_sp_HTFF	ns
Clostridium_fessum	ns
Sutterella_wadsworthensis	ns
Lachnospiraceae_bacterium_CLA_AA	ns
GGB32900_SGB53446	ns
GGB9635_SGB15106	ns
Lentihominibacter_faecis	ns
Odoribacter_splanchnicus	ns
Methanobrevibacter_smithii	ns
Lachnospiraceae_bacterium	ns
GGB9760_SGB15373	ns
Desulfovibrio_fairfieldensis	ns
GGB9699_SGB15216	ns
Blautia_SGB4815	ns
Clostridiaceae_bacterium_AF18_31LB	ns
Clostridium_sp_AM22_11AC	ns
Lacrimispora_amygdalina	ns
GGB2980_SGB3962	ns
Dorea_sp_AF36_15AT	ns
GGB1215_SGB1581	ns
Levyella_massiliensis	ns
Porphyromonas_sp_HMSC065F10	ns
Prevotella_bivia	ns
Parabacteroides_goldsteinii	ns
Peptoniphilus_urinimassiliensis	ns
Clostridiales_bacterium_S5_A14a	ns
GGB3109_SGB4121	ns
Bacteroides_thetaiotaomicron	ns
GGB9480_SGB14874	ns
Anaerococcus_sp_Marseille_P3625	ns

Species	Module
Clostridium_innocuum	ns
Prevotella_corporis	ns
Anaerococcus_obesiensis	ns
Clostridium_hathewayi	ns
Streptococcus_thermophilus	ns
Bifidobacterium_animalis	ns
Bacteroides_eggerthii	ns
Anaerobutyricum_soehngenii	ns
Bacteroides_xylanisolvens	ns
GGB9345_SGB14311	ns
Oscillibacter_sp_MSJ_31	ns
Alistipes_finegoldii	ns
Campylobacter_hominis	ns
Vescimonas_coprocola	ns
GGB9453_SGB14844	ns
Oscillospiraceae_bacterium	ns
Clostridiales_bacterium_KLE1615	ns
Anaerotignum_faecicola	ns
GGB9770_SGB15390	ns
Bacteroides_cellulosilyticus	ns
Eubacterium_siraeum	ns
Bifidobacterium_bifidum	ns
Bifidobacterium_pseudocatenulatum	ns
Bacteroides_nordii	ns
GGB3433_SGB4574	ns
GGB38744_SGB14842	ns
Blautia_stercoris	ns
Porphyromonas_somerae	ns
Porphyromonas_asaccharolytica	ns
Porphyromonas_SGB1980	ns
Peptoniphilus_lacrimalis	ns
Lawsonella_clevelandensis	ns
Schaalia_turicensis	ns
Prevotella_timonensis	ns
Peptoniphilus_harei	ns
Prevotella_bergensis	ns
GGB9524_SGB14924	ns
Arcanobacterium_urinimassiliense	ns
Urinicoccus_timonensis	ns
Bacteroides_fragilis	ns
Anaerococcus_SGB6678	ns
Bacteroides_salyersiae	ns
Sellimonas_intestinalis	ns
Ezakiella_coagulans	ns
Fenollaria_massiliensis	ns

Species	Module
Mediterraneibacter_glycyrrhizinilyticu	ns
Clostridium_SGB4750	ns
GGB1455_SGB2018	ns
Campylobacter_ureolyticus	ns
Peptoniphilus_gorbachii	ns
Anaerococcus_mediterraneensis	ns
Butyricimonas_virosa	ns
Frisingicoccus_SGB4674	ns
Anaerococcus_murdochii	ns
GGB10524_SGB17049	ns
Streptococcus_anginosus	ns
Erysipelatoclostridium_ramosum	ns
Clostridium_sp_AT4	ns

WGCNA Module Scores - UFPF Dataset (Species)

Module scores refer to the average abundance of species in each module. Module scores are represented by the module eigengene, the first principal component of the species in each module and represents the overall abundance or expression trend of that module across samples.

Sample	down	ns	up
UF.PF.2020.001.1N	-0.044833452	0.038913183	-0.761331197
UF.PF.2020.001.2G	0.012033602	-0.053618122	0.459891606
UF.PF.2020.002.2G	1.377541284	0.028560369	-0.006255159
UF.PF.2020.002.3N	0.589793957	0.053483845	-0.490773912
UF.PF.2020.003.1N	-0.901585506	-0.010541791	0.795179361
UF.PF.2020.003.2G	0.210409441	-0.07819714	0.630392218
UF.PF.2020.004.1N	0.362340353	-0.00102112	0.633022487
UF.PF.2020.004.2G	-0.423798072	0.082451123	0.354598098
UF.PF.2020.005.2G	-0.482443688	-0.03783231	0.438012852
UF.PF.2020.006.2G	-0.162544386	-0.088106506	0.754523353
UF.PF.2020.007.2G	1.151531051	-0.079944415	0.063534785
UF.PF.2020.008.1N	-0.092377239	-0.051395487	-0.45689471
UF.PF.2020.009.3N	0.660737841	0.065544292	-0.368125124
UF.PF.2020.010.1N	-0.063675299	-0.001273388	-0.365521739
UF.PF.2020.011.1N	0.857018757	-0.03444524	-0.280216426
UF.PF.2020.016.1N	-0.603424871	0.083354513	0.085672602
UF.PF.2020.017.1N	-0.501917714	0.089763293	-0.39614846
UF.PF.2020.018.1N	0.71666019	-0.032372956	-0.979026763
UF.PF.2020.021.1N	-0.388732429	0.04120882	0.148584979
UF.PF.2020.022.1N	-0.504068926	0.092122985	0.134455444
UF.PF.2020.023.1N	-0.518574323	0.036792324	0.334026027
UF.PF.2020.024.1N	-0.715231612	0.121214166	-0.601075255
UF.PF.2020.025.3N	-0.277787184	0.150988981	-0.180830323
UF.PF.2020.026.1N	-0.055697876	0.013422048	0.306854427
UF.PF.2020.030.1N	-0.708742056	0.040694613	0.676018129
UF.PF.2021.008.2G	-0.43869048	0.014949871	0.463126046
UF.PF.2021.009.2G	-0.08397974	-0.008530087	0.268193121
UF.PF.2021.010.2G	-0.531261763	0.067953192	-0.424338756
UF.PF.2021.011.2G	-0.446166672	-0.038160241	0.320825983
UF.PF.2021.012.2G	-0.590898839	-0.037740179	0.334475773
UF.PF.2021.014.2G	-0.927271354	-0.042121113	1.477679034
UF.PF.2021.015.2G	0.081971309	0.005485328	0.517113896
UF.PF.2021.016.2G	-0.292641523	0.107855611	0.434360776
UF.PF.2021.017.2G	-0.665482424	-0.010032424	0.230864717
UF.PF.2021.018.2G	0.188075038	0.062040649	0.128512396
UF.PF.2021.019.2G	-0.380108358	0.045909683	0.475204501
UF.PF.2021.020.2G	0.877152297	-0.04390159	-0.669838097
UF.PF.2021.022.2G	0.060334219	0.01675289	0.37747766
UF.PF.2021.023.2G	0.729302254	-0.114555071	-0.379342392

Sample	down	ns	up
UF.PF.2021.024.2G	0.009390702	0.002437934	0.498825096
UF.PF.2021.025.3G	0.022964752	-0.046936322	-1.077766735
UF.PF.2021.031.1N	0.368441445	-0.21179496	-0.510965689
UF.PF.2021.032.3N	-1.020456981	-0.054330093	-0.339694519
UF.PF.2021.033.1N	-0.800919891	0.064361571	0.492165998
UF.PF.2021.034.3N	0.614279849	-0.068826563	0.022365642
UF.PF.2021.035.1N	-0.517943334	0.028173745	-0.566962055
UF.PF.2021.036.3N	0.701955824	0.008714301	-1.167945403
UF.PF.2021.037.1N	0.111464759	0.137696022	-0.251868501
UF.PF.2021.038.3N	-0.606553002	0.156904567	-0.496669685
UF.PF.2021.039.3N	1.041695637	0.03265595	-0.122632675
UF.PF.2021.040.1N	-0.487763841	0.145151226	0.005061937
UF.PF.2021.041.1N	0.098244991	0.07839202	-0.666170426
UF.PF.2021.042.3N	-0.022541553	0.080912121	-0.246324031
UF.PF.2021.044.1N	-0.790166851	-0.084527199	-0.703142563
UF.PF.2021.045.1N	-1.245452173	-0.043766814	1.199796751
UF.PF.2021.046.3N	0.449552315	0.009132371	-1.27235883
UF.PF.2021.047.1N	1.470095397	-0.091724434	-0.711610959
UF.PF.2021.048.1N	-0.059852315	-0.072988189	-0.628482073
UF.PF.2021.049.1N	-1.17435813	-0.126176164	-1.336457994
UF.PF.2021.050.1N	-0.333889348	0.036662119	0.745678837
UF.PF.2021.051.1N	0.477304237	0.090943615	-0.486642849
UF.PF.2021.054.1N	0.427453712	-0.003515219	-0.445736346
UF.PF.2021.055.1N	-1.426020573	-0.254518962	1.281402226
UF.PF.2021.056.3N	1.437209403	0.005098062	-0.382049257
UF.PF.2021.057.1N	0.013221223	-0.017187596	-1.090822936
UF.PF.2021.058.1N	0.154765975	0.007639733	0.053212762
UF.PF.2021.060.3N	1.095546719	0.03764566	-0.134889027
UF.PF.2021.062.1N	0.297338776	0.050822587	-0.812910587
UF.PF.2021.063.1N	-0.275239076	-0.126662395	-0.899843444
UF.PF.2021.064.1N	-0.733114121	0.082274655	-0.062157244
UF.PF.2022.029.2G	0.545005442	-0.03078594	1.002507681
UF.PF.2022.030.2G	1.327021789	-0.062054859	0.039345422
UF.PF.2022.068.1N	-0.665713927	0.062829337	0.957684955
UF.PF.2022.069.1N	0.540746486	0.078683311	1.109331693
UF.PF.2022.070.3N	0.319696218	0.090627809	0.82941321
UF.PF.2022.071.1N	-1.159277048	-0.081108351	-0.003237879
UF.PF.2022.072.1N	0.258098983	0.071399252	-0.239460455
UF.PF.2022.074.1N	-0.310817428	0.15006315	0.386740672
UF.PF.2022.075.1N	-0.316925741	-0.060083373	1.679539987
UF.PF.2022.076.1N	0.116227891	0.049919443	0.350393298
UF.PF.2022.079.1N	0.810104148	0.028754422	-0.813581177
UF.PF.2022.087.1N	0.395532378	0.017773787	0.247165686
UF.PF.2022.088.1N	-0.672133913	0.078753325	-0.071483674
UF.PF.2022.031.2G	0.592949618	0.052262395	-0.435421901
UF.PF.2022.084.1N	-0.575666904	-0.041685129	0.690104501

Sample	down	ns	up
UF.PF.2022.085.3N	-0.150238554	-0.067107321	0.139730125
UF.PF.2022.091.1N	0.650629119	0.000797337	-0.375737519
UF.PF.2022.092.1N	0.215929704	-0.003756164	-0.798581685
UF.PF.2022.096.1N	1.592975499	-0.11836386	-0.457643521
UF.PF.2022.100.1N	-0.179586027	0.06123035	0.210354834
UF.PF.2022.102.1N	0.340967102	0.015915387	0.171207399
UF.PF.2022.103.1N	0.082892364	-0.181273972	0.060449122
UF.PF.2023.032.2G	-0.797102252	-0.21077441	0.187110611
UF.PF.2023.119.1N	-0.266756586	0.037362733	0.745929627
UF.PF.2023.123.1N	0.300317383	-0.175962864	-0.428420653
UF.PF.2023.124.3N	0.635503923	-0.111751744	0.449272261

WGCNA Module List - UFPF Dataset (Pathways)

Below is the module list extracted from our WGCNA analysis (of the UFPF dataset) displaying specific microbial pathways and their module assignments- ns (not-significant), down, or up.

Pathway	Module
1CMET2-PWY: folate transformations III (E. coli)	down
ALLANTOINDEG-PWY: superpathway of allantoin degradation in yeast	ns
ANAEROFRUCAT-PWY: homolactic fermentation	down
ANAGLYCOLYSIS-PWY: glycolysis III (from glucose)	down
ARG+POLYAMINE-SYN: superpathway of arginine and polyamine biosynthesis	down
ARGDEG-PWY: superpathway of L-arginine, putrescine, and 4-aminobutanoate degradation	up
ARGININE-SYN4-PWY: L-ornithine biosynthesis II	down
ARGSYN-PWY: L-arginine biosynthesis I (via L-ornithine)	down
ARGSYNBSUB-PWY: L-arginine biosynthesis II (acetyl cycle)	down
ARO-PWY: chorismate biosynthesis I	down
ASPASN-PWY: superpathway of L-aspartate and L-asparagine biosynthesis	down
AST-PWY: L-arginine degradation II (AST pathway)	ns
BIOTIN-BIOSYNTHESIS-PWY: biotin biosynthesis I	ns
BRANCHED-CHAIN-AA-SYN-PWY: superpathway of branched chain amino acid biosynthesis	down
CALVIN-PWY: Calvin-Benson-Bassham cycle	down
CENTFERM-PWY: pyruvate fermentation to butanoate	ns
CITRULBIO-PWY: L-citrulline biosynthesis	down
COA-PWY-1: superpathway of coenzyme A biosynthesis III (mammals)	down
COA-PWY: coenzyme A biosynthesis I (prokaryotic)	down
COBALSYN-PWY: superpathway of adenosylcobalamin salvage from cobinamide I	down
COLANSYN-PWY: colanic acid building blocks biosynthesis	down
COMPLETE-ARO-PWY: superpathway of aromatic amino acid biosynthesis	down
DAPLYSINESYN-PWY: L-lysine biosynthesis I	ns
DARABCATK12-PWY: D-arabinose degradation I	ns
DTDPRHAMSYN-PWY: dTDP-β-L-rhamnose biosynthesis	down
ECASYN-PWY: enterobacterial common antigen biosynthesis	ns
FAO-PWY: fatty acid β-oxidation I (generic)	ns
FASYN-ELONG-PWY: fatty acid elongation -- saturated	ns
FERMENTATION-PWY: mixed acid fermentation	ns
FOLSYN-PWY: superpathway of tetrahydrofolate biosynthesis and salvage	up
FUC-RHAMCAT-PWY: superpathway of fucose and rhamnose degradation	ns
FUCCAT-PWY: fucose degradation	down
GALACT-GLUCUROCAT-PWY: superpathway of hexuronide and hexuronate degradation	down
GALACTARDEG-PWY: D-galactarate degradation I	ns

Pathway	Module
GALACTITOLCAT-PWY: galactitol degradation	ns
GALACTUROCAT-PWY: D-galacturonate degradation I	down
GLCMANNANAUT-PWY: superpathway of N-acetylglucosamine, N-acetylmannosamine and N-acetylneuraminate degradation	down
GLUCARDEG-PWY: D-glucarate degradation I	ns
GLUCARGALACTSUPER-PWY: superpathway of D-glucarate and D-galactarate degradation	ns
GLUCONEO-PWY: gluconeogenesis I	down
GLUCOSE1PMETAB-PWY: glucose and glucose-1-phosphate degradation	up
GLUCUROCAT-PWY: superpathway of β-D-glucuronosides degradation	down
GLUDEG-I-PWY: GABA shunt	ns
GLUTORN-PWY: L-ornithine biosynthesis I	down
GLYCOCAT-PWY: glycogen degradation I	up
GLYCOGENSYNTH-PWY: glycogen biosynthesis I (from ADP-D-Glucose)	down
GLYCOL-GLYOXDEG-PWY: superpathway of glycol metabolism and degradation	ns
GLYCOLYSIS-E-D: superpathway of glycolysis and the Entner-Doudoroff pathway	down
GLYCOLYSIS-TCA-GLYOX-BYPASS: superpathway of glycolysis, pyruvate dehydrogenase, TCA, and glyoxylate bypass	ns
GLYCOLYSIS: glycolysis I (from glucose 6-phosphate)	ns
GLYOXYLATE-BYPASS: glyoxylate cycle	ns
GOLPDLCAT-PWY: superpathway of glycerol degradation to 1,3-propanediol	down
HCAMHPDEG-PWY: 3-phenylpropanoate and 3-(3-hydroxyphenyl)propanoate degradation to 2-hydroxypentadienoate	ns
HEME-BIOSYNTHESIS-II-1: heme b biosynthesis V (aerobic)	ns
HEME-BIOSYNTHESIS-II: heme b biosynthesis I (aerobic)	ns
HEMESYN2-PWY: heme b biosynthesis II (oxygen-independent)	ns
HEXITOLDEGSUPER-PWY: superpathway of hexitol degradation (bacteria)	down
HISDEG-PWY: L-histidine degradation I	down
HISTSYN-PWY: L-histidine biosynthesis	down
HOMOSER-METSYN-PWY: L-methionine biosynthesis I	ns
HSERMETANA-PWY: L-methionine biosynthesis III	down
ILEUSYN-PWY: L-isoleucine biosynthesis I (from threonine)	down
KDO-NAGLIPASYN-PWY: superpathway of (Kdo)2-lipid A biosynthesis	ns
KETOGLUCONMET-PWY: ketogluconate metabolism	up
LACTOSECAT-PWY: lactose and galactose degradation I	ns
LIPASYN-PWY: phospholipases	ns
MET-SAM-PWY: superpathway of S-adenosyl-L-methionine biosynthesis	down
METH-ACETATE-PWY: methanogenesis from acetate	down
METHGLYUT-PWY: superpathway of methylglyoxal degradation	ns
METSYN-PWY: superpathway of L-homoserine and L-methionine biosynthesis	down

Pathway	Module
NAD-BIOSYNTHESIS-II: NAD salvage pathway III (to nicotinamide riboside)	ns
NAGLIPASYN-PWY: lipid IVA biosynthesis (E. coli)	ns
NONMEVIPP-PWY: methylerythritol phosphate pathway I	down
NONOXIPENT-PWY: pentose phosphate pathway (non-oxidative branch) I	down
OANTIGEN-PWY: O-antigen building blocks biosynthesis (E. coli)	down
ORNARGDEG-PWY: superpathway of L-arginine and L-ornithine degradation	up
ORNDEG-PWY: superpathway of ornithine degradation	up
P105-PWY: TCA cycle IV (2-oxoglutarate decarboxylase)	ns
P108-PWY: pyruvate fermentation to propanoate I	ns
P122-PWY: heterolactic fermentation	up
P124-PWY: Bifidobacterium shunt	up
P161-PWY: acetylene degradation (anaerobic)	up
P164-PWY: purine nucleobases degradation I (anaerobic)	down
P185-PWY: formaldehyde assimilation III (dihydroxyacetone cycle)	down
P221-PWY: octane oxidation	up
P23-PWY: reductive TCA cycle I	up
P4-PWY: superpathway of L-lysine, L-threonine and L-methionine biosynthesis I	ns
P41-PWY: pyruvate fermentation to acetate and (S)-lactate I	ns
P42-PWY: incomplete reductive TCA cycle	ns
P441-PWY: superpathway of N-acetylneuraminate degradation	ns
P461-PWY: hexitol fermentation to lactate, formate, ethanol and acetate	ns
PANTO-PWY: phosphopantothenate biosynthesis I	down
PANTOSYN-PWY: superpathway of coenzyme A biosynthesis I (bacteria)	down
PENTOSE-P-PWY: pentose phosphate pathway	down
PEPTIDOGLYCANSYN-PWY: peptidoglycan biosynthesis I (meso-diaminopimelate containing)	down
PHOSLIPSYN-PWY: superpathway of phospholipid biosynthesis I (bacteria)	down
POLYAMINSYN3-PWY: superpathway of polyamine biosynthesis II	ns
POLYAMSYN-PWY: superpathway of polyamine biosynthesis I	down
POLYISOPRENSYN-PWY: polyisoprenoid biosynthesis (E. coli)	down
PPGPPMET-PWY: ppGpp metabolism	ns
PWY-1042: glycolysis IV	down
PWY-1269: CMP-3-deoxy-D-manno-octulosonate biosynthesis	down
PWY-1861: formaldehyde assimilation II (assimilatory RuMP Cycle)	ns
PWY-241: C4 photosynthetic carbon assimilation cycle, NADP-ME type	ns
PWY-2941: L-lysine biosynthesis II	ns
PWY-2942: L-lysine biosynthesis III	down
PWY-3001: superpathway of L-isoleucine biosynthesis I	down
PWY-3841: folate transformations II (plants)	down
PWY-4041: γ-glutamyl cycle	ns
PWY-4984: urea cycle	down
PWY-5004: superpathway of L-citrulline metabolism	ns

Pathway	Module
PWY-5005: biotin biosynthesis II	down
PWY-5022: 4-aminobutanoate degradation V	ns
PWY-5030: L-histidine degradation III	down
PWY-5097: L-lysine biosynthesis VI	down
PWY-5100: pyruvate fermentation to acetate and lactate II	down
PWY-5103: L-isoleucine biosynthesis III	down
PWY-5104: L-isoleucine biosynthesis IV	down
PWY-5121: superpathway of geranylgeranyl diphosphate biosynthesis II (via MEP)	down
PWY-5130: 2-oxobutanoate degradation I	ns
PWY-5136: fatty acid β-oxidation II (plant peroxisome)	ns
PWY-5138: fatty acid β-oxidation IV (unsaturated, even number)	ns
PWY-5154: L-arginine biosynthesis III (via N-acetyl-L-citrulline)	down
PWY-5188: tetrapyrrole biosynthesis I (from glutamate)	down
PWY-5189: tetrapyrrole biosynthesis II (from glycine)	ns
PWY-5345: superpathway of L-methionine biosynthesis (by sulfhydrylation)	ns
PWY-5347: superpathway of L-methionine biosynthesis (transsulfuration)	down
PWY-5367: petroselinate biosynthesis	ns
PWY-5384: sucrose degradation IV (sucrose phosphorylase)	ns
PWY-5484: glycolysis II (from fructose 6-phosphate)	ns
PWY-5497: purine nucleobases degradation II (anaerobic)	ns
PWY-5505: L-glutamate and L-glutamine biosynthesis	down
PWY-561: superpathway of glyoxylate cycle and fatty acid degradation	ns
PWY-5656: mannosylglycerate biosynthesis I	up
PWY-5659: GDP-mannose biosynthesis	down
PWY-5667: CDP-diacylglycerol biosynthesis I	down
PWY-5675: nitrate reduction V (assimilatory)	ns
PWY-5676: acetyl-CoA fermentation to butanoate II	down
PWY-5686: UMP biosynthesis I	down
PWY-5690: TCA cycle II (plants and fungi)	ns
PWY-5692: allantoin degradation to glyoxylate II	ns
PWY-5695: inosine 5'-phosphate degradation	down
PWY-5705: allantoin degradation to glyoxylate III	ns
PWY-5723: Rubisco shunt	ns
PWY-5747: 2-methylcitrate cycle II	ns
PWY-5837: 2-carboxy-1,4-naphthoquinol biosynthesis	up
PWY-5838: superpathway of menaquinol-8 biosynthesis I	up
PWY-5840: superpathway of menaquinol-7 biosynthesis	ns
PWY-5845: superpathway of menaquinol-9 biosynthesis	up
PWY-5850: superpathway of menaquinol-6 biosynthesis	ns
PWY-5855: ubiquinol-7 biosynthesis (early decarboxylation)	ns
PWY-5860: superpathway of demethylmenaquinol-6 biosynthesis I	ns
PWY-5861: superpathway of demethylmenaquinol-8 biosynthesis I	up
PWY-5862: superpathway of demethylmenaquinol-9 biosynthesis	up

Pathway	Module
PWY-5896: superpathway of menaquinol-10 biosynthesis	ns
PWY-5897: superpathway of menaquinol-11 biosynthesis	up
PWY-5898: superpathway of menaquinol-12 biosynthesis	up
PWY-5899: superpathway of menaquinol-13 biosynthesis	up
PWY-5913: partial TCA cycle (obligate autotrophs)	ns
PWY-5918: superpathway of heme b biosynthesis from glutamate	ns
PWY-5920: superpathway of heme b biosynthesis from glycine	ns
PWY-5941: glycogen degradation II	ns
PWY-5971: palmitate biosynthesis (type II fatty acid synthase)	ns
PWY-5973: cis-vaccenate biosynthesis	down
PWY-5981: CDP-diacylglycerol biosynthesis III	ns
PWY-5989: stearate biosynthesis II (bacteria and plants)	ns
PWY-6121: 5-aminoimidazole ribonucleotide biosynthesis I	down
PWY-6122: 5-aminoimidazole ribonucleotide biosynthesis II	down
PWY-6123: inosine-5'-phosphate biosynthesis I	down
PWY-6124: inosine-5'-phosphate biosynthesis II	down
PWY-6125: superpathway of guanosine nucleotides de novo biosynthesis II	down
PWY-6126: superpathway of adenosine nucleotides de novo biosynthesis II	down
PWY-6147: 6-hydroxymethyl-dihydropterin diphosphate biosynthesis I	down
PWY-6151: S-adenosyl-L-methionine salvage I	down
PWY-6163: chorismate biosynthesis from 3-dehydroquinate	down
PWY-6168: flavin biosynthesis III (fungi)	down
PWY-621: sucrose degradation III (sucrose invertase)	ns
PWY-6270: isoprene biosynthesis I	down
PWY-6277: superpathway of 5-aminoimidazole ribonucleotide biosynthesis	down
PWY-6282: palmitoleate biosynthesis I (from (5Z)-dodec-5-enoate)	ns
PWY-6284: superpathway of unsaturated fatty acids biosynthesis (E. coli)	ns
PWY-6285: superpathway of fatty acids biosynthesis (E. coli)	up
PWY-6292: superpathway of L-cysteine biosynthesis (mammalian)	ns
PWY-6293: superpathway of L-cysteine biosynthesis (fungi)	ns
PWY-6305: superpathway of putrescine biosynthesis	down
PWY-6317: D-galactose degradation I (Leloir pathway)	down
PWY-6318: L-phenylalanine degradation IV (mammalian, via side chain)	ns
PWY-6328: L-lysine degradation X	ns
PWY-6353: purine nucleotides degradation II (aerobic)	down
PWY-6385: peptidoglycan biosynthesis III (mycobacteria)	down
PWY-6386: UDP-N-acetylmuramoyl-pentapeptide biosynthesis II (lysine-containing)	down
PWY-6387: UDP-N-acetylmuramoyl-pentapeptide biosynthesis I (meso-diaminopimelate containing)	down
PWY-6470: peptidoglycan biosynthesis V (β-lactam resistance)	down
PWY-6507: 4-deoxy-L-threo-hex-4-enopyranuronate degradation	down

Pathway	Module
PWY-6519: 8-amino-7-oxononanoate biosynthesis I	ns
PWY-6527: stachyose degradation	down
PWY-6531: mannitol cycle	ns
PWY-6545: pyrimidine deoxyribonucleotides de novo biosynthesis III	ns
PWY-6549: L-glutamine biosynthesis III	ns
PWY-6588: pyruvate fermentation to acetone	ns
PWY-6590: superpathway of Clostridium acetobutylicum acidogenic fermentation	ns
PWY-6595: superpathway of guanosine nucleotides degradation (plants)	up
PWY-6606: guanosine nucleotides degradation II	down
PWY-6607: guanosine nucleotides degradation I	ns
PWY-6608: guanosine nucleotides degradation III	down
PWY-6609: adenine and adenosine salvage III	down
PWY-6612: superpathway of tetrahydrofolate biosynthesis	up
PWY-6628: superpathway of L-phenylalanine biosynthesis	ns
PWY-6630: superpathway of L-tyrosine biosynthesis	ns
PWY-6690: cinnamate and 3-hydroxycinnamate degradation to 2-hydroxypentadienoate	ns
PWY-6700: queuosine biosynthesis I (de novo)	down
PWY-6703: preQ0 biosynthesis	down
PWY-6708: ubiquinol-8 biosynthesis (early decarboxylation)	ns
PWY-6731: starch degradation III	up
PWY-6749: CMP-legionaminate biosynthesis I	ns
PWY-6803: phosphatidylcholine acyl editing	ns
PWY-6823: molybdopterin biosynthesis	ns
PWY-6859: all-trans-farnesol biosynthesis	ns
PWY-6895: superpathway of thiamine diphosphate biosynthesis II	ns
PWY-6897: thiamine diphosphate salvage II	down
PWY-6901: superpathway of glucose and xylose degradation	ns
PWY-6902: chitin degradation II (Vibrio)	ns
PWY-6906: chitin derivatives degradation	ns
PWY-6922: L-Nδ-acetylornithine biosynthesis	ns
PWY-6936: seleno-amino acid biosynthesis (plants)	down
PWY-6961: L-ascorbate degradation II (bacterial, aerobic)	ns
PWY-6969: TCA cycle V (2-oxoglutarate synthase)	ns
PWY-6992: 1,5-anhydrofructose degradation	ns
PWY-7013: (S)-propane-1,2-diol degradation	ns
PWY-702: L-methionine biosynthesis II	ns
PWY-7094: fatty acid salvage	ns
PWY-7111: pyruvate fermentation to isobutanol (engineered)	down
PWY-7115: C4 photosynthetic carbon assimilation cycle, NAD-ME type	ns
PWY-7117: C4 photosynthetic carbon assimilation cycle, PEPCK type	ns
PWY-7118: chitin deacetylation	ns
PWY-7184: pyrimidine deoxyribonucleotides de novo biosynthesis I	ns
PWY-7197: pyrimidine deoxyribonucleotide phosphorylation	down

Pathway	Module
PWY-7198: pyrimidine deoxyribonucleotides de novo biosynthesis IV	up
PWY-7199: pyrimidine deoxyribonucleosides salvage	down
PWY-7204: pyridoxal 5'-phosphate salvage II (plants)	ns
PWY-7208: superpathway of pyrimidine nucleobases salvage	down
PWY-7209: superpathway of pyrimidine ribonucleosides degradation	up
PWY-7210: pyrimidine deoxyribonucleotides biosynthesis from CTP	ns
PWY-7211: superpathway of pyrimidine deoxyribonucleotides de novo biosynthesis	ns
PWY-7220: adenosine deoxyribonucleotides de novo biosynthesis II	down
PWY-7221: guanosine ribonucleotides de novo biosynthesis	down
PWY-7222: guanosine deoxyribonucleotides de novo biosynthesis II	down
PWY-7228: superpathway of guanosine nucleotides de novo biosynthesis I	down
PWY-7229: superpathway of adenosine nucleotides de novo biosynthesis I	down
PWY-7234: inosine-5'-phosphate biosynthesis III	ns
PWY-7237: myo-, chiro- and scyllo-inositol degradation	down
PWY-7238: sucrose biosynthesis II	ns
PWY-724: superpathway of L-lysine, L-threonine and L-methionine biosynthesis II	down
PWY-7242: D-fructuronate degradation	down
PWY-7254: TCA cycle VII (acetate-producers)	ns
PWY-7269: mitochondrial NADPH production (yeast)	ns
PWY-7282: 4-amino-2-methyl-5-diphosphomethylpyrimidine biosynthesis II	down
PWY-7315: dTDP-N-acetylthomosamine biosynthesis	ns
PWY-7323: superpathway of GDP-mannose-derived O-antigen building blocks biosynthesis	down
PWY-7328: superpathway of UDP-glucose-derived O-antigen building blocks biosynthesis	up
PWY-7345: superpathway of anaerobic sucrose degradation	ns
PWY-7356: thiamine diphosphate salvage IV (yeast)	ns
PWY-7357: thiamine phosphate formation from pyrithiamine and oxythiamine (yeast)	down
PWY-7383: anaerobic energy metabolism (invertebrates, cytosol)	ns
PWY-7385: 1,3-propanediol biosynthesis (engineered)	ns
PWY-7388: octanoyl-[acyl-carrier protein] biosynthesis (mitochondria, yeast)	ns
PWY-7392: taxadiene biosynthesis (engineered)	ns
PWY-7400: L-arginine biosynthesis IV (archaebacteria)	down
PWY-7409: phospholipid remodeling (phosphatidylethanolamine, yeast)	ns
PWY-7456: β-(1,4)-mannan degradation	down
PWY-7560: methylerythritol phosphate pathway II	down
PWY-7616: methanol oxidation to carbon dioxide	ns
PWY-7663: gondoate biosynthesis (anaerobic)	down

Pathway	Module
PWY-7664: oleate biosynthesis IV (anaerobic)	ns
PWY-7761: NAD salvage pathway II (PNC IV cycle)	ns
PWY-7790: UMP biosynthesis II	ns
PWY-7791: UMP biosynthesis III	ns
PWY-7805: (aminomethyl)phosphonate degradation	ns
PWY-7807: glyphosate degradation III	ns
PWY-7851: coenzyme A biosynthesis II (eukaryotic)	ns
PWY-7858: (5Z)-dodecenoate biosynthesis II	ns
PWY-7874: L-threonate degradation	ns
PWY-7883: anhydromuropeptides recycling II	ns
PWY-7942: 5-oxo-L-proline metabolism	ns
PWY-7953: UDP-N-acetylmuramoyl-pentapeptide biosynthesis III (meso-diaminopimelate containing)	ns
PWY-7977: L-methionine biosynthesis IV	ns
PWY-8004: Entner-Doudoroff pathway I	ns
PWY-801: homocysteine and cysteine interconversion	ns
PWY-8073: lipid IVA biosynthesis (P. putida)	ns
PWY-8131: 5'-deoxyadenosine degradation II	ns
PWY-8178: pentose phosphate pathway (non-oxidative branch) II	ns
PWY-8187: L-arginine degradation XIII (reductive Stickland reaction)	ns
PWY-821: superpathway of sulfur amino acid biosynthesis (Saccharomyces cerevisiae)	up
PWY-841: superpathway of purine nucleotides de novo biosynthesis I	down
PWY-I9: L-cysteine biosynthesis VI (from L-methionine)	ns
PWY0-1061: superpathway of L-alanine biosynthesis	ns
PWY0-1241: ADP-L-glycero-β-D-manno-heptose biosynthesis	ns
PWY0-1261: anhydromuropeptides recycling I	down
PWY0-1277: 3-phenylpropanoate and 3-(3-hydroxyphenyl)propanoate degradation	ns
PWY0-1296: purine ribonucleosides degradation	down
PWY0-1297: superpathway of purine deoxyribonucleosides degradation	ns
PWY0-1298: superpathway of pyrimidine deoxyribonucleosides degradation	ns
PWY0-1319: CDP-diacylglycerol biosynthesis II	down
PWY0-1337: oleate β-oxidation	ns
PWY0-1338: polymyxin resistance	ns
PWY0-1415: superpathway of heme b biosynthesis from uroporphyrinogen-III	ns
PWY0-1477: ethanolamine utilization	ns
PWY0-1479: tRNA processing	ns
PWY0-1533: methylphosphonate degradation I	ns
PWY0-1586: peptidoglycan maturation (meso-diaminopimelate containing)	down
PWY0-162: superpathway of pyrimidine ribonucleotides de novo biosynthesis	down

Pathway	Module
PWY0-166: superpathway of pyrimidine deoxyribonucleotides de novo biosynthesis (E. coli)	up
PWY0-301: L-ascorbate degradation I (bacterial, anaerobic)	ns
PWY0-41: allantoin degradation IV (anaerobic)	ns
PWY0-42: 2-methylcitrate cycle I	ns
PWY0-461: L-lysine degradation I	ns
PWY0-781: aspartate superpathway	ns
PWY0-845: superpathway of pyridoxal 5'-phosphate biosynthesis and salvage	ns
PWY0-862: (5Z)-dodecenoate biosynthesis I	ns
PWY1G-0: mycothiol biosynthesis	ns
PWY1ZNC-1: assimilatory sulfate reduction IV	ns
PWY3O-4107: NAD salvage pathway V (PNC V cycle)	ns
PWY4FS-7: phosphatidylglycerol biosynthesis I (plastidic)	down
PWY4FS-8: phosphatidylglycerol biosynthesis II (non-plastidic)	down
PWY4LZ-257: superpathway of fermentation (Chlamydomonas reinhardtii)	up
PWY66-389: phytol degradation	up
PWY66-391: fatty acid β-oxidation VI (mammalian peroxisome)	ns
PWY66-399: gluconeogenesis III	ns
PWY66-409: superpathway of purine nucleotide salvage	ns
PWY66-429: fatty acid biosynthesis initiation (mitochondria)	ns
PWY66-430: myristate biosynthesis (mitochondria)	ns
PYRIDNUCSAL-PWY: NAD salvage pathway I (PNC VI cycle)	ns
PYRIDNUCSYN-PWY: NAD de novo biosynthesis I (from aspartate)	down
PYRIDOXSYN-PWY: pyridoxal 5'-phosphate biosynthesis I	down
REDCITCYC: TCA cycle VI (Helicobacter)	up
RHAMCAT-PWY: L-rhamnose degradation I	down
RIBOSYN2-PWY: flavin biosynthesis I (bacteria and plants)	down
SALVADEHYPOX-PWY: adenosine nucleotides degradation II	down
SER-GLYSYN-PWY: superpathway of L-serine and glycine biosynthesis I	down
SO4ASSIM-PWY: assimilatory sulfate reduction I	ns
SULFATE-CYS-PWY: superpathway of sulfate assimilation and cysteine biosynthesis	ns
TCA-GLYOX-BYPASS: superpathway of glyoxylate bypass and TCA	ns
TCA: TCA cycle I (prokaryotic)	ns
THISYNARA-PWY: superpathway of thiamine diphosphate biosynthesis III (eukaryotes)	down
THRESYN-PWY: superpathway of L-threonine biosynthesis	down
TRNA-CHARGING-PWY: tRNA charging	down
UBISYN-PWY: superpathway of ubiquinol-8 biosynthesis (early decarboxylation)	ns
UDPNAGSYN-PWY: UDP-N-acetyl-D-glucosamine biosynthesis I	ns
URDEGR-PWY: superpathway of allantoin degradation in plants	ns
VALSYN-PWY: L-valine biosynthesis	down

WGCNA Module Scores - UFPF Dataset (Pathways)

Module scores refer to the average abundance of microbial pathways in each module. Module scores are represented by the module eigengene, the first principal component of the pathways in each module and represents the overall abundance or expression trend of that module across samples.

Sample	down	ns	up
UF.PF.2020.001.1N	0.193727	-0.03396	-0.47865
UF.PF.2020.003.1N	-0.95483	-0.00877	0.344945
UF.PF.2020.004.1N	0.492343	-0.24367	-0.75
UF.PF.2020.008.1N	-0.32266	-0.10973	-0.17477
UF.PF.2020.010.1N	-0.78118	-0.07172	0.24877
UF.PF.2020.011.1N	0.543696	-0.22805	-0.81642
UF.PF.2020.016.1N	-0.16382	-0.16083	0.006885
UF.PF.2020.017.1N	0.179086	-0.11155	-0.20219
UF.PF.2020.018.1N	-0.30019	-0.15304	-0.08348
UF.PF.2020.021.1N	0.478029	-0.32166	-0.37489
UF.PF.2020.022.1N	-0.45457	-0.09212	0.311536
UF.PF.2020.024.1N	0.191939	-0.1604	-0.19066
UF.PF.2020.026.1N	0.134689	-0.20622	-0.56601
UF.PF.2020.030.1N	-0.84625	-0.02668	0.311902
UF.PF.2021.031.1N	0.408042	-0.38125	-0.10755
UF.PF.2021.033.1N	0.218704	-0.1097	-0.54975
UF.PF.2021.035.1N	-0.03971	-0.2457	0.016324
UF.PF.2021.037.1N	-0.07416	-0.1654	0.067651
UF.PF.2021.040.1N	-0.3304	0.006811	-0.1062
UF.PF.2021.041.1N	0.126807	-0.2057	-0.20505
UF.PF.2021.044.1N	0.337262	-0.27527	-0.33931
UF.PF.2021.045.1N	-0.28762	-0.21627	-0.05124
UF.PF.2021.047.1N	0.707474	-0.34806	-0.92478
UF.PF.2021.048.1N	0.141108	-0.27549	-0.52193
UF.PF.2021.049.1N	0.65925	-0.364	-0.1387
UF.PF.2021.050.1N	0.353203	-0.2968	-0.15246
UF.PF.2021.051.1N	0.133515	-0.31004	-0.20926
UF.PF.2021.054.1N	0.761923	-0.31737	-0.75389
UF.PF.2021.055.1N	-0.47039	-0.16947	0.035621
UF.PF.2021.057.1N	0.764561	-0.43493	-0.39396
UF.PF.2021.058.1N	0.586867	-0.26118	-0.67386
UF.PF.2021.062.1N	0.689171	-0.34525	-0.45751
UF.PF.2021.063.1N	-0.08496	-0.30108	0.129815
UF.PF.2021.064.1N	-0.67213	-0.04859	0.059355
UF.PF.2022.068.1N	-0.82023	-0.02816	0.176155
UF.PF.2022.069.1N	0.075931	-0.0706	-0.32821
UF.PF.2022.071.1N	-0.24616	-0.20737	-0.17321
UF.PF.2022.072.1N	0.224892	-0.19047	-0.74075

Sample	down	ns	up
UF.PF.2022.074.1N	-0.93862	0.116527	0.093768
UF.PF.2022.075.1N	0.476871	-0.27588	-0.55942
UF.PF.2022.076.1N	-0.84174	-0.01217	0.170411
UF.PF.2022.079.1N	0.311654	-0.20039	-0.50832
UF.PF.2022.084.1N	-0.56883	-0.06732	-0.02546
UF.PF.2022.087.1N	0.737037	-0.36113	-0.61533
UF.PF.2022.088.1N	-0.00316	-0.16469	-0.15697
UF.PF.2022.091.1N	0.610731	-0.30813	-0.55698
UF.PF.2022.092.1N	0.786139	-0.36316	-0.37091
UF.PF.2022.096.1N	1.053411	-0.47469	-0.97165
UF.PF.2022.100.1N	-1.02136	0.059105	0.085779
UF.PF.2022.102.1N	-1.1802	0.041944	0.428382
UF.PF.2022.103.1N	-0.96164	0.000882	0.102815
UF.PF.2023.119.1N	0.091573	-0.21734	-0.21091
UF.PF.2023.123.1N	0.599466	-0.41377	-0.27836
UF.PF.2020.002.3N	-0.06536	-0.09956	-0.14996
UF.PF.2020.009.3N	-0.18343	-0.10815	0.021727
UF.PF.2020.025.3N	-1.70738	0.230948	0.522574
UF.PF.2021.025.3G	0.624496	-0.18973	-0.79196
UF.PF.2021.032.3N	-0.26707	-0.04688	0.013556
UF.PF.2021.034.3N	0.30685	-0.20185	-0.17377
UF.PF.2021.036.3N	0.660441	-0.1988	-0.59806
UF.PF.2021.038.3N	0.11397	-0.05604	-0.12435
UF.PF.2021.039.3N	0.661427	-0.1398	-0.45285
UF.PF.2021.042.3N	0.053883	-0.06444	0.013172
UF.PF.2021.046.3N	-0.07426	-0.16779	0.436319
UF.PF.2021.056.3N	-0.25057	-0.03868	-0.14479
UF.PF.2021.060.3N	-0.35004	-0.05547	-0.07847
UF.PF.2022.070.3N	0.403688	-0.05069	-0.41535
UF.PF.2022.085.3N	0.065928	-0.0744	-0.63887
UF.PF.2023.124.3N	-0.82017	0.0606	0.16891
UF.PF.2020.001.2G	-0.0267	0.361467	0.659517
UF.PF.2020.002.2G	1.223965	0.186034	-0.10711
UF.PF.2020.003.2G	0.896754	0.141601	0.456296
UF.PF.2020.004.2G	-0.20852	0.506476	0.670543
UF.PF.2020.005.2G	-0.12321	0.509619	0.596393
UF.PF.2020.006.2G	-0.35523	0.448527	0.59981
UF.PF.2020.007.2G	-0.39902	0.515632	0.633404
UF.PF.2021.008.2G	-0.30085	0.548925	0.679633
UF.PF.2021.009.2G	-0.39921	0.507279	0.732429
UF.PF.2021.010.2G	1.319943	0.121302	-0.05158
UF.PF.2021.011.2G	-0.25396	0.52574	0.82465
UF.PF.2021.012.2G	-0.25092	0.5199	0.738199

Sample	down	ns	up
UF.PF.2021.014.2G	0.186327	0.331018	0.379772
UF.PF.2021.015.2G	-0.25136	0.603539	0.651633
UF.PF.2021.016.2G	-0.24223	0.557799	0.489432
UF.PF.2021.017.2G	-0.26113	0.484423	0.677468
UF.PF.2021.018.2G	0.143772	0.35116	0.587049
UF.PF.2021.019.2G	-0.35052	0.563602	0.767143
UF.PF.2021.020.2G	0.481049	0.405717	0.300255
UF.PF.2021.022.2G	-0.2361	0.539215	0.686435
UF.PF.2021.023.2G	-0.31153	0.414283	0.579485
UF.PF.2021.024.2G	-0.36951	0.564642	0.811173
UF.PF.2022.029.2G	-0.00273	0.428186	0.437283
UF.PF.2022.030.2G	-0.29539	0.437434	0.726633
UF.PF.2022.031.2G	0.92151	0.281245	0.220239
UF.PF.2023.032.2G	-0.41191	0.475894	0.774842

A comparative analysis of Parkinson’s disease and inflammatory bowel disease gut microbiomes highlights shared depletions in key butyrate-producing bacteria

Maeve E. Krueger, Jake Sondag Boles, Zachary D. Simon, Stephan D. Alvarez, Nikolaus R. McFarland,Michael S. Okun, Ellen M. Zimmermann, Christopher E. Forsmark, Malú Gámez Tansey*
University of Florida

Version: 1.03

Number	Item	Recommendation	Item Source	Additional Guidance	Yes/No/NA	Comments or location in manuscript
**Abstract**						
1.0	Structured or Unstructured Abstract	Abstract should include information on background, methods, results, and conclusions in structured or unstructured format.	STORMS		Yes	pg 2
1.1	Study Design	State study design in abstract.	STORMS	See 3.0 for additional information on study design.	Yes	
1.2	Sequencing methods	State the strategy used for metagenomic classification.	STORMS	For example, targeted 16S by qPCR or sequencing, shotgun metagenomics, metatranscriptomics, etc.	Yes	shallow shotgun metagenomic sequencing
1.3	Specimens	Describe body site(s) studied.	STORMS		Yes	
**Introduction**						
2.0	Background and Rationale	Summarize the underlying background, scientific evidence, or theory driving the current hypothesis as well as the study objectives.	STORMS		Yes	
2.1	Hypotheses	State the pre-specified hypothesis. If the study is exploratory, state any pre-specified study objectives.	STORMS		Yes	
**Methods**						
3.0	Study Design	Describe the study design.	STORMS	Observational (Case-Control, Cohort, Cross-sectional survey, etc.) or Experimental (Randomized controlled trial, Non-randomized controlled trial, etc.). For a brief description of common study designs see: DOI: 10.11613/BM.2014.022<br><br>If applicable, describe any blinding (e.g. single or double-blinding) used in the course of the study.	Yes	Methods
3.1	Participants	State what the population of interest is, and the method by which participants are sampled from that population. Include relevant information on physiological state of the subjects or stage in the life history of disease under study when participants were sampled.	STORMS	Examples of the population of interest could be: adults with no chronic health conditions, adults with type II diabetes, newborns, etc. This is the total population to whom the study is hoped to be generalizable to. The sampling method describes how potential participants were selected from that population.<br><br>If the participants are from a substudy of a larger study, provide a brief description of that study and cite that study.<br><br>Clearly state how cases and controls are defined.<br><br>An example of relevant physiological state might be pre/post menopausal for a vaginal microbiome study; examples of stage in the life history of disease could be whether specimens were collected during active or dormant disease, or before or after treatment.	Yes	Methods
3.2	Geographic location	State the geographic region(s) where participants were sampled from.	MIxS: geographic location (country and/or sea,region)	Geographic coordinates can be reported to prevent potential ambiguities if necessary.	Yes	Methods - Florida
3.3	Relevant Dates	State the start and end dates for recruitment, follow-up, and data collection.	STORMS	Recruitment is the period in which participants are recruited for the study. In longitudinal studies, follow-up is the date range in which participants are asked to complete a specific assessment. Finally, data collection is the total period in which data is being collected from participants including during initial recruitment through all follow-ups.	Yes	Methods - recruitment from August 2020 and May 2023
3.4	Eligibility criteria	List any criteria for inclusion and exclusion of recruited participants.	Modified STROBE	Among potential recruited participants, how were some chosen and others not? This could include criteria such as sex, diet, age, health status, or BMI.<br><br>If there is a primary and validation sample, describe inclusion/exclusion criteria for each.	Yes	Methods

Number	Item	Recommendation	Item Source	Additional Guidance	Yes/No/NA	Comments or location in manuscript
3.5	Antibiotics Usage	List what is known about antibiotics usage before or during sample collection.	STORMS	If participants were excluded due to current or recent antibiotics usage, state this here.<br><br>Other factors (e.g. proton pump inhibitors, probiotics, etc.) that may influence the microbiome should also be described as well.	Yes	Methods - no antibiotic use within 1 month of recruitment
3.6	Analytic sample size	Explain how the final analytic sample size was calculated, including the number of cases and controls if relevant, and reasons for dropout at each stage of the study. This should include the number of individuals in whom microbiome sequencing was attempted and the number in whom microbiome sequencing was successful.	STORMS	Consider use of a flow diagram (see template at https://stormsmicrobiome.org/figures). Also state sample size in abstract.<br><br>If power analysis was used to calculate sample size, describe those calculations.	No	
3.7	Longitudinal Studies	For longitudinal studies, state how many follow-ups were conducted, describe sample size at follow-up by group or condition, and discuss any loss to follow-up.	STORMS	If there is loss to follow-up, discuss the likelihood that drop-out is associated with exposures, treatments, or outcomes of interest.	No	
3.8	Matching	For matched studies, give matching criteria.	Modified STROBE	"Matched" refers to matching between comparable study participants as cases and controls or exposed / unexposed.<br><br>Indicate whether participants were individual or frequency matched and in what ratio were they matched (e.g. 1 case to 1 control).	NA	
3.9	Ethics	State the name of the institutional review board that approved the study and protocols, protocol number and date of approval, and procedures for obtaining informed consent from participants.	STORMS		Yes	Methods
4.0	Laboratory methods	State the laboratory/center where laboratory work was done.	STORMS	Provide a reference to complete lab protocols if previously published elsewhere such as on protocols.io. Note any modifications of lab protocols and the reason for protocol modifications.	Yes	Methods - CosmosID performed DNA extraction and shotgun metagenomic sequencing
4.1	Specimen collection	State the body site(s) sampled from and how specimens were collected.	MIxS: sample collection device or method; host body site	Use terms from the Uber-anatomy Ontology (https://www.ebi.ac.uk/ols/ontologies/uberon) to describe body sites in a standardized format.	Yes	Methods - stool swabs
4.2	Shipping	Describe how samples were stored and shipped to the laboratory.	STORMS	Include length of time from collection to receipt by the lab and if temperature control was used during shipping.	Yes	Methods
4.3	Storage	Describe how the laboratory stored samples, including time between collection and storage and any preservation buffers or refrigeration used.	STORMS	State where each procedure or lot of samples was done if not all in the same place.<br><br>Include reagent/lot/catalogue #s for storage buffers.	Yes	
4.4	DNA extraction	Provide DNA extraction method, including kit and version if relevant.	MIxS: nucleic acid extraction	If any DNA quantification methods were used prior to DNA amplification or at the pooling step of library preparation, state so here.	Yes	Methods- QIAGEN DNeasy PowerSoil Pro Kit
4.5	Human DNA sequence depletion or microbial DNA enrichment	Describe whether human DNA sequence depletion or enrichment of microbial or viral DNA was performed.	STORMS		Yes	Methods- human DNA contamination was removed during QC
4.6	Primer selection	Provide primer selection and DNA amplification methods as well as variable region sequenced (if applicable).	MIxS: pcr primers		NA	
4.7	Positive Controls	Describe any positive controls (mock communities) if used.	STORMS	If used, should be deposited under guidance provided in the 8.X items.	NA	
4.8	Negative Controls	Describe any negative controls if used.	STORMS	If used, should be deposited under guidance provided in the 8.X items.	NA	
4.9	Contaminant mitigation and identification	Provide any laboratory or computational methods used to control for or identify microbiome contamination from the environment, reagents, or laboratory.	STORMS	Includes filtering of reagents and other steps to minimize contamination. It is relevant to state whether the specimens of interest have low microbial load, which makes contamination especially relevant.	NA	
4.10	Replication	Describe any biological or technical replicates included in the sequencing, including which steps were replicated between them.	STORMS	Replication may be biological (redundant biological specimens) or technical (aliquots taken at different stages of analysis) and used in extraction, sequencing, preprocessing, and/or data analysis.	NA	
4.11	Sequencing strategy	Major divisions of strategy, such as shotgun or amplicon sequencing.	MIxS: sequencing method	For amplicon sequencing (for example, 16S variable region), state the region selected. State the model of sequencer used.	Yes	Methods - shotgun
4.12	Sequencing methods	State whether experimental quantification was used (QMP/cell count based, spike-in based) or whether relative abundance methods were applied.	STORMS	These include read length, sequencing depth per sample (average and minimum), whether reads are paired, and other parameters.	Yes	Methods- average and range of reads provided
4.13	Batch effects	Detail any blocking or randomization used in study design to avoid confounding of batches with exposures or outcomes. Discuss any likely sources of batch effects, if known.	STORMS	Sources of batch effects include sample collection, storage, library preparation, and sequencing and are commonly unavoidable in all but the smallest of studies.	No	

Number	Item	Recommendation	Item Source	Additional Guidance	Yes/No/NA	Comments or location in manuscript
4.14	Metatranscriptomics	Detail whether any mRNA enrichment was performed and whether/how retrotranscription was performed prior to sequencing. Provide size range of isolated transcripts. Describe whether the sequencing library was stranded or not. Provide details on sequencing methods and platforms.	STORMS	Provide details on any internal standards which may have been used as well as parameters and versions of any software or databases used.	No	
4.15	Metaproteomics	Detail which protease was used for digestion. Provide details on proteomic methods and platforms (e.g. LC-MS/MS, instrument type, column type, mass range, resolution, scan speed, maximum injection time, isolation window, normalised collision energy, and resolution).	STORMS	Provide details on any internal standards which may have been used as well as parameters and versions of any software or databases used.	No	
4.16	Metabolomics	Specify the analytic method used (such as nuclear magnetic resonance spectroscopy or mass spectrometry). For mass spectrometry, detail which fractions were obtained (polar and/or non polar) and how these were analyzed. Provide details on metabolomics methods and platforms (e.g. derivatization, instrument type, injection type, column type and instrument settings).	STORMS	Provide details on any internal standards which may have been used as well as parameters and versions of any software or databases used.	No	
5.0	Data sources/ measurement	For each non-microbiome variable, including the health condition, intervention, or other variable of interest, state how it was defined, how it was measured or collected, and any transformations applied to the variable prior to analysis.	MIxS: host disease status	State any sources of potential bias in measurements, for example multiple interviewers or measurement instruments, and whether these potential biases were assessed or accounted for in study design.<br><br>Use terms from a standardized ontology such as the Experimental Factor Ontology (https://www.ebi.ac.uk/efo/) to describe variables of interest in a standardized format.	NA	
6.0	Research design for causal inference	Discuss any potential for confounding by variables that may influence both the outcome and exposure of interest. State any variables controlled for and the rationale for controlling for them.	STORMS	For causal inference, this item refers to describing the assumptions that would be required to draw causal inferences from observational data. See Vujkovic-Cvijin, I., Sklar, J., Jiang, L. et al. Host variables confound gut microbiota studies of human disease. Nature 587, 448–454 (2020). https://doi.org/10.1038/s41586-020-2881-9 for more details on confounding in observational microbiome studies.<br><br>For example, hypothesized confounders may be controlled for by multivariable adjustment. Consider using a directed acyclic graph (DAG) to describe your causal model and justify any variables controlled for. DAGs can be made using www.dagitty.net.	Yes	Methods - colonscopy prep polyethylene glycol ingestion by IBD subjects
6.1	Selection bias	Discuss potential for selection or survival bias.	STORMS	Selection bias can occur when some members of the target study population are more likely to be included in the study/final analytic sample than others. Some examples include survival bias (where part of the target study population is more likely to die before they can be studied), convenience sampling (where members of the target study population are not selected at random), and loss to follow-up (when probability of dropping out is related to one of the things being studied).	No	
7.0	Bioinformatic and Statistical Methods	Describe any transformations to quantitative variables used in analyses (e.g. use of percentages instead of counts, normalization, rarefaction, categorization).	STORMS	If a variable is analyzed using different transformations, state rationale for the transformation and for each analyses which version of the variable is used.<br><br>In case of any complex or multistep transformations, give enumerated instructions for reproducing those transformations.	Yes	described throughout Methods
7.1	Quality Control	Describe any methods to identify or filter low quality reads or samples.	MIxS: sequence quality check	If samples were excluded based on quality or read depth, list the criteria used, the number of samples excluded, and the final sample size after quality control.	Yes	Methods - followed QC that was described in Wallen et al. 2022 PMID: 36376318
7.2	Sequence analysis	Describe any taxonomic, functional profiling, or other sequence analysis performed.	MIxS: feature prediction; similarity search method		Yes	Methods- MetaPhlAn + HUMAnN

Number	Item	Recommendation	Item Source	Additional Guidance	Yes/No/NA	Comments or location in manuscript
7.3	Statistical methods	Describe all statistical methods.	Modified STROBE	Describe any statistical tests used, exploratory data analysis performed, dimension reduction methods/unsupervised analysis, alpha/beta metrics, and/or methods for adjusting for measurement bias.<br><br>If multiple statistical methods are possible, discuss why the methods used were selected.<br><br>If a multiple hypothesis testing correction method was used, describe the type of correction used.<br><br>State which taxonomic levels are analyzed.	Yes	Methods- see analysis in R
7.4	Longitudinal analysis	If the study is longitudinal, include a section that explicitly states what analysis methods were used (if any) to account for grouping of measurements by individual or patterns over time.	STORMS		NA	
7.5	Subgroup analysis	Describe any methods used to examine subgroups and interactions.	STROBE		NA	
7.6	Missing data	Explain how missing data were addressed.	STROBE	"Missing data" refers to participant measurements such as covariates, exposures, outcomes, or time points that should have been collected but were not, not to zeros in taxonomic abundance tables or data points not applicable to that observation.	Yes	One PD sample was missing almost all metadata, so this sample was exluded from all analyses entirely. Small pseudo-count added to zero counts prior to performing CLR-transformation for PCoA
7.7	Sensitivity analyses	Describe any sensitivity analyses.	STROBE		No	
7.8	Findings	State criteria used to select findings for reporting.	STORMS	For example, false discovery rate with total number of tests, effect size threshold, significance threshold, microbes of interest.	Yes	Methods- see analysis in R
7.9	Software	Cite all software (including read mapping software) and databases (including any used for taxonomic reference or annotating amplicons, if applicable) used. Include version numbers.	Modified STREGA	Installed packages, add-ons or libraries should be stated and cited in addition to the software used.<br><br>All parameters employed that differ from the default of that software/version should be provided.<br><br>This is in addition to, not a replacement for, publishing of code as outlined in the section Reproducible Research.	Yes	Methods + Full list of software packages used can be found in the supplementary Key Resource Table
8.0	Reproducible research	Make a statement about whether and how others can reproduce the reported analysis.	STORMS	Any protected information that has been excluded or provided under controlled access should be listed along with any relevant data access procedures. "On request from authors" is not sufficiently detailed; formal data access procedures and conditions should be defined.<br><br>If data are unavailable, state so clearly.<br><br>Consider using a specialized rubric for reproducible research (such as: https://mbio.asm.org/content/9/3/e00525-18.short).<br><br>Consider preregistering the study protocol (such as on osf.io or https://plos.org/open-science/preregistration/).	Yes	Methods- all data and code associated with this project are publicly available
8.1	Raw data access	State where raw data may be accessed including demultiplexing information.	STORMS	Robust, long-term databases such as those hosted by NCBI and EBI are preferred. If using a private repository, provide rationale.	Yes	raw sequences available in NCBI SRA BioProject PRJNA1096686
8.2	Processed data access	State where processed data may be accessed.	STORMS	Unfiltered data should be provided.<br><br>Robust, long-term databases such as those hosted by NCBI and EBI-EMBL are preferred. Repositories like zenodo (https://zenodo.org/) or publisso (https://www.publisso.de/en/working-for-you/doi-service/) can be used to provide a DOI and long-term storage for processed datasets, even those which cannot be published openly.	Yes	source data available in Zenodo doi: 10.5281/zenodo.10912505

Number	Item	Recommendation	Item Source	Additional Guidance	Yes/No/NA	Comments or location in manuscript
8.3	Participant data access	State where individual participant data such as demographics and other covariates may be accessed, and how they can be matched to the microbiome data.	STORMS	If re-categorized, transformed, or otherwise derived variables were used in the analysis, these variables or code for deriving them should be provided.<br><br>Examples of how participant data can be matched to microbiome data are: using the same set of anonymized identifiers, or using different anonymized identifiers but providing a map.<br><br>Provided data should be sufficient to independently replicate the current analysis.	Yes	Table 1 + demographics and metadata available in Zenodo source data doi: 10.5281/zenodo.10912505
8.4	Source code access	State where code may be accessed.	STORMS	If a standard or formalized workflow was employed, reference it here.	Yes	all code stored in GitHub- https://github.com/maevekrueger/UFPF_metagenomics
8.5	Full results	Provide full results of all analyses, in computer-readable format, in supplementary materials.	STORMS	For example, any fold-changes, p-values, or FDR values calculated, provided as a spreadsheet.<br><br>Use a machine-readable, plain-text format such as csv or tsv.	Yes	full data tables from analyses can be found in supplementary materials
**Results**						
9.0	Descriptive data	Give characteristics of study participants (e.g. dietary, demographic, clinical, social) and information on exposures and potential confounders.	STROBE	Typically reported in a table included in the paper or as a supplementary table. Indicate number of participants with missing data for each variable of interest.<br><br>This includes environmental and lifestyle factors that may affect the relationship between the microbiome and the condition of interest. Participant diet and medication use should be summarized, if known.<br><br>At minimum, age and sex of all participants should be summarized.	Yes	Table 1
10.0	Microbiome data	Report descriptive findings for microbiome analyses with all applicable outcomes and covariates.	STORMS	This includes measures of diversity as well as relative abundances. These descriptive findings should be reported both for the sample overall and for individual groups.	Yes	PCoAs - Fig 1 + Supp. Fig 1
10.1	Taxonomy	Identify taxonomy using standardized taxon classifications that are sufficient to uniquely identify taxa.	STORMS	If not using full taxonomic hierarchy, make sure it is clear whether names stated are species, genera, family, etc.<br><br>Italicize genus/species pairs. Consult journal guidelines or standardized references on taxonomic nomenclature. For instance, https://wwwnc.cdc.gov/eid/page/scientific-nomenclature	Yes	
10.2	Differential abundance	Report results of differential abundance analysis by the variable of interest and (if applicable) by time, clearly indicating the direction of change and total number of taxa tested.	STORMS	If there are more than two groups, include omnibus (multigroup) test results if applicable to the research question.<br><br>If applicable, reported effect sizes should include a measure of uncertainty such as the confidence interval.	Yes	
10.3	Other data types	Report other data analyzed--e.g. metabolic function, functional potential, MAG assembly, and RNAseq.	STORMS		Yes	functional potential
10.4	Other statistical analysis	Report any statistical data analysis not covered above.	STORMS	This could include subgroup analysis, sensitivity analyses, and cluster analysis.<br><br>Visualizations should be easily interpretable and colorblind-friendly. The caption and/or main text should provide a detailed description of visualizations for visually-impaired readers.	Results	Fig 1-2
**Discussion**						
11.0	Key results	Summarise key results with reference to study objectives	STROBE		Yes	

Number	Item	Recommendation	Item Source	Additional Guidance	Yes/No/NA	Comments or location in manuscript
12.0	Interpretation	Give a cautious overall interpretation of results considering objectives, limitations, multiplicity of analyses, results from similar studies, and other relevant evidence.	STROBE	Define or clarify any subjective terms such as "dominant," "dysbiosis," and similar words used in interpretation of results.<br><br>When interpreting the findings, consider how the interpretation of the findings may be summarized or quoted for the general public such as in press releases or news articles.<br><br>If causal language is used in the interpretation (such as "alters," "affects," "results in," "causes," or "impacts"), assumptions made for causal inference should be explicitly stated as part of 6.0 and 13.0.<br><br>Distinguish between function potential (ie inferred from metagenomics) and observed activity (ie metatranscriptomic, metabolomic, proteomic) if discussing microbial function.	Yes	
13.0	Limitations	Discuss limitations of the study, taking into account sources of potential bias or imprecision.	STROBE	Also consider limitations resulting from the methods (especially novel methods), the study design, and the sample size.	Yes	Discussion
13.1	Bias	Discuss any potential for bias to influence study findings.	STORMS	May include sampling method, representativeness of study participants, or potential confounding.	Yes	Dicussion
13.2	Generalizability	Discuss the generalisability (external validity) of the study results	STROBE	To what populations or other settings do you expect the conclusions to generalize?	Yes	Discussion- included use of larger PD and IBD datasets in this analysis to validate and expand upon findings of UFPF dataset
14.0	Ongoing/future work	Describe potential future research or ongoing research based on the study's findings.	STORMS	Yes	Yes	Discussion
**Other information**						
15.0	Funding	Give the source of funding and the role of the funders for the present study and, if applicable, for the original study on which the present article is based	STROBE		Yes	Acknowledgements
15.1	Acknowledgements	Include acknowledgements of those who contributed to the research but did not meet critera for authorship.	STORMS	For general guidelines on authorship, see http://www.icmje.org and https://www.elsevier.com/authors/journal-authors/policies-and-ethics/credit-author-statement	Yes	Acknowledgements
15.2	Conflicts of Interest	Include a conflicts of interest statement.	STORMS		Yes	Competing Interests
16.0	Supplements	Indicate where supplements may be accessed and what materials they contain.	STORMS		Yes	Key Resource Table; Supplementary Tables, Subject Questionnaires, STORMS Checklist
17.0	Supplementary data	Provide supplementary data files of results with for all taxa and all outcome variables analyzed. Indicate the taxonomic level of all taxa.	STORMS	Depending on the analysis performed, examples of the supplemental results included could be mean relative abundance, differential abundance, raw p-value, multiple hypothesis testing-adjusted p-values, and standard error.<br><br>All discussed taxa should include the taxonomic level (e.g. class, order, genus).	Yes	see supplementary tables

RESOURCE TYPE	RESOURCE NAME	SOURCE	IDENTIFIER (w/ RRID)	NEW/ REUSE	ADDITIONAL INFORMATION
Dataset	DNA quantification: DNA extraction		Data not available	new	This was outsourced to CosmosID Inc. Data was not shared with us.
Dataset	DNA quantification: library preparation and sequencing		Data not available	new	This was outsourced to CosmosID Inc. Data was not shared with us.
Dataset	DNA sequencing	NCBI SRA	https://www.ncbi.nlm.nih.gov/bioproject/1096686	new	BioProject: PRJNA1096686
Dataset	human reference genome	NCBI	https://www.ncbi.nlm.nih.gov/datasets/genome/GCF_000001405.39/	reuse	click Download
Dataset	Read counts	Zenodo	https://zenodo.org/doi/10.5281/zenodo.10912505	new	In the Source_data file on our Zenodo Repository, in Table 1 titled "UFPF_metadata", total read count after QC can be found in the column titled "Reads." Taxonomic raw counts that were computed from MetaPhlAn's relative abundance output and the total read count can be found in Table 3 titled "UFPF_metaphlan_counts"
Dataset	ChocoPhlAn data (vJun23)	Segatalab FTP / Github	http://cmprod1.cibio.unitn.it/biobakery4/metaphlan_databases	reuse	Followed the instructions installation and downloading the databases outlined here- https://github.com/biobakery/MetaPhlAn/wiki/MetaPhlAn-4#installation. The CHOCOPhlAn databases downloaded from the Segatalab website where those dated mpa_vJune23.
Dataset	UniRef90 reference data (v201901b)	Github	https://github.com/biobakery/humann?tab=readme-ov-file#installation-update	reuse	Under the section "Download a translated search database," followed the steps to download the full UniRef90 database (20.7GB reccomended) via $humann_databases --download uniref uniref90_diamond $INSTALL_LOCATION
Dataset	MetaCyc reference data (v24)	MetaCyc	https://metacyc.org/	reuse	This is included when you download and install HUMAnN - https://github.com/biobakery/humann?tab=readme-ov-file#installation-update
Dataset	Demographic data	Source Data Table 1 / Zenodo	https://zenodo.org/doi/10.5281/zenodo.10912505	new	Table 1 (UFPF_metadata) of the Source_Data file in the Zenodo repository displays the demographic and metadata for the UFPF dataset.

RESOURCE TYPE	RESOURCE NAME	SOURCE	IDENTIFIER (w/ RRID)	NEW/ REUSE	ADDITIONAL INFORMATION
Dataset	Subject questionnaire data	Source Data Table 1 / Zenodo	https://zenodo.org/doi/10.5281/zenodo.10912505	new	Table 1 (UFPF_metadata) of the Source_Data file in the Zenodo repository displays the demographic and metadata for the UFPF dataset.
Dataset	Wallen et al. data	Zenodo	https://zenodo.org/doi/10.5281/zenodo.7246184	reuse	Tables 5 - 8 of the Source Data in our project's Zenodo repository (doi/10.5281/zenodo.10912505) display metadata and additional metagenomic data from the Wallen PD dataset. We sourced this data from this publication's Source_Data_24Oct2022.xlsx in their Zenodo repository in the link provided under the IDENTIFIER column to the left.
Dataset	Human Microbiome Project 2 (HMP2) data	IBMDB	https://www.ibdmdb.org/results	reuse	Demographic and metadata was acquired by clicking “Download HMP2 Metadata” with the updated data being 2018-08-20. Due to the large age range of the HMP2 project, this dataset was filtered to include only subjects 40 years and older. Taxonomic data was downloaded by identifying Name “HMP2”, Week “2018.18”, Data type “Metagenomics(MGX)”, and clicking “products.” Then selecting “Merged Tables” and downloading the file titled “taxonomic_profiles.tsv.gz”. Functional pathway data was downloaded via the file titled “pathabundance.tsv.gz" within the Merged Tables tab.
Software/code	R code	Github	https://github.com/maevekrueger/UFPF_metagenomics	new	R 4.2.3
Software/code	Python code	Github	https://github.com/maevekrueger/UFPF_metagenomics/blob/main/Bioinformatic%20Processing%20of%20Sequences	new	3.11
Software/code	BBSplit	Source Forge	https://sourceforge.net/projects/bbmap/	reuse	BBSplit

RESOURCE TYPE	RESOURCE NAME	SOURCE	IDENTIFIER (w/ RRID)	NEW/ REUSE	ADDITIONAL INFORMATION
Software/code	ChocoPhlAn	Segatalab FTP	http://cmprod1.cibio.unitn.it/biobakery4/metaphlan_databases	reuse	ChocoPhlAn _CHOCOPhlAnSGB_202307
Software/code	Python	Python	https://www.python.org/downloads/release/python-360/; (RRID:SCR_008394)	reuse	3.11
Software/code	MetaPhlAn	Huttenhower Lab	https://huttenhower.sph.harvard.edu/metaphlan; (RRID:SCR_004915)	reuse	MetaPhlAn 4
Software/code	HUMAnN	Huttenhower Lab	https://huttenhower.sph.harvard.edu/humann; (RRID:SCR_014620)	reuse	HUMAnN 3.5
Software/code	MetaCyc	MetaCyc	https://metacyc.org/; (RRID:SCR_007778)	reuse	24
Software/code	ANCOM-BC2	Bioconductor	https://bioconductor.org/packages/release/bioc/vignettes/ANCOMBC/inst/doc/ANCOMBC2.html; (RRID:SCR_024901)	reuse	2
Software/code	ANCOM-BC	Bioconductor	https://www.bioconductor.org/packages/release/bioc/html/ANCOMBC.html	reuse	package that ANCOM-BC2 is contained in; 2.0.3
Software/code	R	R Project	https://www.r-project.org/; (RRID:SCR_001905)	reuse	4.2.3
Software/code	readxl	Comprehensive R Archive Network	https://cran.r-project.org/web/packages/readxl/index.html; (RRID:SCR_018083)	reuse	1.4.3
Software/code	openxlsx	Comprehensive R Archive Network	https://cran.r-project.org/web/packages/openxlsx/index.html; (RRID:SCR_019185)	reuse	4.2.5.2
Software/code	tidyverse	Comprehensive R Archive Network	https://cran.r-project.org/web/packages/tidyverse/index.html; (RRID:SCR_019186)	reuse	2.0.0
Software/code	ape	Comprehensive R Archive Network	https://cran.r-project.org/web/packages/ape/index.html; (RRID:SCR_017343)	reuse	5.7-1
Software/code	vegan	Comprehensive R Archive Network	http://cran.r-project.org/web/packages/vegan/index.html; (RRID:SCR_011950)	reuse	2.6-4
Software/code	pairwiseAdonis	Github	https://github.com/pmartinezarbizu/pairwiseAdonis	reuse	0.4.1

RESOURCE TYPE	RESOURCE NAME	SOURCE	IDENTIFIER (w/ RRID)	NEW/ REUSE	ADDITIONAL INFORMATION
Software/code	arsenal	Comprehensive R Archive Network	https://cran.r-project.org/web/packages/arsenal/index.html	reuse	3.6.3
Software/code	compositions	Comprehensive R Archive Network	https://cran.r-project.org/web/packages/compositions/index.html	reuse	2.0-6
Software/code	phyloseq	Bioconductor	https://www.bioconductor.org/packages/release/bioc/html/phyloseq.html; (RRID:SCR_013080)	reuse	1.44.0
Software/code	ggplot2	Comprehensive R Archive Network	https://cran.r-project.org/web/packages/ggplot2/index.html; (RRID:SCR_014601)	reuse	3.4.2
Software/code	ggVennDiagram	Comprehensive R Archive Network	https://cran.r-project.org/web/packages/ggVennDiagram/index.html	reuse	1.2.3
Software/code	ggbeeswarm	Comprehensive R Archive Network	https://cran.r-project.org/web/packages/ggbeeswarm/index.html	reuse	0.7.2
Software/code	ggpubr	Comprehensive R Archive Network	https://cran.r-project.org/web/packages/ggpubr/index.html; (RRID:SCR_021139)	reuse	0.6.0
Software/code	camcorder	Comprehensive R Archive Network	https://cran.r-project.org/web/packages/camcorder/index.html	reuse	0.1.0
Software/code	paletteer	Comprehensive R Archive Network	https://cran.r-project.org/web/packages/paletteer/index.html	reuse	1.5.0
Software/code	WGCNA	Comprehensive R Archive Network	https://cran.r-project.org/web/packages/WGCNA/index.html; (RRID:SCR_003302)	reuse	1.72-1
Software/code	afex	Comprehensive R Archive Network	https://cran.r-project.org/web/packages/afex/index.html; (RRID:SCR_022857)	reuse	1.3-0
Software/code	emmeans	Comprehensive R Archive Network	https://cran.r-project.org/web/packages/emmeans/index.html; (RRID:SCR_018734)	reuse	1.8.7

RESOURCE TYPE	RESOURCE NAME	SOURCE	IDENTIFIER (w/ RRID)	NEW/ REUSE	ADDITIONAL INFORMATION
Software/code	multcomp	Comprehensive R Archive Network	https://cran.r-project.org/web/packages/multcomp/index.html; (RRID:SCR_018255)	reuse	1.4-25
Software/code	kableExtra	Comprehensive R Archive Network	https://cran.r-project.org/web/packages/kableExtra/index.html	reuse	1.3.4
Software/code	dplyr	Comprehensive R Archive Network	https://cran.r-project.org/web/packages/dplyr/index.html; (RRID:SCR_016708)	reuse	1.0.10
Software/code	stringr	Comprehensive R Archive Network	https://cran.r-project.org/web/packages/stringr/index.html; (RRID:SCR_022813)	reuse	1.5.0
Software/code	broom	Comprehensive R Archive Network	https://cran.r-project.org/web/packages/broom/index.html	reuse	1.0.5
Software/code	reshape2	Comprehensive R Archive Network	https://cran.r-project.org/web/packages/reshape2/index.html; (RRID:SCR_022679)	reuse	1.4.4
Protocols	DNA Extraction		Not available		DNA extraction & library prep and sequencing were done by CosmosID Inc. The methods provided from CosmosID Inc. are available in our manuscript. If you would like further information regarding their procedures, you can find their contact information listed on their website: https://www.cosmosid.com/
Protocols	Library Preparation and Sequencing		Not available		

FOR OFFICE USE ONLY

Subject ID: ________

Please indicate if you or your relatives have any of the following conditions.

Condition	You	Biological Mother	Biological Father	Biological Brothers or Sisters	Biological Children
Neurological					
Multiple Sclerosis					
Myasthenia Gravis					
Guillain-Barré Syndrome					
CIDP					
Polymyositis					
Dermatomyositis					
Alzheimer's Disease					
Non-Alzhemeier's Dementia					
Endocrine					
Hypothyroidism (under-active)					
Hyperthyroidism (over-active)					
Hashimoto thyroiditis					
Grave's disease					
Diabetes mellitus (age started)					
Type 1					
Type 2					
Addison's Disease					
Autoimmune Adrenalitis					
Skin					
Vitiligo					
Scleroderma					
Pemphigus					
Psoriasis					
Gastrointestinal					
Crohn's Disease					
Ulcerative Colitis					
B12 deficiency					
Bones and Joints					
Rhematoid Arthritis					
Reiter's Syndrome					
Ankylosing Spondylitis					
Systemic					
Lupus (SLE)					
Sjogren's Syndrome					
Hematologic					
Hemolytic Anemia					
Aplastic Anemia					
Pernicious Anemia					
Antiphospholipid Syndrome					
Thrombocytopenia					
TTP					
ITP					
Other Autoimmune Disorders					
Cancer					
Specify Type					

FOR OFFICE USE ONLY Subject ID:________________

Medications

Name **Total Daily Dose**

1. ______________________________
2. ______________________________
3. ______________________________
4. ______________________________
5. ______________________________
6. ______________________________
7. ______________________________
8. ______________________________
9. ______________________________
10. ______________________________

FOR OFFICE USE ONLY Subject ID:_______________

Tobacco

Have you smoked at least 100 cigarettes (about 5 packs) in your entire lifetime? ☐Yes ☐No

During the time that you smoked, how much did you smoke on average, and for how many years? Check all that apply.

☐Less than ½ pack per day, for _________ years (*specify number of years*)

☐Equal to or more than ½ pack but less than 1 pack per day, for __________ years (*specify number of years*)

☐Equal to or more than 1 pack but less than 2 packs per day, for __________years (*specify number of years*)

☐Equal to or more than 2 packs per day, for __________years (*specify number of years*)

At what age did you begin smoking? ________________________

Are you still smoking? ☐ Yes ☐ No If not, at what age did you stop? ___________________

NSAIDs

Over the Counter NSAIDs

NSAIDs are non-steroidal anti-inflammatory drugs, like Ibuprofen, Motrin IB, Advil, and Aleve, which are commonly used for pain. Aspirin and acetaminophen like Tylenol are not NSAIDs.

How often do you (or did you) take **over the counter NSAIDS**, and for how many years? Check all that apply.

☐ Never

☐ Less than once a week, for _________ years (*specify number of years)*

☐ About one to four times a week, for _________ years (*specify number of years)*

☐ About five to ten times a week, for _________ years (*specify number of years)*

☐ More than 10 times a week, for _________ years (*specify number of years)*

FOR OFFICE USE ONLY Subject ID:________________

Prescription NSAIDs

Prescription NSAIDS include Anaprox (generic name: naproxen), Arthrotec (diclofenac sodium), Bextra (valdecoxib), Cataflam (diclofenac potassium), Celebrex (celecoxib), Clinoril (sulindac), Dolobid (diflunisal), EC-naprosy n (naproxen), Feldene (piroxicam), Indocin (indomethacin), Mobic (meloxicam), Motrin (ibuprofen), Naprelan (nalproxen controlled release), Naprosyn (naproxen), Ponstel (mefenamic acid), Relafen (nabumetone), Toradol (ketcrolactromethamine), Triisate (choline magnesium salicylate), Vioxx (rofecoxib), Voltaren (diclofenac sodium)

How often do you (or did you) take prescription NSAIDs, and for how many years? Check all that apply.

☐Never
☐Less than once a week, for __________ years (*specify number of years)*
☐About one to four times a week, for __________ years (*specify number of years)*
☐About five to ten times a week, for __________ years (*specify number of years)*
☐More than 10 times a week, for __________ years (*specify number of years)*

Caffeine

How much **caffeinated coffee** do you (or did you) drink, and for how many years? Check all that apply.
A cup is about 5 ounces, which is the size of a small Styrofoam cup or a china cup. A coffee mug is 2 cups.

☐Never
☐Less than 2 cups a week, for __________ years (*specify number of years*)
☐1-2 cups a day, for ____________ years (*specify number of years*)
☐3-5 cups a day, for ____________ years (*specify number of years*)
☐6 or more cups a day, for ____________ years (*specify number of years*)

At what age did you start drinking caffeinated coffee? _________
Are you still drinking caffeinated coffee? ☐Yes ☐ No If not, at what age did you stop? ________________

FOR OFFICE USE ONLY Subject ID:________________

How much **caffeinated tea** do you (or did you) drink, and for how many years? Check all that apply.
A cup is about 5 ounces, which is the size of a small Styrofoam cup or a china cup. A coffee mug is 2 cups.

- ☐ Never
- ☐ Less than 2 cups a week, for __________ years (*specify number of years*)
- ☐ 1-2 cups a day, for ____________ years (*specify number of years*)
- ☐ 3-5 cups a day, for ____________ years (*specify number of years*)
- ☐ 6 or more cups a day, for ____________ years (*specify number of years*)

At what age did you start drinking caffeinated tea? _________
Are you still drinking caffeinated tea? ☐ Yes ☐ No If not, at what age did you stop? ________________

How much **caffeinated soda** do you (or did you) drink, and for how many years? Check all that apply.
A can of soda is 12 oz.

- ☐ Never
- ☐ Less than 2 cups a week, for __________ years (*specify number of years*)
- ☐ 1-2 cups a day, for ____________ years (*specify number of years*)
- ☐ 3-5 cups a day, for ____________ years (*specify number of years*)
- ☐ 6 or more cups a day, for ____________ years (*specify number of years*)

At what age did you start drinking caffeinated soda? _________
Are you still drinking caffeinated soda? ☐ Yes ☐ No If not, at what age did you stop? ________________

Head Injury

Have you ever had a head injury that caused loss of consciousness or required medical care? ☐ Yes ☐ No (If no, go to Ethnic origins section)
How many times in your life have you had such a head injury? _______ times
How old were you when you had your first head injury? _________ years old
How old were you when you had your last head injury? _________ years old

FOR OFFICE USE ONLY Subject ID:_______________

Ethnic Origins

From what countries did your paternal ancestors immigrate to the United States? ____________________

From what countries did your maternal ancestors immigrate to the United States? ____________________

Are you Hispanic or Latino? ☐ Yes ☐ No

What race do you most identify yourself with:

☐ American Indian/Alaskan Native

☐ Asian

☐ Native Hawaiian or other Pacific Islander

☐ Black or African American

☐ White

☐ More than one race

If you are from a particular religious lineage, such as Jewish, Amish, etc., please specify.
This is a question of genetic lineage, not personal preference: ____________________

Immune System Related Events

Any recent immunizations (including annual flu)? Please provide type and date.

Have you had a fever or a known infection in the past 30 days? Please specify.

Have you been hospitalized in the past 30 days? Please specify.

Have you had surgery in the past 30 days? Please specify.

Activities of Daily Living Scale

Patient: ____________ Date: ____________ Time Point: B 2/3w 3m 1y

Your Name: ____________ Relationship to Participant: ____________

Please circle the description under each heading which best characterizes the patient's behavior over the *past several weeks*.

Instrumental Activities Scale

Ability to Use the Telephone

(1) Operates telephone on own initiative, looks up and dials numbers, etc.
(2) Answers telephone; dials a few well-known numbers, but does not look up or dial less-frequently used numbers without assistance.
(3) Does not use telephone at all.

Shopping

(1) Takes care of all shopping needs independently (i.e. goes to the store, selects needed items, pays for items and brings them home).
(2) Shops independently for small purchases.
(3) Completely unable to shop alone.

Food Preparation

(1) Plans, prepares and serves adequate meals independently; manages the stove without help.
(2) Prepares adequate meals if supplied with ingredients; heats, serves and prepares meals but does not maintain an adequate diet.
(3) Needs to have meals prepared and served.

Housekeeping

(1) Maintains house (house-cleaning, vacuum-cleaning, washes floors, etc.) alone or with occasional assistance
(2) Performs light daily tasks such as dish-washing and bed-making, but cannot maintain an acceptable level of cleanliness.
(3) Does not participate in any housekeeping tasks.

Laundry

(1) Does personal laundry completely.
(2) Launders small items, washes some clothes by hand, needs help with major laundry.
(3) All laundry must be done by others.

Mode of Transportation

(1) Travels independently on public transportation, drives own car, or arranges own travel via taxi.
(2) Travels on public transportation, taxi, or automobile when assisted or accompanied by another.
(3) Does not travel at all.

Medications

(1) Is responsible for taking medication in correct dosages at correct time
(2) Takes responsibility of medication if it is prepared in advance in separate dosage.
(3) Is not capable of dispensing own medication.

Ability to Handle Finances

(1) Manages financial matters independently (budgets, writes checks, pays rent, bills, collects and keeps track of income, etc.).
(2) Manages day-to-day purchases but needs help with banking, major purchases, etc.
(3) Incapable of handling money.

Physical Self Maintenance Scale

Toilet

(1) Cares for self at toilet completely, no incontinence
(2) Needs to be reminded, needs help cleaning self or redressing, or has rare (weekly at most) accidents.
(3) Soiling of wetting more than once a week or total incontinence.

Feeding

(1) Eats without assistance.
(2) East with mild to moderate assistance at mealtimes and/or with special preparation of food or help in cleaning up.
(3) Does not feel self at all

Dressing

(1) Dresses, undresses and selects clothes from wardrobe without help.
(2) Needs minor to moderate assistance in dressing and/or in the selection of clothes.
(3) Unable to dress self.

Hair Grooming

(1) Washes and combs/brushes hair independently.
(2) Needs moderate and regular assistance in grooming or does not wash/comb hair unless told.
(3) Caregiver must wash and comb hair.

Dental Hygiene

(1) Brushes teeth independently and does not require to be reminded.
(2) Needs moderate and regular supervision or assistance in brushing teeth (needs to be reminded to brush teeth; needs help gathering all necessary objects, etc.)
(3) Can not brush teeth without complete assistance.

Nail Care

(1) Cares for nails (cleans and clips) regularly and without assistance.
(2) Must be reminded to care for nails or requires some assistance in cutting nails.
(3) Relies on others for total nail grooming care.

Bathing

(1) Bathes self (tub, shower, sponge bath) without help.
(2) Needs supervision or assistance in bathing.
(3) Does not wash self.

BDI - II

Instructions: This questionnaire consists of 21 groups of statements. Please read each group of statements carefully. And then pick out the one statement in each group that best describes the way you have been feeling during the past two weeks, including today. Circle the number beside the statement you have picked. If several statements in the group seem to apply equally well, circle the highest number for that group. Be sure that you do not choose more than one statement for any group, including Item 16 (Changes in Sleeping Pattern) or Item 18 (Changes in Appetite).

1. Sadness
   0. I do not feel sad.
   1. I feel sad much of the time.
   2. I am sad all the time.
   3. I am so sad or unhappy that I can't stand it.

2. Pessimism
   0. I am not discouraged about my future.
   1. I feel more discouraged about my future than I used to.
   2. I do not expect things to work out for me.
   3. I feel my future is hopeless and will only get worse.

3. Past Failure
   0. I do not feel like a failure.
   1. I have failed more than I should have.
   2. As I look back, I see a lot of failures.
   3. I feel I am a total failure as a person.

4. Loss of Pleasure
   0. I get as much pleasure as I ever did from the things I enjoy.
   1. I don't enjoy things as much as I used to.
   2. I get very little pleasure from the things I used to enjoy.
   3. I can't get any pleasure from the things I used to enjoy.

5. Guilty Feelings
   0. I don't feel particularly guilty.
   1. I feel guilty over many things I have done or should have done.
   2. I feel quite guilty most of the time.
   3. I feel guilty all of the time.

6. Punishment Feelings
   0. I don't feel I am being punished.
   1. I feel I may be punished.
   2. I expect to be punished.
   3. I feel I am being punished.

7. Self-Dislike
   0. I feel the same about myself as ever.
   1. I have lost confidence in myself.
   2. I am disappointed in myself.
   3. I dislike myself.

8. Self-Criticalness

0. I don't criticize or blame myself more than usual.
1. I am more critical of myself than I used to be.
2. I criticize myself for all of my faults.
3. I blame myself for everything bad that happens.

9. Suicidal Thoughts or Wishes

0. I don't have any thoughts of killing myself.
1. I have thoughts of killing myself, but I would not carry them out.
2. I would like to kill myself.
3. I would kill myself if I had the chance.

10. Crying

0. I don't cry anymore than I used to.
1. I cry more than I used to.
2. I cry over every little thing.
3. I feel like crying, but I can't.

11. Agitation

0. I am no more restless or wound up than usual.
1. I feel more restless or wound up than usual.
2. I am so restless or agitated, it's hard to stay still.
3. I am so restless or agitated that I have to keep moving or doing something.

12. Loss of Interest

0. I have not lost interest in other people or activities.
1. I am less interested in other people or things than before.
2. I have lost most of my interest in other people or things.
3. It's hard to get interested in anything.

13. Indecisiveness

0. I make decisions about as well as ever.
1. I find it more difficult to make decisions than usual.
2. I have much greater difficulty in making decisions than I used to.
3. I have trouble making any decisions.

14. Worthlessness

0. I do not feel I am worthless.
1. I don't consider myself as worthwhile and useful as I used to.
2. I feel more worthless as compared to others.
3. I feel utterly worthless.

15. Loss of Energy

0. I have as much energy as ever.
1. I have less energy than I used to have.
2. I don't have enough energy to do very much.
3. I don't have enough energy to do anything.

16. Changes in Sleeping Pattern

0. I have not experienced any change in my sleeping.
1a I sleep somewhat more than usual.
1b I sleep somewhat less than usual.
2a I sleep a lot more than usual.
2b I sleep a lot less than usual.
3a I sleep most of the day.
3b I wake up 1-2 hours early and can't get back to sleep.

17. Irritability

0. I am not more irritable than usual.
1. I am more irritable than usual.
2. I am much more irritable than usual.
3. I am irritable all the time.

18. Changes in Appetite

0. I have not experienced any change in my appetite.
1a My appetite is somewhat less than usual.
1b My appetite is somewhat greater than usual.
2a My appetite is much less than before.
2b My appetite is much greater than usual.
3a I have no appetite at all.
3b I crave food all the time.

19. Concentration Difficulty

0. I can concentrate as well as ever.
1. I can't concentrate as well as usual.
2. It's hard to keep my mind on anything for very long.
3. I find I can't concentrate on anything.

20. Tiredness or Fatigue

0. I am no more tired or fatigued than usual.
1. I get more tired or fatigued more easily than usual.
2. I am too tired or fatigued to do a lot of the things I used to do.
3. I am too tired or fatigued to do most of the things I used to do.

21. Loss of Interest in Sex

0. I have not noticed any recent change in my interest in sex.
1. I am less interested in sex than I used to be.
2. I am much less interested in sex now.
3. I have lost interest in sex completely.

Total Score: _______

THE PSYCHOLOGICAL CORPORATION
Harcourt Brace & Company
Copyright 1996, by Aaron T. Beck. All rights reserved.

PDBP
Parkinson's Disease
Biomarkers Program

* **Required fields**

Modified Schwab and England Activities of Daily Living Scale

*Name of Site: ____________________	*Type of Visit: ____________________<br>e.g. Screening, Baseline, 6 months, 12 months, 18 months, 24 months, 30 months, 36 months, 42 months, 48 months, 54 months, 60 months.
*Date of Visit: ____________________	*GUID: ________________________
*Age of Subject (years and months): ______________	Subject ID: ______________________

*Score: __________ (Number between 1-100)

100% – Completely independent. Able to do all chores without slowness, difficulty or impairment. Essentially normal. Unaware of any difficulty.

90% – Completely independent. Able to do all chores with some degree of slowness, difficulty and impairment. Might take twice as long. Beginning to be aware of difficulty.

80% – Completely independent in most chores. Takes twice as long. Conscious of difficulty and slowness.

70% – Not completely independent. More difficulty with some chores. Three to four times as long in some. Must spend a large part of the day with chores.

60% – Some dependency. Can do most chores, but exceedingly slowly and with much effort. Errors; some impossible.

50% – More dependent. Help with half, slower, et cetera. Difficulty with everything.

40% – Very dependent. Can assist with all chores, but few alone.

30% – With effort, now and then does a few chores alone or begins alone. Much help needed.

20% – Nothing alone. Can be a slight help with some chores. Severe invalid.

10% – Total dependent, helpless. Complete invalid.

0% – Vegetative functions such as swallowing, bladder and bowel functions are not functioning. Bed-ridden.

MONTREAL COGNITIVE ASSESSMENT (MOCA)
Version 7.1 Original Version

NAME :
Education :
Sex :
Date of birth :
DATE :

VISUOSPATIAL / EXECUTIVE

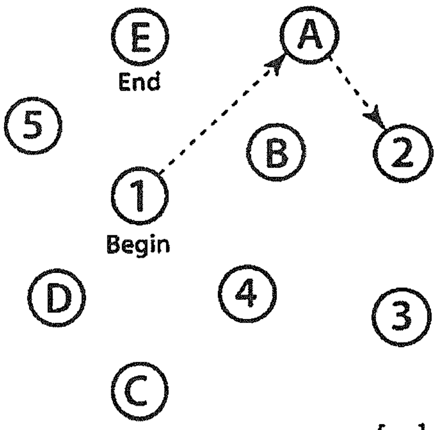

[ ]

Copy cube

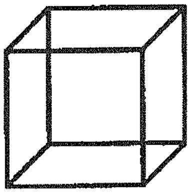

[ ]

Draw CLOCK (Ten past eleven)
(3 points)

[ ] Contour [ ] Numbers [ ] Hands

POINTS ___/5

NAMING

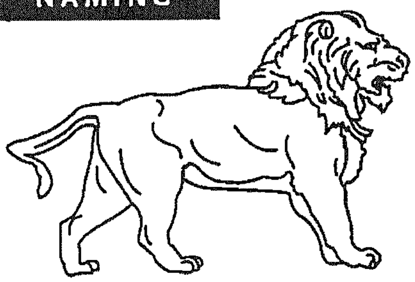

[ ]

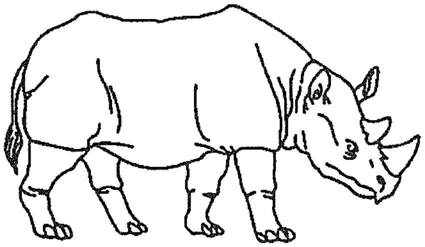

[ ]

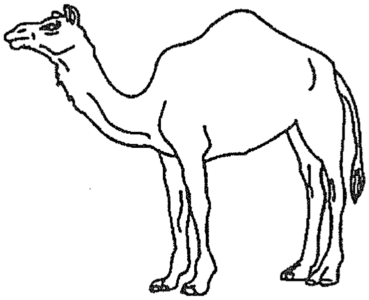

[ ]

___/3

MEMORY

Read list of words, subject must repeat them. Do 2 trials, even if 1st trial is successful.
Do a recall after 5 minutes.

	FACE	VELVET	CHURCH	DAISY	RED
1st trial					
2nd trial					

No points

ATTENTION

Read list of digits (1 digit/ sec.).

Subject has to repeat them in the forward order [ ] 2 1 8 5 4
Subject has to repeat them in the backward order [ ] 7 4 2

___/2

Read list of letters. The subject must tap with his hand at each letter A. No points if ≥ 2 errors

[ ] F B A C M N A A J K L B A F A K D E A A A J A M O F A A B

___/1

Serial 7 subtraction starting at 100 [ ] 93 [ ] 86 [ ] 79 [ ] 72 [ ] 65

4 or 5 correct subtractions: **3 pts**, 2 or 3 correct: **2 pts**, 1 correct: **1 pt**, 0 correct: **0 pt**

___/3

LANGUAGE

Repeat : I only know that John is the one to help today. [ ]
The cat always hid under the couch when dogs were in the room. [ ]

___/2

Fluency / Name maximum number of words in one minute that begin with the letter F [ ] _____ (N ≥ 11 words)

___/1

ABSTRACTION

Similarity between e.g. banana - orange = fruit [ ] train – bicycle [ ] watch - ruler

___/2

DELAYED RECALL

Has to recall words WITH NO CUE	FACE [ ]	VELVET [ ]	CHURCH [ ]	DAISY [ ]	RED [ ]	Points for UNCUED recall only
Optional — Category cue						
Optional — Multiple choice cue						

___/5

ORIENTATION

[ ] Date [ ] Month [ ] Year [ ] Day [ ] Place [ ] City

___/6

© Z.Nasreddine MD www.mocatest.org Normal ≥26 / 30

TOTAL ___/30

Add 1 point if ≤ 12 yr edu

Administered by: ______________________

FOR OFFICE USE ONLY Subject ID:_______________

Confidential Environmental and Family History Questionnaire for PD Studies

Today's Date: ________________________

Personal Information

Name: ________________________ Birthdate: ________________ Sex: M☐ F☐

Address: ____

Phone (H): ________________ Phone (W): ________________ Email: ________________

Have you ever been diagnosed with, or suspected to have Parkinson's disease or parkinsonism? ☐ Yes ☐ No

If you answered **yes** to the above question:

What was your first symptom? ________________________

At what age did you notice the first symptom? ______________

If seen by a physician, at what age were you diagnosed? ______ Name of Neurologist (City, State):______________________

If this form is completed by someone other than the subject/patient:

Name of person completing form: ________________________________

Relationship to subject/patient: ________________ Number of years you have known the subject/patient: ______________

Address: ____

Phone (H): ________________ Phone (W): ________________ Email: ________________

Parkinson's Disease Screening Questionnaire (Control)

Site Name: ____________________

Subject ID: ______________

1. Do you have trouble arising from a chair? ☐YES ☐NO
2. Is your handwriting smaller than it once was? ☐YES ☐NO
3. Do people tell you that your voice is softer than it once was? ☐YES ☐NO
4. Is your balance poor? ☐YES ☐NO
5. Do your feet ever seem to get stuck to the floor? ☐YES ☐NO
6. Do people tell you that your face seems less expressive than it once did? ☐YES ☐NO
7. Do your arms and legs shake? ☐YES ☐NO
8. Do you have trouble buttoning buttons? ☐YES ☐NO
9. Do you shuffle your feet and / or take tiny steps when you walk? ☐YES ☐NO
10. Has anyone ever told you that you have Parkinson's disease? ☐YES ☐NO
11. Have you ever taken levodopa or Sinemet? ☐YES ☐NO

Rocca et al., J Clin Epidemiol Vol. 51, No. 6, pp. 517 – 523, 1998

Unified PARKINSON Disease Rating Scale (UPDRS)

(AAN, 1995)

The UPDRS is a rating tool to follow the longitudinal course of Parkinson's Disease. It is made up of the 1)Mentation, Behavior, and Mood, 2)ADL and 3)Motor sections. These are evaluated by interview. Some sections require multiple grades assigned to each extremity.

I. Mentation, Behavior, Mood

o *Intellectual Impairment*
0-none
1-mild (consistent forgetfulness with partial recollection of events with no other difficulties)
2-moderate memory loss with disorientation and moderate difficulty handling complex problems
3-severe memory loss with disorientation to time and often place, severe impairment with problems
4-severe memory loss with orientation only to person, unable to make judgments or solve problems

o *Thought Disorder*
0-none
1-vivid dreaming
2-"benign" hallucination with insight retained
3-occasional to frequent hallucination or delusions without insight, could interfere with daily activities
4-persistent hallucination, delusions, or florid psychosis.

o *Depression*
0-not present
1-periods of sadness or guilt greater than normal, never sustained for more than a few days or a week
2-sustained depression for >1 week
3-vegetative symptoms (insomnia, anorexia, abulia, weight loss)
4-vegetative symptoms with suicidality

o *Motivation/Initiative*
0-normal
1-less of assertive, more passive
2-loss of initiative or disinterest in elective activities
3-loss of initiative or disinterest in day to say (routine) activities
4-withdrawn, complete loss of motivation

II. Activities of Daily Living

o *Speech*
0-normal
1-mildly affected, no difficulty being understood
2-moderately affected, may be asked to repeat
3-severely affected, frequently asked to repeat
4-unintelligible most of time

o *Salivation*
0-normal
1-slight but noticeable increase, may have nighttime drooling
2-moderately excessive saliva, hay minimal drooling
3-marked drooling

o *Swallowing*
0-normal
1-rare choking
2-occasional choking
3-requires soft food
4-requires NG tube or G-tube

o *Handwriting*
0-normal
1-slightly small or slow
2-all words small but legible
3-severely affected, not all words legible
4-majority illegible

o *Cutting Food/Handing Utensils*
0-normal
1-somewhat slow and clumsy but no help needed
2-can cut most foods, some help needed
3-food must be cut, but can feed self
4-needs to be fed

o *Dressing*
0-normal
1-somewhat slow, no help needed
2-occasional help with buttons or arms in sleeves
3-considerable help required but can do something alone
4-helpless

o *Hygiene*
0-normal
1-somewhat slow but no help needed
2-needs help with shower or bath or very slow in hygienic care
3-requires assistance for washing, brushing teeth, going to bathroom
4-helpless

o *Turning in Bed/ Adjusting Bed Clothes*
0-normal
1-somewhat slow no help needed
2-can turn alone or adjust sheets but with great difficulty
3-san initiate but not turn or adjust alone
4-helpless

- o *Falling-Unrelated to Freezing*
  - 0-none
  - 1-rare falls
  - 2-occasiona, less than one per day
  - 3-average of once per day
  - 4->1 per day
- o *Freezing When Walking*
  - 0-normal
  - 1-rare, may have start hesitation
  - 2-occasional falls from freezing,
  - 3-frequent freezing, occasional falls
  - 4-frequent falls from freezin
- o *Walking*
  - 0-normal
  - 1-mild difficulty, day drag legs or decrease arm swing
  - 2-moderate difficultly requires no assist
  - 3-severe disturbance requires assistance
  - 4-cannot walk at all even with assist
- o *Tremor*
  - 0-absent
  - 1-slight and infrequent, not bothersome to patient
  - 2-moderate, bothersome to patient
  - 3-severe, interfere with many activities
  - 4-marked, interferes with many activities
- o *Sensory Complaints Related to Parkinsonism*
  - 0-none
  - 1-occasionally has numbness, tingling, and mild aching
  - 2-frequent, but not distressing
  - 3-frequent painful sensation
  - 4-excruciating pain

III. Motor Exam

- o *Speech*
  - 0-normal
  - 1-slight loss of expression, diction,volume
  - 2-monotone, slurred but understandable, mod. impaired
  - 3-marked impairment, difficult to understand
  - 4-unintelligible
- o Facial Expression
  - 0-Normal
  - 1-slight hypomymia, could be poker face
  - 2-slight but definite abnormal diminution in expression
  - 3-mod. hypomimia, lips parted some of time
  - 4-masked or fixed face, lips parted 1/4 of inch or more with complete loss of expression
- o *Tremor at Rest*
  - + Face
    - 0-absent
    - 1-slight and infrequent
    - 2-mild and present most of time
    - 3-moderate and present most of time
    - 4-marked and present most of time
  - + Right Upper Extremity (RUE)
    - 0-absent
    - 1-slight and infrequent
    - 2-mild and present most of time
    - 3-moderate and present most of time
    - 4-marked and present most of time
  - + LUE
    - 0-absent
    - 1-slight and infrequent
    - 2-mild and present most of time
    - 3-moderate and present most of time
    - 4-marked and present most of time
  - + RLE
    - 0-absent
    - 1-slight and infrequent
    - 2-mild and present most of time
    - 3-moderate and present most of time
    - 4-marked and present most of time
  - + LLE
    - 0-absent
    - 1-slight and infrequent
    - 2-mild and present most of time
    - 3-moderate and present most of time
    - 4-marked and present most of time
- o *Action or Postural Tremor*
  - + RUE
    - 0-absent
    - 1-slight, present with action
    - 2-moderate, present with action
    - 3-moderate present with action and posture holding
    - 4-marked, interferes with feeding
  - + LUE
    - 0-absent
    - 1-slight, present with action
    - 2-moderate, present with action
    - 3-moderate present with action and posture holding
    - 4-marked, interferes with feeding
- o *Rigidity*
  - + Neck
    - 0-absent
    - 1-slight or only with activation
    - 2-mild/moderate
    - 3-marked, full range of motion
    - 4-severe
  - + RUE
    - 0-absent
    - 1-slight or only with activation
    - 2-mild/moderate
    - 3-marked, full range of motion
    - 4-severe
  - + LUE
    - 0-absent
    - 1-slight or only with activation
    - 2-mild/moderate
    - 3-marked, full range of motion
    - 4-severe
  - + RLE
    - 0-absent
    - 1-slight or only with activation
    - 2-mild/moderate
    - 3-marked, full range of motion
    - 4-severe
  - + LLE
    - 0-absent

1-slight or only with activation
2-mild/moderate
3-marked, full range of motion
4-severe

o *Finger taps*

+ Right
0-normal
1-mild slowing, and/or reduction in amp.
2-moderate impaired. Definite and early fatiguing, may have occasional arrests
3-severely impaired. Frequent hesitations and arrests.
4-can barely perform

+ Left
0-normal
1-mild slowing, and/or reduction in amp.
2-moderate impaired. Definite and early fatiguing, may have occasional arrests
3-severely impaired. Frequent hesitations and arrests.
4-can barely perform

o *Hand Movements (open and close hands in rapid succession)*

+ Right
0-normal
1-mild slowing, and/or reduction in amp.
2-moderate impaired. Definite and early fatiguing, may have occasional arrests
3-severely impaired. Frequent hesitations and arrests.
4-can barely perform

+ Left
0-normal
1-mild slowing, and/or reduction in amp.
2-moderate impaired. Definite and early fatiguing, may have occasional arrests
3-severely impaired. Frequent hesitations and arrests.
4-can barely perform

o *Rapid Alternating Movements (pronate and supinate hands)*

+ Right
0-normal
1-mild slowing, and/or reduction in amp.
2-moderate impaired. Definite and early fatiguing, may have occasional arrests
3-severely impaired. Frequent hesitations and arrests.
4-can barely perform

+ Left
0-normal
1-mild slowing, and/or reduction in amp.
2-moderate impaired. Definite and early fatiguing, may have occasional arrests
3-severely impaired. Frequent hesitations and arrests.
4-can barely perform

o *Leg Agility (tap heel on ground, amp should be 3 inches)*

+ Right
0-normal
1-mild slowing, and/or reduction in amp.
2-moderate impaired. Definite and early fatiguing, may have occasional arrests
3-severely impaired. Frequent hesitations and arrests.
4-can barely perform

+ Left
0-normal
1-mild slowing, and/or reduction in amp.
2-moderate impaired. Definite and early fatiguing, may have occasional arrests
3-severely impaired. Frequent hesitations and arrests.
4-can barely perform

o *Arising From Chair (pt. arises with arms folded across chest)*
0-normal
1-slow, may need more than one attempt
2-pushes self up from arms or seat
3-tends to fall back, may need multiple tries but can arise without assistance
4-unable to arise without help

o *Posture*
0-normal erect
1-slightly stooped, could be normal for older person
2-definitely abnormal, mod. stooped, may lean to one side
3-severely stooped with kyphosis
4-marked flexion with extreme abnormality of posture

o *Gait*
0-normal
1-walks slowly, may shuffle with short steps, no festination or propulsion
2-walks with difficulty, little or no assistance, some festination, short steps or propulsion
3-severe disturbance, frequent assistance
4-cannot walk

o *Postural Stability (retropulsion test)*
0-normal
1-recovers unaided
2-would fall if not caught
3-falls spontaneously
4-unable to stand

o *Body Bradykinesia/ Hypokinesia*
0-none
1-minimal slowness, could be normal, deliberate character
2-mild slowness and poverty of movement, definitely abnormal, or dec. amp. of movement
3-moderate slowness, poverty, or small amplitude
4-marked slowness, poverty, or amplitude
